# ANO1 Expression Orchestrates p27Kip1/MCL1-Mediated Signaling in Head and Neck Squamous Cell Carcinoma

**DOI:** 10.3390/cancers13051170

**Published:** 2021-03-09

**Authors:** Artemis Filippou, Henna Pehkonen, Piia-Riitta Karhemo, Juho Väänänen, Anni I. Nieminen, Juha Klefström, Reidar Grénman, Antti A. Mäkitie, Heikki Joensuu, Outi Monni

**Affiliations:** 1 Applied Tumor Genomics Research Program, Faculty of Medicine, University of Helsinki, 00014 Helsinki, Finland; artemis.filippou@helsinki.fi (A.F.); henna.pehkonen@helsinki.fi (H.P.); piia-riitta.karhemo@helsinki.fi (P.-R.K.); juho.vaananen@helsinki.fi (J.V.); 2 Translational Cancer Medicine Research Program and Medicum, Faculty of Medicine, University of Helsinki, 00014 Helsinki, Finland; anni.nieminen@helsinki.fi; 3 Finnish Cancer Institute, FICAN South Helsinki University Hospital, Faculty of Medicine, University of Helsinki, 00014 Helsinki, Finland; juha.klefstrom@helsinki.fi; 4 Department of Otorhinolaryngology-Head and Neck Surgery, University of Turku and Turku University Hospital, 20520 Turku, Finland; reidar.grenman@utu.fi; 5 Department of Otorhinolaryngology-Head and Neck Surgery, University of Helsinki and Helsinki University Hospital, 00130 Helsinki, Finland; antti.makitie@helsinki.fi; 6 Research Program in Systems Oncology, Faculty of Medicine, University of Helsinki, 00014 Helsinki, Finland; 7 Department of Oncology, University of Helsinki and Helsinki University Hospital, 00290 Helsinki, Finland; heikki.joensuu@hus.fi; 8 Department of Oncology, Clinicum, Faculty of Medicine, University of Helsinki, 00014 Helsinki, Finland

**Keywords:** ANO1, head and neck cancer, p27^Kip1^, MCL1, intrinsic apoptosis, cell cycle, targeted therapy, Ani9-5f, AZD-5991

## Abstract

**Simple Summary:**

Our aim was to elucidate the molecular mechanisms of how ANO1 contributes to oncogenic processes in squamous cell carcinoma of the head and neck (HNSCC). We explored transcriptional programs influenced by ANO1 knockdown in patient-derived UT-SCC cell lines with 11q13 amplification and ANO1 overexpression. ANO1 depletion led to downregulation of broad pro-survival BCL2 family protein members, including MCL1, and simultaneously induced upregulation of the cell cycle inhibitor p27^Kip1^ and its redistribution from the cytoplasm into the nucleus in the studied HNSCC cells. Gene set enrichment analysis highlighted pathways associated with perturbed cell cycle and apoptosis in the ANO1-depleted samples. Silencing of ANO1 and application of an ANO1-targeting small-molecule inhibitor led to ANO1 degradation and reduction of cell viability. These findings suggest that ANO1 has drug target potential that deserves further evaluation in preclinical in vivo models.

**Abstract:**

Head and neck squamous cell carcinoma (HNSCC) is a heterogeneous group of tumors that derive from the mucosal epithelium of the upper aerodigestive tract and present high mortality rate. Lack of efficient targeted-therapies and biomarkers towards patients’ stratification are caveats in the disease treatment. Anoctamin 1 (*ANO1*) gene is amplified in 30% of HNSCC cases. Evidence suggests involvement of ANO1 in proliferation, migration, and evasion of apoptosis; however, the exact mechanisms remain elusive. Aim of this study was to unravel the ANO1-dependent transcriptional programs and expand the existing knowledge of ANO1 contribution to oncogenesis and drug response in HNSCC. We cultured two HNSCC cell lines established from primary tumors harboring amplification and high expression of ANO1 in three-dimensional collagen. Differential expression analysis of ANO1-depleted HNSCC cells demonstrated downregulation of MCL1 and simultaneous upregulation of p27^Kip1^ expression. Suppressing ANO1 expression led to redistribution of p27^Kip1^ from the cytoplasm to the nucleus and associated with a cell cycle arrested phenotype. ANO1 silencing or pharmacological inhibition resulted in reduction of cell viability and ANO1 protein levels, as well as suppression of pro-survival BCL2 family proteins. Collectively, these data provide insights of ANO1 involvement in HNSCC carcinogenesis and support the rationale that ANO1 is an actionable drug target.

## 1. Introduction

Head and neck squamous cell carcinoma (HNSCC) consists of an etiologically and clinically heterogeneous group of tumors, located in the upper aerodigestive tract [1]. The estimated global annual incidence of head and neck cancer is 890,000 new cases leading to about 450,000 deaths [2]. The most commonly identified causal factors are tobacco and alcohol consumption [3], and human papillomavirus infection [4]. HNSCCs harbor a variety of genetic aberrations [5]. The standard primary treatment modalities are surgery and radiotherapy with or without chemotherapy. Cetuximab, a monoclonal antibody that binds to the epidermal growth factor receptor (EGFR) and the immune checkpoint inhibitors nivolumab and pembrolizumab have more recently been approved for the treatment of HNSCC [6]. Locally recurrent and advanced HNSCC remain, however, challenging to treat and the development of novel treatments is a high priority [7].

Gene encoding anoctamin-1 (ANO1, aliases TMEM16A/DOG1), a calcium-dependent chloride channel protein, locates in 11q13 [8], which is commonly amplified in several cancers, including the breast [9,10], the stomach [11,12], the ovary [13], and the prostate [14,15], glioblastoma [16], and head and neck cancer [10,17,18,19,20]. ANO1 is an established biomarker for gastrointestinal stromal tumors (GIST) [12]. ANO1 expression is associated with cell proliferation [10,12,13,14,19,20,21,22,23], migration [11,17,18,22], invasion [11,13,14,17], metastasis [14,17,20], tumor growth [10,13,14,19] and survival [11,18]. ANO1 interacts with EGFR [24], participates in its downstream signaling [10,19] and associates with scaffolding/signaling proteins, including ezrin, radixin and moesin [25]. ANO1 is also linked to tumorigenesis-promoting MAPK [19], as well as apoptosis-evasive TNF-α [15] signaling. A comprehensive mechanistic understanding between ANO1 expression and HNSCC tumorigenesis is still lacking, but the frequency of its overexpression in HNSCC [26] suggests that ANO1 might be a potent target for pharmacological intervention. Several small molecules targeting ANO1 have been developed [9,27,28], although a newer class of inhibitors, Ani9 [29,30], warrants further evaluation.

Myeloid cell leukemia-1 gene (*MCL-1*), encodes for MCL1, a BCL2 family member with crucial role in the regulation of apoptosis and cell cycle progression [31]. Cancer cells, including HNSCC, may hijack these processes by overexpressing MCL1, and thus promote cell survival [32,33]. Besides sharing many functional and structural similarities with other BCL2-family pro-survival proteins, MCL1 exhibits unique BH3-binding properties, a short protein half-life [34], and the ability to regulate mitochondrial metabolism [35]. Therefore, MCL1 presents a potential drug target for small molecule BH3-mimetic molecules that favor the induction of apoptosis [36,37], some of which are currently under investigation with promising outcomes [38].

p27^Kip1^, encoded by *CDKN1B* gene, is a cyclin-dependent kinase inhibitor that regulates G1-S phase transition by binding to cyclin E-CDK2 [39]. During early G1, p27^Kip1^ promotes the assembly and nuclear translocation of cyclin D1-Cdk complexes [39]. Mitogenic signals promote the assembly of cyclin D-CDK4 complexes that sequester p27^Kip1^ restraining its activity and favor the timely activation of cyclin E-CDK2 complexes and progression of the cell cycle [40]. Diverse mechanisms modulate p27^Kip1^ function at the level of its transcription, translation, post-translation, as well as subcellular localization [41]. Oncogene-activated pathways are involved in p27^Kip1^ nuclear export or cytoplasmic retention in human malignancies of epithelial origin, facilitating aberrant functions of p27^Kip1^, such as enhanced cell motility and migration [41].

In this study, we investigated the gene expression patterns involved in ANO1 functions and explored their molecular mechanisms in patient-derived HNSCC cell lines cultured in three-dimensional collagen matrix. We also studied the potential of ANO1 as a drug target in HNSCC cell lines with high endogenous ANO1 expression. Our data demonstrate that ANO1 expression facilitates the expression of MCL1 and regulates the expression, subcellular distribution and proteolytic degradation of p27^Kip1^, which may explain how ANO1 mediates cancer cell progression.

## 2. Results

### 2.1. ANO1 Expression Promotes Cell Proliferation and EMT of Primary Cancer Cells Derived from HNSCC

**Figure 1:**
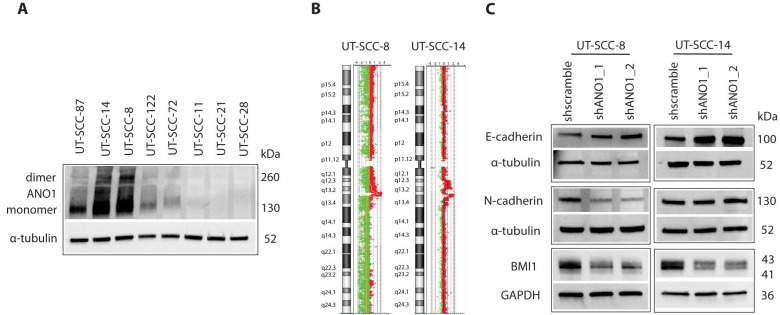
Comparison of ANO1 amplification and expression in a panel of University of Turku-Squamous Cell Carcinoma (UT-SCC) cell lines, and the effect of ANO1 expression on epithelial-to-mesenchymal transition (EMT). (**A**) Western Blot illustrating the expression of ANO1 in a panel of UT-SCC cell lines with diverse endogenous ANO1 expression levels. (**B**) Array comparative genomic hybridization (aCGH) profiles of chromosome 11 for UT-SCC-8 and UT-SCC-14 cell lines illustrating the high copy number amplification of 11q13. (**C**) Western blot analysis of EMT markers E-cadherin and N-cadherin, and stem cell marker BMI1 in shANO1 and shScramble cell lines. GAPDH and α-tubulin are the loading controls.

To study the function and involvement of ANO1 in HNSCC progression, we utilized cell lines from primary tumors of patients established at the University of Turku (UT-SCC cell lines). We have previously analyzed a mutational panel, copy number and gene expression profiles of 45 HPV-negative UT-SCC cell lines [42]. Among eight selected cell lines with diverse ANO1 mRNA expression (Figure S1A) [42], immunoblotting of ANO1 identified UT-SCC-8 and UT-SCC-14 as the cell lines with endogenously highest protein expression (ANO1^HIGH^, Figure 1A). We next verified the high level of amplification in 11q13 region, harboring ANO1 gene, by generating the copy number profiles of these cell lines (Figure 1B), based on array comparative genomic hybridization (aCGH) data [42].

Because silencing of ANO1 in cancer cells with 11q13 amplification results in reduction of cell proliferation, migration, and tumor growth [10,13,14,17,19], we knocked down ANO1 using two different shRNA constructs in ANO1^HIGH^ cell lines to study its role in HNSCC. In line with previous studies, ANO1 depletion in the ANO1^HIGH^ UT-SCC cells showed inhibition of cell proliferation (Figure S1B,C). Because ANO1 expression is linked to epithelial-to-mesenchymal transition (EMT) [11,20], we next examined if expression of selected EMT markers was affected by ANO1 depletion in the ANO1^HIGH^ UT-SCC cells. E-cadherin levels were increased (Figure 1C), suggesting that ANO1 depletion is sufficient to revert the phenotype to a less malignant one [43]. N-cadherin expression, an EMT marker that correlates with malignant behavior in HNSCC [44], decreased in ANO1 shRNA UT-SCC-8 cells. Of interest, a regulator of stemness, BMI1, was downregulated upon ANO1 depletion (Figure 1C). BMI1, together with Twist, can induce repression of E-cadherin expression in HNSCC [45], thus providing a possible explanation for E-cadherin increase in the ANO1-knockdown cells. These observations are in an agreement with previous findings that ANO1 promotes cell proliferation and EMT in HNSCC.

### 2.2. ANO1 Knockdown Leads to Downregulation of Pro-Survival Protein MCL1 Conferring Apoptosis

**Figure 2:**
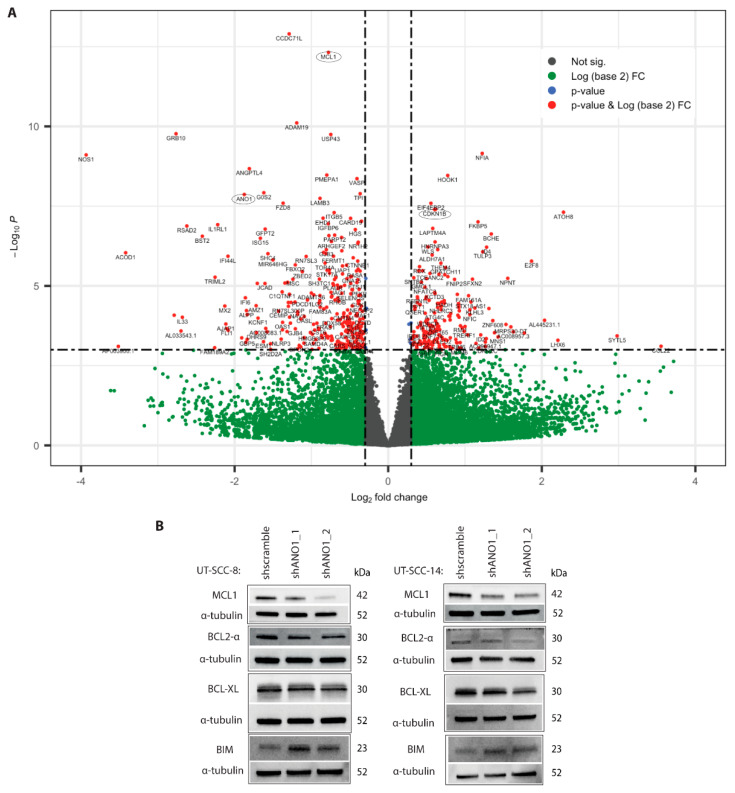
RNA sequencing revealed differential expression of genes involved in ANO1 signaling. (**A**) Volcano plot illustrating the differentially expressed genes. Each gene is represented by a dot. Red-colored genes pass the cutoffs of p_adj_, 10 × 10^−4^ and log2FC, 0.30 (above the horizontal dash line). (**B**) Western blots showing the differential protein expression between shScramble and shANO1 of key BCL2 family members, including pro-survival MCL1, BCL2, and BCL-XL, as well as pro-apoptotic BIM in UT-SCC-8 and UT-SCC-14 cell lines. Alpha-tubulin is the loading control.

To elucidate in an unbiased fashion the role of ANO1 in the cancerous properties of the ANO1^HIGH^ UT-SCC cell lines, we performed RNA sequencing from ANO1 silenced cell samples (shANO1) and their control counterparts (shScramble), cultured in three-dimensional collagen type I, which better recapitulates the tumor microenvironment [46]. Immunoblotting of ANO1 (Figure S2A) validated the efficiency of the knockdown prior to RNA sequencing. Differential expression analysis of ANO1 knockdown as compared to ANO1^HIGH^ UT-SCC cells (Figure S2B) revealed a total of 649 statistically significant altered genes, based on Benjamini-Hochberg adjusted p value (p_adj_ < 0.05, Figure 2A). Interestingly, MCL1 was one of the most significantly downregulated genes upon ANO1 knockdown (Table 1), based on the parameters log2FoldChange (log2FC) and p_adj_ and was visualized with a volcano plot (Figure 2A).

Downregulation of MCL1 mRNA levels upon ANO1 knockdown was concordant with a decrease at the protein level (Figure 2B), as well as with previous observations in breast cancer cell lines [10]. In addition, BCL-XL (alias BCL2L1) mRNA expression was downregulated (Table 1) and a modest downregulation was validated by immunoblotting (Figure 2B), supporting activation of the apoptotic cascade upon ANO1 knockdown. Furthermore, we examined the protein levels of pro-survival BCL2 and pro-apoptotic BIM (alias BCL2L11), which is known to interact with BCL2-protein members. Downregulation of BCL2 indicated the broad impact of ANO1 on pro-survival BCL-2 proteins. BIM was upregulated in the shANO1 samples (Figure 2B), which is in line with previous findings [47]. Apoptosis was measured by increased caspase activity in UT-SCC-8 shANO1 cells, using two different constructs (Figure S1D). Interestingly, FADD was also significantly downregulated (Table 1). FADD is closely located to ANO1 in the 11q13 locus and has been associated with proliferation and apoptosis of HNSCC [48].

### 2.3. ANO1 Exists in the Mitochondria of UT-SCC Cells with High ANO1 Expression

Because depletion of ANO1 modulates the expression of MCL1 protein, which is involved in apoptosis and functions in the mitochondria [35], we set to explore whether ANO1 protein is located in the mitochondria in ANO1^HIGH^ UT-SCC cells. We isolated the mitochondria-enriched and cytosolic fraction of the cell lysates and verified the presence of ANO1 in the concentrated mitochondrial fraction of the UT-SCC-8 cells (Figure S3A). COXIV was used as the mitochondrial marker. This is in agreement with studies utilizing pulmonary endothelial cells, in which ANO1 was present in the mitochondrial fraction [23,49]. We next performed immunoprecipitation experiment using a monoclonal ANO1 antibody and found that mitochondrial protein COXIV interacts with ANO1 (Figure S3B). Interestingly, the RNA sequencing analysis revealed the differential expression of several genes associated to dysfunctional mitochondria, including *PINK1*, *NDUFA13*, *PSEN2*, *FIS1*, *FURIN* and *COX7C* (Table 1).

To explore whether endogenous ANO1 expression correlates with the levels of selected proteins involved in mitochondrial apoptotic pathway, we studied by immunoblotting their expression patterns in five UT-SCC and two GIST cell lines with high ANO1 expression (UT-SCC-8, UT-SCC-14, UT-SCC-87, GIST48, GIST-T1), as well as two UT-SCC cell lines with low ANO1 expression (UT-SCC-11 and UT-SCC-95). MCL1 and BCL-XL were highly expressed in all the immunoblotted UT-SCC cell lines, supporting recent findings [33]. Interestingly, strong BCL2-b expression was observed in GIST cell lines (Figure S3C) but was almost undetectable in UT-SCC cell lines. In line with these findings, recent studies show dependency of HNSCC cells on both MCL1 and BCL-XL expression for survival, with little or no contribution from BCL2 [33,50,51].

### 2.4. Pathway Analysis Revealed ANO1 Involvement in Oncogenic Signaling, Including Apoptosis and Cell Cycle Progression

**Figure 3:**
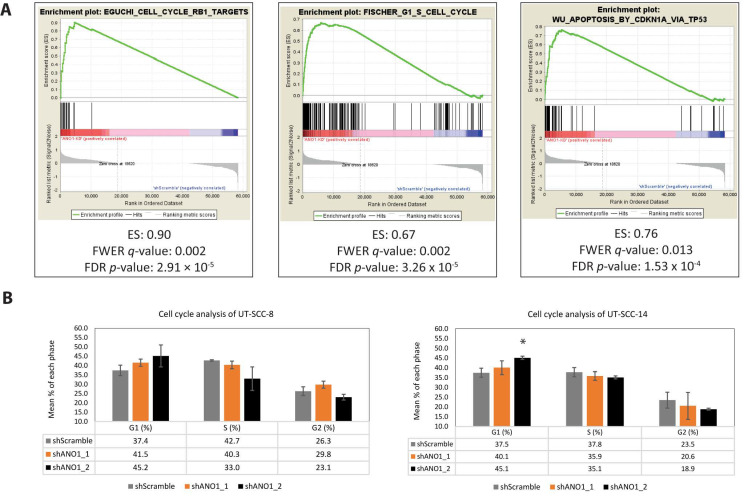
Gene Set Enrichment Analysis (GSEA) pathway analysis revealed ANO1 involvement in multiple cell cycle-related pathways. (**A**) Representative plots of significantly enriched pathways derived from the GSEA analysis. Criteria values for the selection were enrichment score (ES), family-wise error rate (FWER, *q*-value) < 0.025, and false discovery rate (FDR) < 0.05. (**B**) Bar plot graphs illustrating the mean percentage distribution of UT-SCC-8 and UT-SCC-14 shScramble, shANO1_1 and shANO1_2 cells in G1, S and G2 phases, respectively, *:*p*-value < 0.05.

The RNA sequencing dataset showed significant differential expression of genes established in the regulation of the cell cycle, including *CDKN1B*, *RB1*, *CDKN2C*, *E2F8* and *HDAC1* (Table 1). To unravel the exact signaling pathways of ANO1 involvement, we utilized this dataset to perform Gene Set Enrichment Analysis (GSEA), which revealed 59 significantly enriched pathways, based on the false discovery rate (FDR) and family-wise error rate (FWER) *p*-values (FDR < 0.05 and FWER < 0.025, Table S1). Interestingly, the majority of the pathways revealed a direct link of ANO1 to cell cycle progression-related functions, including ‘Chang cycling genes’ (enrichment score (ES) = 0.79, FDR = 0, FWER = 0), ‘Fischer G1 S Cell cycle’ (ES = 0.67, FDR = 3.26 × 10^−5^, FWER = 0.002 and ‘Whitfield Cell Cycle G1 S’ (ES = 0.67, FDR = 1.52 × 10^−4^, FWER = 0.012) (Figure 3A). Moreover, the GSEA analysis revealed pathways related to retinoblastoma (RB) or E2F-targets pathways, with roles in the regulation of transcription, DNA damage, and G1-S cell cycle progression. Additionally, the apoptotic pathway ‘Wu Apoptosis by CDKN1A via TP53’ (ES 0.76, FDR = 1.53 × 10^−4^, FWER = 0.013) was significantly enriched in the ANO1-depleted group, supporting the role of ANO1 expression in the evasion of apoptosis.

We next examined whether ANO1 depletion in the ANO1^HIGH^ cells affected cell cycle progression. In line with the GSEA findings, the proportion of ANO1-silenced cells in G1 phase of the cell cycle was increased, as compared to the control cells (Figure 3B). Cell cycle inhibition upon ANO1 depletion of ANO1^HIGH^ UT-SCC is in concordance with previous findings [22,52]. Interestingly, a key molecule in G2/M checkpoint regulation, the WEE1 kinase-encoding gene, was also significantly downregulated upon ANO1 depletion (Table 1). WEE1 is positioned downstream of MCL1 as a part of its non-apoptotic nuclear functions [34] and thus its downregulation may be responsible for complementary inhibition in the cell cycle progression.

### 2.5. ANO1 Regulates p27^Kip1^ Stability and Subcellular Localization

**Figure 4:**
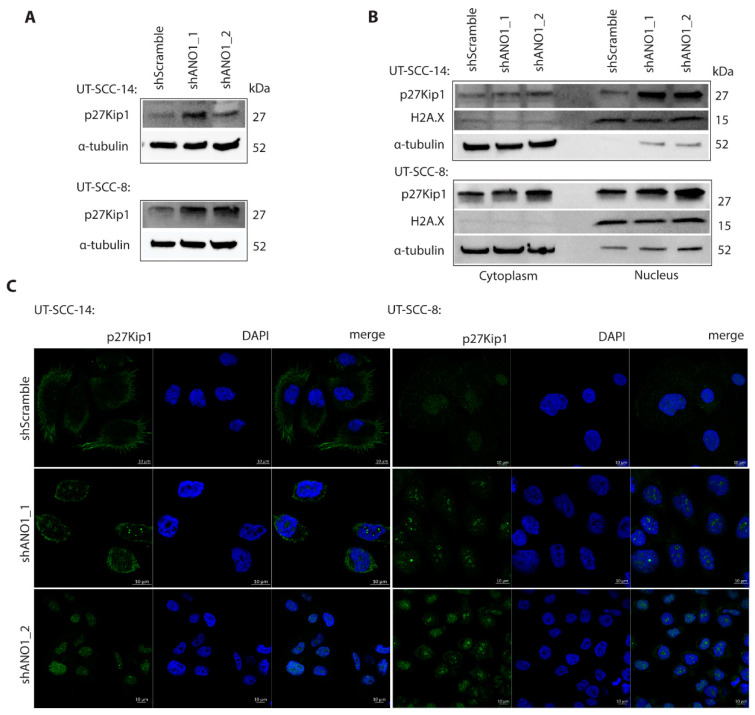
ANO1 modulates localization of p27^Kip1^ and expression of these genes is inversely correlated. (**A**) Expression of p27^Kip1^ in UT-SCC-14 and UT-SCC-8 shScramble and shANO1 cell lines, respectively, by immunoblotting. Alpha-tubulin is the loading control. (**B**) Western blot analysis depicting the distribution of p27^Kip1^ expression following fractionation of nuclear and cytoplasmic lysate extracts. H2A.x is the marker of nuclear fraction and α-tubulin marker of the cytoplasmic fraction in UT-SCC-14 and UT-SCC-8 shScramble and shANO1 cell lines. (**C**) Representative immunofluorescent confocal microscopy images depict the differential intensity and subcellular localization of p27^Kip1^ in UT-SCC-14 and UT-SCC-8 shScramble and shANO1 cell lines, respectively. Scale bars included in the right corner of each image indicate the respective size.

Having shown that ANO1 depletion results in halt of the cell cycle progression (Figure 3A,B), decreased proliferation (Figure S1B), and exacerbated apoptosis (Figure S1D), we set to examine the role of p27^Kip1^ in the context of ANO1 expression, due to its involvement in cell cycle progression, proliferation and cell motility [39,40,41]. In agreement with our RNA sequencing dataset, whole cell lysates of ANO1-depleted ANO1^HIGH^ cells showed increased expression of p27^Kip1^, compared to control cells (Figure 4A).

Post-translational modifications affecting protein stability and subcellular localization of p27^Kip1^ may interfere with its canonical function as cell cycle inhibitor and tumor suppressor [41]. We therefore isolated protein extracts from the nuclear and cytoplasmic fractions and found redistribution and differential p27^Kip1^ protein levels in shScramble compared to shANO1 samples of ANO1^HIGH^ cell lines (Figure 4B). We additionally examined by immunofluorescent staining whether differential expression of ANO1 affects p27^Kip1^ localization. Indeed, result of ANO1 silencing was the redistribution of p27^Kip1^ from modest expression at the cell edges and the cytoplasm to an intensified nuclear and perinuclear localization (Figure 4C). These findings highlight that ANO1 orchestrates the biological function of CDKN1B by regulating its protein levels and cellular localization in the studied cell lines. Interestingly, ANO1 depletion led also to downregulation of TRIM21 mRNA expression (Table 1). TRIM21 encodes for an E3 ubiquitin ligase that, when part of the SCF^SKP2^ complex, mediates proteolytic degradation of p27^Kip1^, thus promoting cell cycle progression [53]. In one ANO1-interactome study, TRIM21 has emerged as one of the association partners of ANO1 [25].

### 2.6. New ANO1 Small-Molecule Inhibitor Efficiently Decreases Cell Viability of ANO1^HIGH^ HNSCC Cell Lines

We showed that ANO1 expression is linked to proliferation and evasion of apoptosis in the ANO1^HIGH^ cells, indicating that ANO1 expression can potentially serve as a biomarker and drug target for the treatment of HNSCC. Ani9 is a newer class of small-molecule inhibitors with IC50~110 nM, as determined for endogenous human ANO1 in cellular assay and with highest selectivity for the ANO1 member of the anoctamin family of channel proteins [29]. We therefore examined the pharmacological potential of Ani9 and its three-fold more potent derivative, Ani9-5f [30], in ANO1^HIGH^ cell lines. As anticipated, Ani9-5f was more potent inhibitor as compared to its predecessor Ani9, which resulted in mild decrease of cancer cell viability and reduction of ANO1 protein levels (Figure S4A,B).

**Figure 5:**
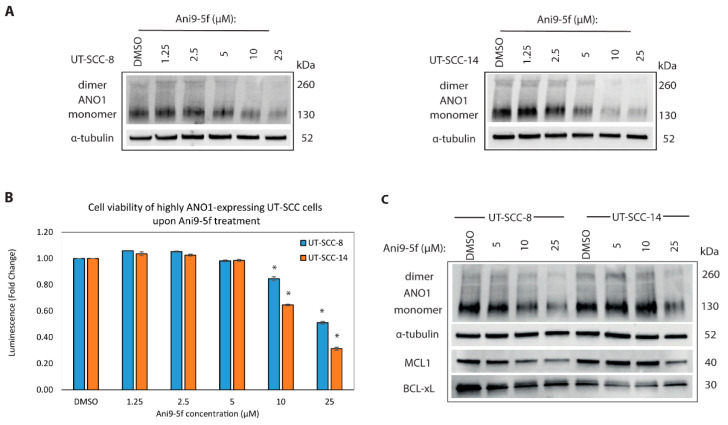
Pharmacological inhibition of ANO1 decreases cell viability and suppresses protein levels of ANO1, MCL1, and BCL-XL. (**A**) Western blots showing the decreasing ANO1 protein levels in UT-SCC cells, upon treatment with Ani9-5f for 48 h in a concentration-dependent manner. Alpha-tubulin is the loading control. (**B**) Bar plot illustrating the response of ANO1^HIGH^ UT-SCC cell lines to Ani9-5f small-molecule inhibitor, as measured by cell viability (Cell Titer Glow^®^), * *p*-value < 0.05. (**C**) Western blot panels indicate the differential protein levels of ANO1, MCL1 and BCL-XL upon treatment of UT-SCC-8 and UT-SCC-14 with the indicated concentrations of Ani9-5f. Alpha-tubulin is the loading control.

Previously proposed Ani9-5f mechanism of action suggests ANO1 degradation upon the selective binding and inhibition of ANO1 by Ani9-5f [30]. Ani9-5f treatment decreased ANO1 protein levels starting at 5 µM tested concentration (Figure 5A) and resulted in ~30% reduction in viability at 10 µM concentration (Figure 5B). To test the specificity of Ani9-5f, we measured changes of cell viability in shScramble compared to shANO1 of ANO1^HIGH^ cells post-treatment and found that cells with higher ANO1 expression were more sensitive to Ani9-5f-inhibition, reaching statistically significant difference in UT-SCC-14 cell line (Figure S5A,B).

Since ANO1 is an established biomarker in GIST, we tested Ani9-5f efficacy in reduction of cell viability in GIST-48 and GIST-T1 cell lines (Figure S6A,B). Predictably, Ani9-5f decreased cancer cell viability at higher concentrations (25–50 µM), due to the more pronounced expression levels of ANO1 in GIST, compared to ANO1^HIGH^ UT-SCC cell lines (Figure S3C).

Since Ani9-5f inhibition decreased ANO1 protein levels (Figure 5A), we examined whether it had an impact on the protein levels of the anti-apoptotic MCL1 similarly to shANO1-mediated silencing (Figure 2B). In line with the ANO1 silencing experiment, MCL1 protein levels decreased upon Ani9-5f treatment starting from 10 µM concentrations (Figure 5C), confirming the interplay between ANO1 and MCL1 expression.

### 2.7. Targeting MCL1 Induces Apoptosis with Concomitant Reduced Expression of BCL-XL

**Figure 6:**
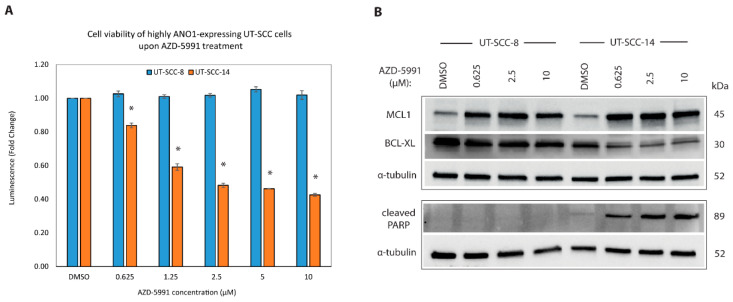
Targeted inhibition of MCL1 decreases cell viability and induces apoptosis in the selected ANO1^HIGH^ cell line. (**A**) Bar plot illustrating the efficacy of BH3-mimetic AZD-5991, as measured by cell viability (Cell Titer Glow^®^) in ANO1^HIGH^ cell lines, * *p*-value < 0.05. (**B**) Western blot panels showing the protein levels of MCL1, BCL-XL, and cleaved-PARP in UT-SCC-8 and UT-SCC-14, upon treatment with the indicated concentrations of AZD-5991.

We demonstrated that ANO1-5f resulted in reduction of cancer cell viability and ANO1 protein levels in ANO1^HIGH^ cells, as well as decrease in the expression of MCL1. Because the small-molecule inhibitors targeting ANO1 have been to date evaluated only in vitro, we investigated whether MCL1-mediated decrease of cancer cell viability is achievable by utilization of MCL1 inhibitor that is under clinical investigation. BH3-mimetics are small-molecule drugs, which interfere with the BH3-binding groove of anti-apoptotic proteins [37]. AZD-5991 is a BH3-antagonist, highly selective for MCL1 [38] and under investigation in Phase I/II clinical trials for hematologic malignancies (ClinicalTrials.gov Identifier NCT03218683). AZD-5991 acts by disrupting the MCL1-BAK complex leading to MCL1 stabilization and subsequent activation of BAK-dependent mitochondrial apoptotic pathway [38]. We treated ANO1^HIGH^ cell lines with a range of concentrations (0.625, 1.25, 2.5, 5 and 10 µM) of AZD-5991. We observed that UT-SCC-14 is a highly responsive cell line (IC50 = 2.5 µM), whereas UT-SCC-8 was resistant independently of the concentration (Figure 6A).

Resistance to BH3-mimetics can emerge by a shift in the balance of BCL2 family members [54]. Displacement of MCL1 interacting partners upon inhibition by AZD-5991 allows subsequent sequestration of the interacting partners by, i.e., BCL-XL. Increasing evidence from solid tumors suggests that several cancer cell types are dependent on more than one BCL2 family protein for survival and in particular, dual inhibition of MCL1 and BCL-XL is required to successfully facilitate apoptosis [33,50,51,54]. Endogenous BCL-XL protein expression was elevated in UT-SCC-8 compared to UT-SCC-14 and, increased among the AZD-5991 treated samples in a concentration-dependent manner, based on the densitometry measurements (Figure S7). In line with previous findings [38], AZD-5991 activity is blocked by overexpression of BCL-XL, providing a possible explanation for UT-SCC-8 resistance (Figure 6B). Increased cleaved-PARP confirmed the establishment of intrinsic apoptosis in UT-SCC-14 treated cells (Figure 6B).

## 3. Discussion

Copy number amplifications are frequently observed in the chromosomal region 11q13, which harbors besides *ANO1*, genes *CCND1*, *ORAOV1*, *PPFIA1*, *FADD* and *CTTN*, the expression of which is associated with cancer proliferation, migration, apoptosis or poorer prognosis [8,48]. In this study, we investigated the molecular role of ANO1 in oncogenesis of ANO1^HIGH^ HNSCC cell lines and explored novel transcriptional programs that are dependent on ANO1 expression.

Most of the existing research provide complementary evidence on the contribution of ANO1 expression to cancer cell proliferative properties [10,14,19,21]. Few studies, however, correlated the increased ANO1 expression with reduced proliferation and cell cycle arrest [17,18], implying that the role of ANO1 in cell proliferation may be cell-type dependent. In our studied cell lines, ANO1 silencing led to significant decrease in cellular proliferation. The expression status of EMT and stem cell markers changed upon depletion of ANO1 expression in ANO1^HIGH^ cells, which is in concordance with previous observations [11,44,45,52]. Acquainted mesenchymal properties in the case of UT-SCC-14 due to its aggressiveness and higher TNM stage compared to the UT-SCC-8, may explain the invariable N-cadherin levels. Interestingly, the mRNA of transforming growth factor alpha (*TGFA*), an EGFR ligand known to stimulate invasiveness in HNSCC [55] was significantly downregulated in our dataset.

RNA sequencing analysis of ANO1^HIGH^ cells and their knockdowns revealed significant differential expression of important cell cycle regulator genes, including *CDKN1B*, *RB1*, *CDKN2C*, *E2F8* and *HDAC1*. In this study, we investigated the role of p27^Kip1^, with canonical function in the regulation of cell cycle progression, mediated by its interaction with cyclin E-CDK2 [39] and cyclin D-CDK4 complexes [40]. We showed significant increase in the p27^Kip1^ mRNA and protein levels upon ANO1 knockdown. Additionally, we observed redistribution of p27^Kip1^ from a cytoplasmic to a mainly nuclear localization, emphasizing the role of ANO1 expression in p27^Kip1^ cytoplasmic sequestration and protein stability in HNSCC cells. Post-translational modifications and differential subcellular localization modulate primarily p27^Kip1^ function, acting either as tumor suppressor (nuclear) or as oncoprotein (cytoplasmic) [41,56,57]. Ubiquitination-mediated proteolysis is an independent mechanism of modulating p27^Kip1^; in the cytoplasm by the KPC complex [57] and in the nucleus by the SCF^SKP2^ [56]. We demonstrated for the first time that ANO1 modulates p27^Kip1^ stoichiometry in HNSCC cells at a diverse level, including transcription and translation, as well as by modification of its subcellular localization.

GSEA analysis showed enrichment of pathways associated with perturbed cell cycle progression, DNA damage/repair, as well as apoptosis in the ANO1-knocked down dataset. Cell cycle analysis supported the trend that higher expression of ANO1 is associated with accelerated pace of the cell cycle [21,22]. Interestingly, the activity of TRIM21 (alias Ro52)-containing SCF^SKP2^-like complex induces p27^Kip1^ ubiquitination and its degradation, ultimately accelerating cell cycle transition to S-phase [53]. The suppression of TRIM21 expression in ANO1-depleted cells co-occurred with a pronounced nuclear p27^Kip1^ localization and a G1-phase restricted phenotype. We propose that the higher prevalence of TRIM21 in ANO1^HIGH^ UT-SCC may be, at least partially, responsible for the increased degradation of p27^Kip1^. ANO1 downregulation, on the other hand, allows p27^Kip1^ accumulation to occur in the absence of TRIM21, thereby causing a p27^Kip1^-dependent G1 arrest in a similar manner as previously observed in the case of HeLa cancer cells [53]. Additionally, WEE1 mRNA, which is involved downstream of the non-apoptotic, nuclear MCL1 signaling pathway [34], was also downregulated in our dataset and may play a role in the complementary inhibition of cell cycle progression. WEE1 is a potential therapeutic drug target for HNSCC [58].

In addition to controlling proliferation and cell cycle progression, ANO1 expression is associated with resistance of apoptotic cell death in several tumor types [10,15,22,47]. The mechanism, however, of ANO1 contribution to the evasion of apoptosis has remained unclear and the proposed explanations are cell-type dependent. BCL2-family protein members are important regulators of the cancer cell fate, particularly in the context of anti-cancer targeted therapies [37]. Suppression of the pro-survival proteins, including MCL1 and BCL-XL, or upregulation of pro-apoptotic proteins, such as BIM, result in the activation of BAX/BAK and permeabilization of the outer mitochondrial membrane, ultimately leading to apoptotic cell death [36]. The balance between anti- and pro-apoptotic mediators is often more crucial for the outcome of cell survival, compared to individual protein’s expression levels [36].

The RNA sequencing results highlighted MCL1 as one of the most significantly differentially expressed genes. This result prompted us to investigate the role of BCL2-family members in ANO1-mediated transcriptional regulation. Besides MCL1 protein reduction, we showed that ANO1 depletion led to downregulation of BCL-XL and BCL2, as well as upregulation of BIM at the protein level, corroborating findings in other cellular models [10,47]. We demonstrated that ANO1 is present in the enriched mitochondrial fraction of ANO1^HIGH^ cell lines, which is in line with a study utilizing pulmonary epithelial cells [23], and showed ANO1 interaction with the mitochondrial marker COXIV by co-immunoprecipitation experiment.

MCL1 functions are regulated by interaction with its partners, post-translational modifications, or differential subcellular localization [34]. Outer mitochondrial membrane (OMM) is associated with MCL1 function in apoptosis [35], nuclear localization is involved in the regulation of the cell cycle [31,59], whereas mitochondrial matrix localization is linked to the mitochondrial processes [35]. Interestingly, genes associated with mitochondrial dysfunction were differentially expressed upon depletion of ANO1 expression, including *PINK1*, *NDUFA13*, *PSEN2*, *FIS1*, *FURIN*, *COX7C*. Specifically, mRNA expression of PINK1 and OMM protein, FIS1, were downregulated. Loss of MCL1 has been previously associated with mitochondrial dysfunction, impaired autophagy and defective PINK1-PARK2 signaling, due to decreased mitochondrial PINK1 protein levels in adult myocytes [60]. On the other hand, FIS1 is an important regulator of mitochondrial dynamics and influences the assembly of mitochondrial fission complexes towards the execution of mitochondrial fragmentation [61]. The potent effect of ANO1 expression in MCL1-mediated mitophagy, mitochondrial morphology and fission in UT-SCC ANO1^HIGH^ cell lines requires additional studies.

Several ANO1 inhibiting compounds have been under investigation [27,28,29]. In this study, we explored ANO1 as a potential target of pharmacological inhibition, as well as a biomarker of response to targeted therapy. Newly developed Ani9-based inhibitor [30] induced significant reduction of ANO1^HIGH^ HNSCC cells, resulting in similar reduction of MCL1 protein levels. We compared Ani9-5f-mediated effect on cell viability between shScramble and shANO1 cells and found that highly expressing cells were more sensitive to the compound. Further assessment of Ani9-5f potential in vivo is reasonable, since effective targeted-therapy options for patients with advanced HNSCC are lacking. Since MCL1 emerged as a key mediator of ANO1^HIGH^ cancer cell signaling and survival, we evaluated the potency of a specific MCL1 small-molecule inhibitor currently in clinical trials [38]. One cell line was resistant to MCL1-targeted treatment, irrespective of the concentration used. In this case, the upregulation of BCL-XL may confer a compensatory adaptation mechanism, providing possible explanation for the resistance [33,50,51,54]. BH3 profiling of the HNSCC cell lines and targeted treatment experiments using a BCL-XL-mimetic will perhaps further elucidate the mechanism of resistance to AZD-5991. A follow-up study of BCL-XL and MCL1 dual inhibition would assess whether the sensitization of the resistant ANO1^HIGH^ HNSCC cell line is dependent on multiple anti-apoptotic proteins for survival [33].

## 4. Materials and Methods

### 4.1. Cell Culture

The UT-SCC cell lines were established from primary HNSCC tumors at the Department of Otorhinolaryngology-Head and Neck Surgery, Turku University Hospital (Turku, Finland) by Prof. Reidar Grènman [62]. We selected a panel of HPV-negative UT-SCC, including cell lines with high and low ANO1 expression levels [42]. UT-SCC-8 (T_2_N_0_M_0_ and G1 grade) and UT-SCC-14 (T_3_N_1_M_0_ and G2 grade), derived respectively from the larynx and tongue, were selected for further studies due to their endogenously high ANO1 protein expression, and to which we refer here as ANO1^HIGH^ cell lines. Genomic DNA was purified using NucleoSpin^®^ Tissue Kit (Macherey-Nagel, Düren, Germany) for the genotyping of UT-SCC-8 and UT-SCC-14 cell lines (FIMM, University of Helsinki), according to manufacturer’s instructions. All used UT-SCC cell lines were negative for mycoplasma using a mycoplasma PCR Detection Kit [42] and MycoAlert^TM^ Detection Kit (Lonza, Basel, Switzerland). UT-SCC cells were cultured in DMEM (Lonza), supplemented with L-glutamine (2 mmol/L; Lonza), FBS (10%; Thermo Fisher Scientific, Waltham, MA, USA), penicillin/streptomycin (100 U/mL; Lonza), and non-essential amino acids (NEAA, 0.1 mM; Lonza). UT-SCC-14 was cultured in DMEM:F12 (Lonza), supplemented with FBS (10%; Thermo Fisher Scientific), penicillin/streptomycin (100 U/mL; Lonza), NaBicarbonate (0.1%; Lonza), and NaPyruvate (0.5 mmol/L/L; Lonza). Cells were detached by using Trypsin EDTA (200 mg/L, Lonza). Cells were maintained at a steady atmosphere of 37 °C and 5% CO_2_. GIST-48 and GIST-T1 were cultured in RPMI-1640 medium (Thermo Fisher Scientific), supplemented with penicillin/streptomycin (100 U/mL; Lonza), FBS (20%; Thermo Fisher Scientific), and L-glutamine (2 mmol/L; Lonza).

### 4.2. Antibodies, Reagents and Chemicals

The following monoclonal antibodies used were purchased from Cell Signaling Technologies (CST, Danvers, MA, USA): Rabbit ANO1 (D1M9Q, 14476), rabbit MCL1 (5453 or 4572); rabbit BCL2 (4223), rabbit BCL-XL (2764), rabbit BIM (2933), rabbit N-cadherin (13116S), rabbit E-cadherin (3195S), rabbit BMI1 (6464S), rabbit cleaved PARP (Asp214, 5625). The following monoclonal antibodies were also used: Rabbit IgG (Alpha Diagnostic International, San Antonio, TX, USA) for the co-immunoprecipitation, and rabbit p27^Kip1^ (32034, Abcam, Cambridge, UK) for both immunofluorescence staining and western blotting. Monoclonal mouse α-tubulin (clone DM1A, T6199, Sigma-Aldrich, St. Luis, MO, USA) was used either as loading control or as marker of the cytoplasmic fraction. Mouse GAPDH (97166, CST) were used as loading control. Monoclonal rabbit histone H2A.X (7631, CST) was used as marker of the nuclear fraction. Mouse COXIV (33985, Abcam) was used as a mitochondrial marker. For Western blotting, secondary antibodies horseradish peroxidase (HRP) conjugate goat anti-mouse IgG and HRP conjugate goat anti-rabbit IgG (Life Technologies, Carlsbad, CA, USA) were used for the detection of mouse and rabbit primary antibodies, respectively. For immunofluorescence microscopy, as secondary antibody Alexa Fluor 488 of goat anti-rabbit IgG (Life Technologies) was used. Rat-tail collagen type I was purchased from Sigma-Aldrich (C7661). Small-molecule inhibitor, Ani9, was purchased from TOCRIS (6076, Bristol, UK). Prof. Wan Namkung (Yonsei University) kindly provided the Ani9-5f inhibitor. AZD-5991 was purchased from Active Biochem. Ltd. (A-6097, Hong Kong, China).

### 4.3. Generation of the ArrayCGH Profiles

The array comparative genomic hybridization (aCGH) data are available under GEO accession number GSE108062 [42]. The copy number profiles were generated using Genomic Workbench software 7.0 (Agilent) and the ADM-2 algorithm. The continuous line shows moving average of log2 copy number ratios with 2 Mb window.

### 4.4. Generation of Stable ANO1-Knockdown UT-SCC Cells

The shRNA constructs were derived from the TRC1 library (Sigma-Aldrich) and target specifically human TMEM16A (alias ANO1) mRNA. shRNA constructs were cloned into the pLKO.1 vector with the puromycin resistance gene. Lentiviral particles harboring the shRNA constructs for ANO1 knockdown (clone IDs: TRCN0000040265 and TRCN0000040266) and non-targeting controls (clone ID: SHC002) were generated at the Biomedicum Functional Genomics Unit (FuGU Libraries, University of Helsinki). For the transduction of cells with each construct, 50,000–80,000 cells/mL were seeded into 12-well plates and were let to adhere overnight. The following day, supernatant was replaced with fresh media. Selection of the transduced cells was performed by addition of 1 μg/mL puromycin (Sigma-Aldrich) in the media.

### 4.5. Sample Preparation for RNA Sequencing

Cells were cultured as single-cell suspension in three-dimensional collagen type I, since it resembles closely the tumor’s adjacent extracellular matrix and enables the study of migratory and invasive properties in HNSCC [46]. Shortly, collagen type I (A1048301, Thermo Fisher Scientific) was diluted according to manufacturer’s instructions in 10 × PBS (Lonza), 1M NaOH and sterile milliQ water. The solution was kept on ice. Stably transduced UT-SCC-8 and UT-SCC-14 shScramble, as well as shANO1 cell lines were counted and pipetted with the collagen suspension at a final concentration of 300,000 cells/mL and plated to poly (2-hydroxyethyl methacrylate) (poly-HEMA; Sigma-Aldrich) pre-coated 24-well plates. Supplemented media was carefully exchanged daily. Each stably transduced cell line, including appropriate number of technical replicates, was incubated in collagen suspension, and was collected on day five upon formation of packed, cobblestone colonies, as visualized by light microscopy. Three technical replicates from each biological sample were further processed for RNA sequencing.

### 4.6. RNA Extraction and Purification

Trizol (Life Technologies) was added to each sample cultured as inert in collagen. Each sample was dissociated from its matrix solution using the Precellys beads (Bertin instruments, Montigny-le-Bretonneux, France). Chloroform was added and samples were shaken thoroughly. A clear phase was formulated during 2 min incubation time at room temperature (RT), following sample centrifugation at 11,300 rpm for 15 min at +4 °C. The transparent phase was transferred to a new tube, followed by addition of isopropanol and mixture of solution. Samples were kept at RT for 10 min and centrifuged at +4 °C, 11,300 rpm, followed by addition of 75% EtOH. RNA was detected as white pellet. The samples were centrifuged at +4 °C, 8800 rpm for 5 min. Supernatant was discarded and pellets were dissolved in RNAse free water at +55 °C for 10 min. Total RNA was purified further using RNeasy Mini Kit (Qiagen, Hilden, Germany), according to manufacturer’s instructions (Qiagen). The RNA quality was determined using TapeStation 4200 (Agilent Technologies, Santa Clara, CA, USA). Samples with RIN values above 9.0 were further processed.

### 4.7. RNA Sequencing and Data Analysis

RiboZero Complete Gold Human kit (Illumina, San Diego, CA, USA) was used to remove ribosomal RNA from the total RNA (1.5 µg). Shortly, the ribosomal depleted RNA was purified with RNeasy mini Elute columns (Qiagen), and the absence of rRNA and the quantity of mRNA was measured with Bioanalyzer. Libraries were prepared using NEBNext Ultra Directional RNA library prep kit (New England Biolabs, Ipswich, MA, USA), according to manufacturer’s instructions at the Biomedicum Functional Genomics Unit (FuGU, University of Helsinki). The amplified library was then purified using AMPure XP Beads (Beckman Coulter Life Sciences, IN, USA). Library quality was assessed with Bioanalyzer (DNA High Sensitivity chip, Agilent) and library quantity by Qubit (Invitrogen, Carlsbad, CA, USA). Libraries were sequenced with NextSeq500 system (High 75 bp, Illumina).

Base calling was performed with Real-Time Analysis v2 (NextSeq 500, Illumina). Fastq files were generated by using bcl2fastq (v2.20.0.422). Read quality was verified by FASTQC (v0.11.3). Trimmomatic (v0.36) was used for quality trimming and removal of any remaining adapter sequences from the data. High-quality reads were then used as an input for STAR (v2.5) to acquire genomic alignments. Reads were mapped to the Homo sapiens reference genome (build GRCh38) and annotated using gencode human release 28 genome and gtf-files. Uniquely mapped reads were assigned to genomic features using the Subread package (v1.5.1) and featureCounts function.

Differential expression analysis was performed by DESeq2 package in R environment (v3.5.1). The resulting *p*-values were adjusted for multiple testing using the Benjamini and Hochberg method of FDR (p_adj_) [63] and genes with value p_adj_ < 0.05 were considered significantly differentially expressed. The RNA seq results were functionally annotated using Ensembl [64]. The volcano plot was created in R environment (v4.0.3) using the EnhancedVolcano package (https://github.com/kevinblighe/EnhancedVolcano, access date 25 January 2019).

### 4.8. Gene Set Enrichment Analysis (GSEA)

Gene set enrichment pathway analysis [65] was performed to detect in which curated datasets from the Molecular Signature Database (MSigDB) the significantly differentially expressed genes from our dataset were enriched, when comparing control (shScramble) and ANO1 (shANO1) knockdown cells. C2 curated dataset (6226 gene sets) was utilized (www.gsea-msigdb.org, access date 2 January 2019). Parameters used were gene set size filters (min = 5, max = 2000) and the number of permutations (*n* = 1000). The enriched pathways were sorted in descending order of their normalized enrichment score (NES). Statistically significant pathways were considered those with FDR (*p*-value) below 0.05 and FWER (*q*-value) below 0.025.

### 4.9. Protein Extraction, Immunoblot and Immunoprecipitation

For the protein extraction of whole cell lysates, harvested cells were incubated on ice with RIPA buffer (Thermo Scientific), supplemented with protease and phosphatase inhibitor cocktail (Roche, Basel, Switzerland). The lysed cells were incubated into pre-chilled, sterile microcentrifuge tubes, rotating for 20 min at +4 °C, followed by centrifugation at 14,000× *g* for 15 min at +4 °C (Thermo Scientific). Protein concentration was determined by either DC protein assay kit (BioRad, Hercules, CA, USA) or Pierce BCA Protein Assay Kit (23225, Thermo Scientific), according to manufacturer’s instructions. Clarified lysates were suspended with 2× Laemmli buffer/beta-mercaptoethanol (BioRad) and boiled at 95 °C for 5 min.

Isolation of nuclear and cytoplasmic extracts was performed using the NE-PER Nuclear and Cytoplasmic Extraction Reagents (78833, Thermo Scientific), according to manufacturer’s instructions. Isolation of mitochondrial extracts was performed using mitochondria isolation kit for cultured cells (89874, Thermo Scientific) according to manufacturer’s instructions.

Interaction between ANO1 and COXIV was studied by co-immunoprecipitation. Protein G sepharose beads (193259, Abcam) were activated by washing in 3× volume of Lysis Buffer (2% CHAPS in HEPES) and spinned at 150× *g*. Cells were previously seeded in 15 cm culture dishes (Sigma-Aldrich) and lysed directly in ice cold 2% CHAPS in HEPES pre-cleared lysates, when 80% confluent. A pre-clearance step of the clarified lysates was performed by gentle agitation of lysates with beads for 10 min at 4 °C, followed by centrifugation. Pre-cleared lysates were incubated with 1 µg of ANO1 antibody and gently rotated at 4 °C for 1 h. The antibody-protein complex was further incubated with protein G sepharose beads for 2 h. After washing the beads three times with CHAPS buffer, the bead-bound proteins were eluted by boiling for 5 min at 95 °C in Laemmli/β-mercaptoethanol sample buffer. Eluted proteins were collected by spinning.

Denatured protein samples were run using precast mini gels TGX of gradient 4–20% (30 µL wells) or 4–15% (50 µL wells, BioRad) and transferred using either a mini or midi PVDF transfer pack (BioRad) and Trans-Blot^®^ Turbo™ Transfer System (BioRad). Membranes were incubated at RT in blocking solution consisting of 5% milk powder or 5% bovine serum albumin (BSA) in tris-buffered saline with Tween (TBST), followed by TBST washes. Membranes were incubated with the primary antibodies overnight at 4 °C with shaking. Membranes were then washed with TBST followed by incubation with secondary antibody for 1 h and washing with TBST. Proteins were detected using Chemidoc Imaging System (BioRad) and Immobilon Western Chemiluminescent HRP Substrate (Merck-Millipore, Burlington, MA, USA). Densitometry analysis was performed using ImageLab software (BioRad). The relative intensities were calculated as the ratio of the background-adjusted volume intensities of interest divided by their corresponding background-adjusted loading control intensities. The uncropped blots with densitometries and intensity ratios are presented in Figure S7.

### 4.10. Cell Proliferation Assay (MTT)

Cell proliferation was assessed with the 3-(4,5-dimethylthiazol-2-yl)-2,5- diphenyltetrazolium bromide (MTT, M6494; Sigma-Aldrich). Cells were seeded at a density of 5 × 10^3^ cells per well in 96-well plates in supplemented DMEM medium. After culturing for 6, 24, 48, and 72 h, respectively, 10 µL of MTT in PBS solution (5 mg/mL) was added to each well and cells were incubated at +37 °C for 2 h in the dark. One hundred microliters of lysis buffer (20% SDS, 10 mM HCl) were then added to each well and cells were incubated overnight. The absorbance of the MTT reduction product formazan was measured at 540 nm using VICTOR^TM^ plate reader (Perkin Elmer, Waltham, MA, USA).

### 4.11. Cell Viability, Apoptosis, and Drug Treatment Assays

UT-SCC and GIST cell lines were seeded on white, 96-well, view microplates with optically clear bottom (Perkin Elmer) at a concentration of 5–8 × 10^3^ cells/well. Cell concentration was optimized based on each cell line’s growth rate to avoid overconfluency during the experiment. Cells were incubated overnight with standard conditions to properly adhere at the bottom of the wells. Media was exchanged with either DMSO (control) or range of drug concentrations including technical replicates per each condition. Cells were treated for 48 h. Inhibitors used were Ani9, Ani9-5f, and AZD-5991. CellTiter-Glo^®^ Luminescent Cell Viability Assay to detect changes in cell viability (G7571, Promega, Madison, WI, USA), and Caspase-Glo^®^ 3/7 Assay to detect changes in apoptosis were used (G8093, Promega), following the manufacturer’s instructions. Luminescence was measured using VICTOR^TM^ plate reader (Perkin Elmer).

### 4.12. Cell Cycle Analysis

Each cell line was seeded so that at least 1 × 10^6^ cells were available on the harvest day. Cells were trypsinized and washed twice with 1 × PBS. The cell pellet was kept, and supernatant was discarded after each wash. Cells were fixed and permeabilized using 70% (vol/vol %) EtOH. After fixation, cells were kept at +4 °C for at least 4 h. Cells were then centrifuged at 500× *g* for 5 min, followed by a single wash with 2% FBS/PBS. Supernatant was carefully removed to not disintegrate the pellet. Cells were treated with 1 mg/mL RNase A and incubated at + 37 °C for 30 min. Cells were stained with 10 µg/mL propidium iodide (P3566; Thermo Scientific) and were incubated for at least 30 min at RT, protected from light. Samples were run in Biomedicum Flow Cytometry Unit (University of Helsinki) using flow cytometer Accuri C6 (Becton Dickinson, Franklin Lakes, NJ, USA) with flow up to 500 events/s and analyzed using FlowJo Software (Becton Dickinson). Results from three experiments are presented in Figure S8.

### 4.13. Immunofluorescence Staining and Confocal Microscopy

For immunofluorescence staining, cells were seeded on coverslips and were let to grow until 70–90% confluent. The cells were fixed using 4% parafolmaldehyde in phosphate-buffered saline (4% PFA/PBS), followed by two washes with PBS. Permeabilization and blocking of aldehyde groups was performed by 10 min incubation with 0.12% glycin, 0.1% Triton X-100/PBS, followed by PBS wash once. After blocking unspecific binding by incubating the cells for 60 min with 3% BSA/ PBS, the cells were washed once with PBS. Washing step was followed by incubation of cells with the primary antibody for 1 h at RT. Primary antibody was diluted 1:100 in 1% BSA/PBS. The cells were then washed with PBS followed by incubation in a secondary antibody dilution of 1:400 in 1% BSA/PBS for 1 h in dark at RT. The cells were washed three times with PBS, 5 min each, followed by two washes with MQ water, 5 min each. Cells were mounted in Mowiol containing 1.4-diazobicyclo (2,2,2) octane (DABCO) and 4′6-diamidino-2-phenylindole (DAPI) (Sigma-Aldrich) for nucleus staining.

Confocal images were taken using a Zeiss Meta 780 (Oberkochen, Germany) laser scanning microscope with a Zeiss 40× or a 63×/1.4 N.A. plan-apochromatic oil objective. Images were linearly adjusted, and channels combined in panels using ZenLite software (2.3 Lite; blue edition).

### 4.14. Statistical Analysis

In vitro experiments were repeated at least three times. Data were presented as fold changes (FC) of the average (mean) of the technical and experimental repeats ± the standard deviation of the mean (SEM), indicating the standard deviation between the measurements in the given set of data. Significant differences between the indicated pairs of data were ascertained using an unpaired student’s *t*-test and two-tailed distribution. The value of *p* < 0.05 was considered as statistically significant.

## 5. Conclusions

In summary, we showed for the first time that ANO1 expression modulates p27^Kip1^ stability and its subcellular distribution in the studied patient-derived HNSCC cell lines. High expression of ANO1 coincided with extensive cytoplasmic localization, a proliferative phenotype, and accelerated cell cycle progression. Conversely, ANO1 depletion in the studied ANO1^HIGH^ HNSCC cells resulted in the prompt nuclear relocation and protein accumulation of p27^Kip1^, thereby enhancing the execution of its canonical functions as cell cycle inhibitor. Several genes with role in protein ubiquitination pathways were significantly differentially expressed upon ANO1 depletion, such as TRIM21, which directly influences p27^kip1^ stability. Moreover, this study illustrated the downregulation of crucial pro-survival BCL2 family protein members, notably MCL1, by both lentiviral silencing and pharmacological inhibition of ANO1. Our findings highlight that ANO1 is an actionable target in HNSCC, as well as that novel ANO1-small molecule inhibitor, Ani9-5f, and BH3-mimetic, AZD-5991, are compounds with high clinical relevance for a subset of HPV-negative HNSCCs. Overall, we provided novel mechanistic insight on the involvement of ANO1 in oncogenic properties of tumors derived from the head and neck area, endorsing further investigation of ANO1 as a potential drug target in preclinical models.

## Figures and Tables

**Table 1:** List of selected significantly differentially expressed genes. The criteria for inclusion were p_adj_ < 0.05 from the RNA sequencing analysis and gene function associated with pathways inferred in the text and linked to ANO1 signaling, including mitochondrial and ubiquitination functions. Genes are listed in ascending order of p_adj_ value. Human Gene Nomenclature Committee (HGNC).

Gene Name	Log2FC	p_adj_	Description (Source HGNC)
*MCL1*	−0.78	4.79 × 10^−13^	Myeloid cell leukemia 1; BCL2 family member
*USP43*	−0.75	5.12 × 10^−7^	Ubiquitin specific peptidase 43
*CDKN1B*	+0.60	3.01 × 10^−5^	Cyclin dependent kinase inhibitor 1B
*TGFA*	−0.77	0.0001	Transforming growth factor alpha
*FIS1*	−0.40	0.0002	Fission, mitochondrial 1
*E2F8*	+1.87	0.0004	E2F transcription factor 8
*DNAJB4*	+0.74	0.0008	DnaJ heat shock protein family (Hsp40) member B4
*HLA-A*	−0.89	0.0029	Major histocompatibility complex, class I, A
*COX7C*	+0.70	0.0031	Cytochrome c oxidase subunit 7C
*PINK1*	−0.58	0.0066	PTEN induced putative kinase 1
*TRIM21*	−0.66	0.0077	Tripartite motif containing 21
*RB1*	+0.50	0.0077	RB transcriptional corepressor 1
*STUB1*	−0.33	0.0088	STIP1 homology and U-box containing protein 1
*PSMD5*	+0.27	0.0093	Proteasome 26S subunit, non-ATPase 5
*FADD*	−0.49	0.0102	Fas associated via death domain
*NDUFA13*	−0.40	0.0102	NADH: ubiquinone oxidoreductase subunit A13
*BCL-X_L_*	−0.54	0.0127	BCL2 like1; protein phosphatase 1, regulatory subunit 52
*WEE1*	+0.74	0.0188	WEE1 G2 checkpoint kinase
*PSMB10*	−0.63	0.0236	Proteasome subunit beta 10
*CDKN2C*	+1.27	0.0257	Cyclin dependent kinase inhibitor 2C
*FURIN*	−0.55	0.0266	Furin, paired basic amino acid cleaving enzyme
*PSEN2*	−0.50	0.0323	Presenilin 2
*HDAC1*	−0.36	0.0328	Histone deacetylase 1
*USP37*	+0.48	0.0334	Ubiquitin specific peptidase 37
*UBE2L6*	−1.16	0.0406	Ubiquitin conjugating enzyme E2 L6
*UBE2E3*	+0.34	0.0428	Ubiquitin conjugating enzyme E2 E3
*USP48*	+0.19	0.0481	Ubiquitin specific peptidase 48

## Data Availability

RNA sequencing data are deposited in Gene Expression Omnibus (GEO) publicly accessible repository with series accession number GSE163639. The data supporting the conclusions of this study are included within the article and the supplementary files.

## Supplementary Materials

The following are available online at https://www.mdpi.com/2072-6694/13/5/1170/s1, Figure S1. Knockdown of ANO1 inhibited proliferation and induced apoptosis in ANO1^HIGH^ UT-SCC cell lines. (A) Bar plot of the gene expression (mRNA) of ANO1 in selected UT-SCC cell lines, as previously determined by Illumina HT-12 human microarrays. The gene expression level is shown as previously reported log2 transformed average signal across UT-SCC cell lines [42]. (B) Differential cell proliferation in UT-SCC-8 and UT-SCC-14 shScramble compared to shANO1_1 and shANO_2 cell lines was assessed by MTT assay. Measurements were performed at the indicated timepoints (hours). Datapoints are shown as fold changes (FC) of the mean absorbances (540 nm). (C) Light microscopy images of UT-SCC-8 shScramble and shANO1 showing the morphological changes upon ANO1 silencing. (D) Caspase 3/7 activity in UT-SCC-8 cell line upon ANO1-knockdown with either shANO1_1 or shANO1_2 construct as compared to shScramble. Figure S2. (A) Western blot analysis showing ANO1 knockdown efficiency in UT-SCC cell lysates prior to RNA sequencing. (B) A heatmap visualizing the overall sample variations among UT-SCC-8 and UT-SCC-14, shANO1 and shScramble samples, respectively. Hierarchical clustering of the top 10,000 differentially expressed genes was performed based on the Euclidian distances of each gene and among each sample. Figure S3. (A) Western blot analysis of cytoplasmic and mitochondrial fractions of ANO1^HIGH^ cell line as well as shANO1_1 and shANO1_2 counterparts, depicting ANO1 localization in the concentrated mitochondrial fraction. COXIV is used as a mitochondrial marker and α-tubulin as a cytoplasmic marker of the respective cell fractions. (B) Western blots showing the co-immunoprecipitation of ANO1 with the mitochondrial marker, COXIV, in ANO1^HIGH^ UT-SCC cell lines. (C) Western blots illustrating the differences in the protein levels of the most significant pro-survival BCL2 family members in a panel of UT-SCC and GIST cell lines. Alpha-tubulin is the loading control. Figure S4. (A) Bar plot illustrating the potency of small molecule inhibitor Ani9 (precursor of Ani9-5f) in ANO1^HIGH^ UT-SCC cell lines as measured by cell viability (Cell Titer Glow^®^). Comparison is done between DMSO and each indicated concentration of Ani9 in UT-SCC-8 and UT-SCC-14. (B) Western Blots showing the decrease of ANO1 protein levels, upon treatment with Ani9 in a concentration-dependent manner. Figure S5. (A,B) Bar plot illustrating increased efficiency of UT-SCC-8 and UT-SCC-14 cells in the response to Ani9-5f targeted inhibition. Results are presented as comparison between DMSO and each indicated concentration of Ani9-5f in shScramble and shANO1_1 or shANO1_2 cells. Figure S6. (A) Bar plot illustrating the potency of small molecule inhibitor Ani9-5f in GIST-48 and GIST-T1 cell lines, as measured by cell viability (Cell Titer Glow^®^). Comparison is done between DMSO and each indicated concentration of Ani9-5f in GIST-48 and GIST-T1. (B) Western blots showing the degradation of ANO1 protein following treatment of the GIST-48 and GIST-T1 in the indicated inhibitor concentrations. Figure S7. Original Western blot images and densitometry measurements. The relative intensities were calculated as the ratio of the background-adjusted volume intensities of protein bands of interest divided by their corresponding background-adjusted loading control intensities. Figure S8. Three representative experiments of cell cycle analysis of UT-SCC-8 shScramble, shANO1_1 and shANO1_2, as well as UT-SCC-14 shScramble, shANO1_1 and shANO1_2, indicating the distribution percentage of cell cycle phases (G1, S and G2) in each condition. Table S1. Enriched gene sets in GSEA analyses after ANO1 knockdown in UT-SCC cancer cell lines cultured in 3D collagen I.
