# Supplementary material for: ANO1 Expression Orchestrates p27Kip1/MCL1-Mediated Signaling in Head and Neck Squamous Cell Carcinoma
Source: Cancers (Basel). 2021 Mar 9;13(5):1170. doi: 10.3390/cancers13051170 (PMC7967175; doi:10.3390/cancers13051170)
Supplement: Supplementary file 1 [file cancers-13-01170-s001.zip › cancers-1120165-supplementary-FINAL/Figures_S1-S8.pdf]

Figure S1

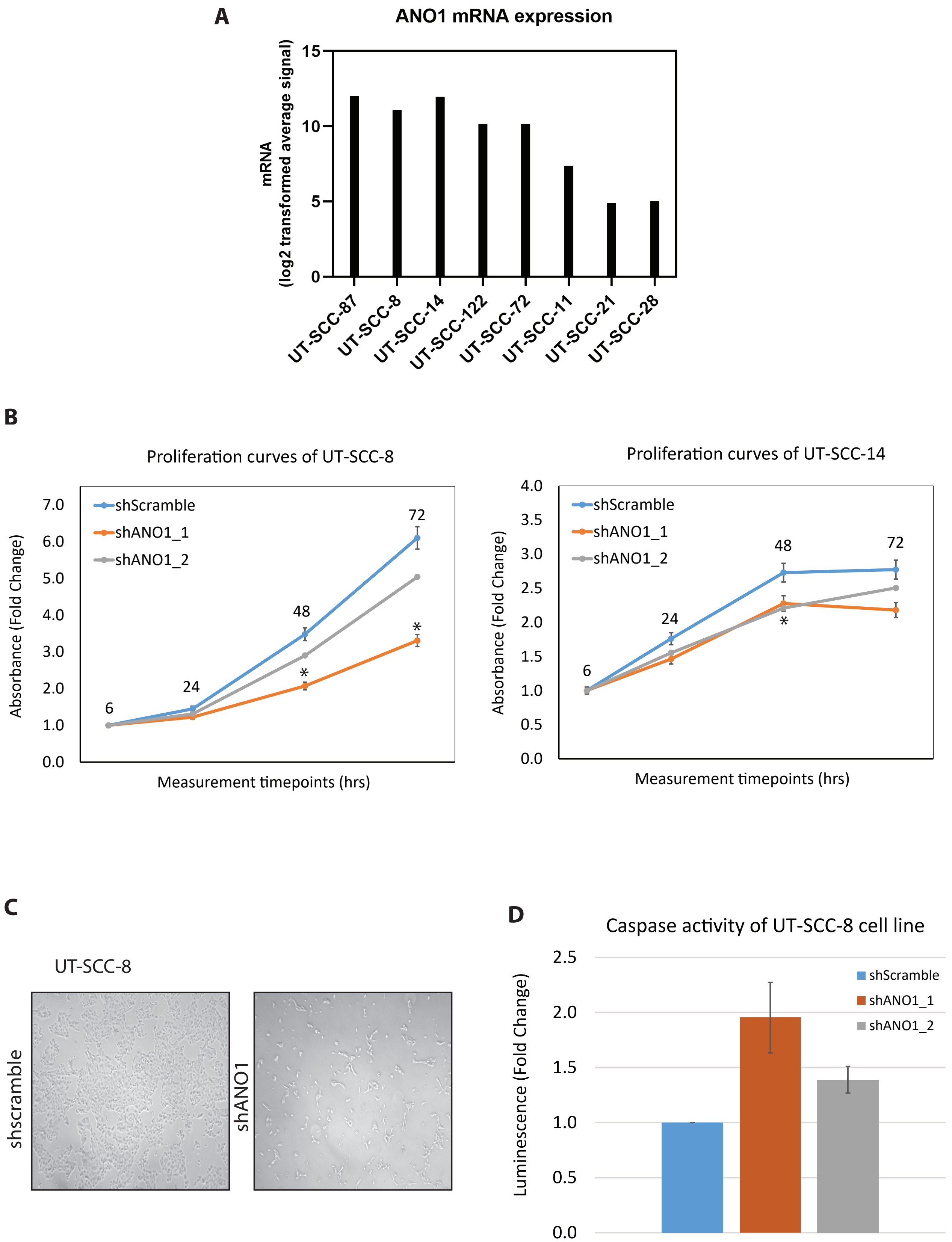
A
ANO1 mRNA expression
mRNA
(log2 transformed average signal)
15
10
5
0
UT-SCC-87
UT-SCC-8
UT-SCC-14
UT-SCC-122
UT-SCC-72
UT-SCC-11
UT-SCC-21
UT-SCC-28
B
Proliferation curves of UT-SCC-8
shScramble
shANO1_1
shANO1_2
Absorbance (Fold Change)
7.0
6.0
5.0
4.0
3.0
2.0
1.0
0.0
6
24
48
72
*
*
Measurement timepoints (hrs)
Proliferation curves of UT-SCC-14
shScramble
shANO1_1
shANO1_2
Absorbance (Fold Change)
4.0
3.5
3.0
2.5
2.0
1.5
1.0
0.5
0.0
6
24
48
72
*
Measurement timepoints (hrs)
C
UT-SCC-8
shscramble
shANO1
D
Caspase activity of UT-SCC-8 cell line
Luminescence (Fold Change)
2.5
2.0
1.5
1.0
0.5
0.0
shScramble
shANO1_1
shANO1_2

Figure S2

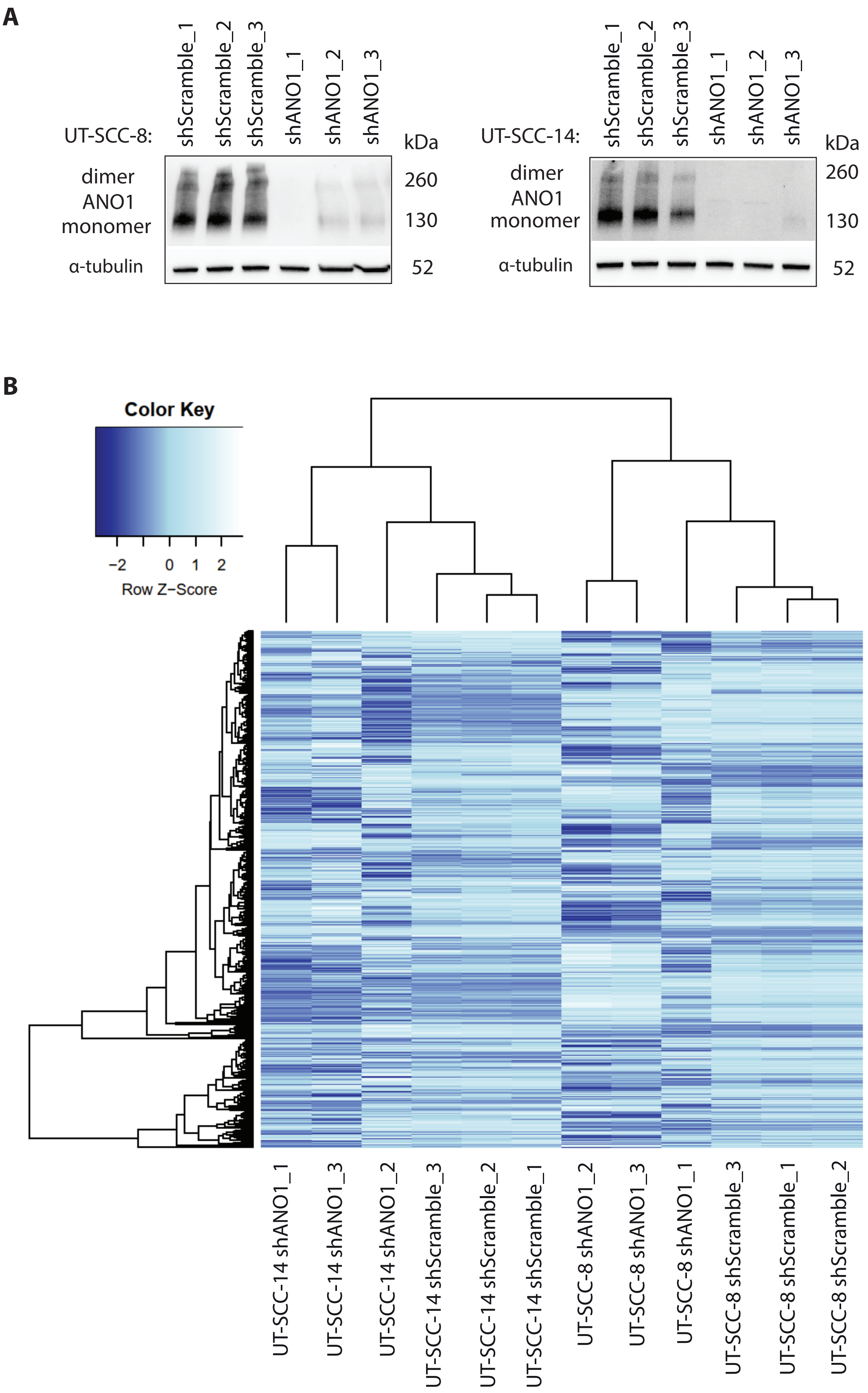
A
UT-SCC-8:
shScramble_1
shScramble_2
shScramble_3
shANO1_1
shANO1_2
shANO1_3
kDa
dimer
ANO1
monomer
α-tubulin
260
130
52
UT-SCC-14:
shScramble_1
shScramble_2
shScramble_3
shANO1_1
shANO1_2
shANO1_3
kDa
dimer
ANO1
monomer
α-tubulin
260
130
52
B
Color Key
−2
0
1
2
Row Z−Score
UT-SCC-14 shANO1_1
UT-SCC-14 shANO1_3
UT-SCC-14 shANO1_2
UT-SCC-14 shScramble_3
UT-SCC-14 shScramble_2
UT-SCC-14 shScramble_1
UT-SCC-8 shANO1_2
UT-SCC-8 shANO1_3
UT-SCC-8 shANO1_1
UT-SCC-8 shScramble_3
UT-SCC-8 shScramble_1
UT-SCC-8 shScramble_2

Figure S3

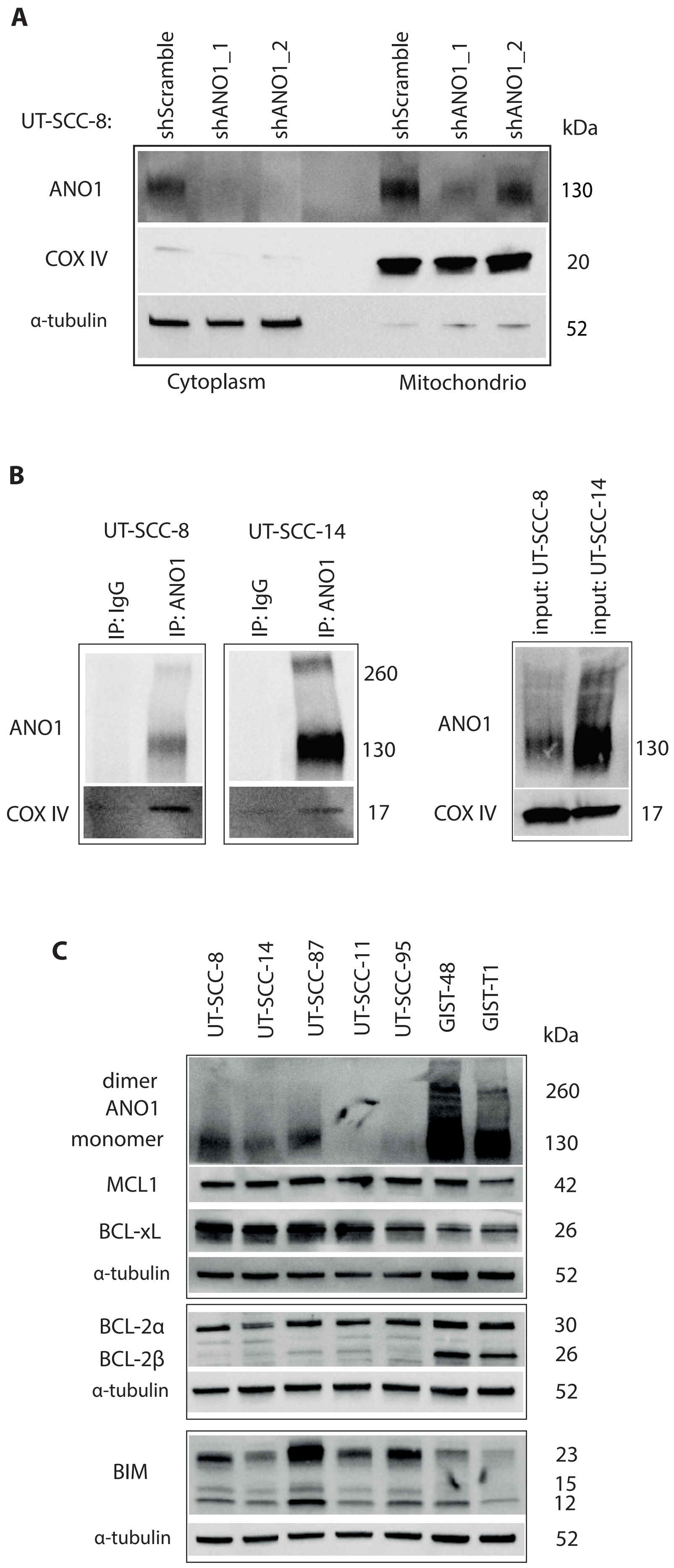
A
UT-SCC-8:
shScramble
shANO1_1
shANO1_2
shScramble
shANO1_1
shANO1_2
kDa
ANO1
130
COX IV
20
α-tubulin
52
Cytoplasm
Mitochondrio
B
UT-SCC-8
UT-SCC-14
IP: IgG
IP: ANO1
IP: IgG
IP: ANO1
input: UT-SCC-8
input: UT-SCC-14
ANO1
260
130
COX IV
17
ANO1
130
COX IV
17
C
UT-SCC-8
UT-SCC-14
UT-SCC-87
UT-SCC-11
UT-SCC-95
GIST-48
GIST-T1
kDa
dimer
ANO1
monomer
260
130
MCL1
42
BCL-xL
26
α-tubulin
52
BCL-2α
30
BCL-2β
26
α-tubulin
52
BIM
23
15
12
α-tubulin
52

Figure S4

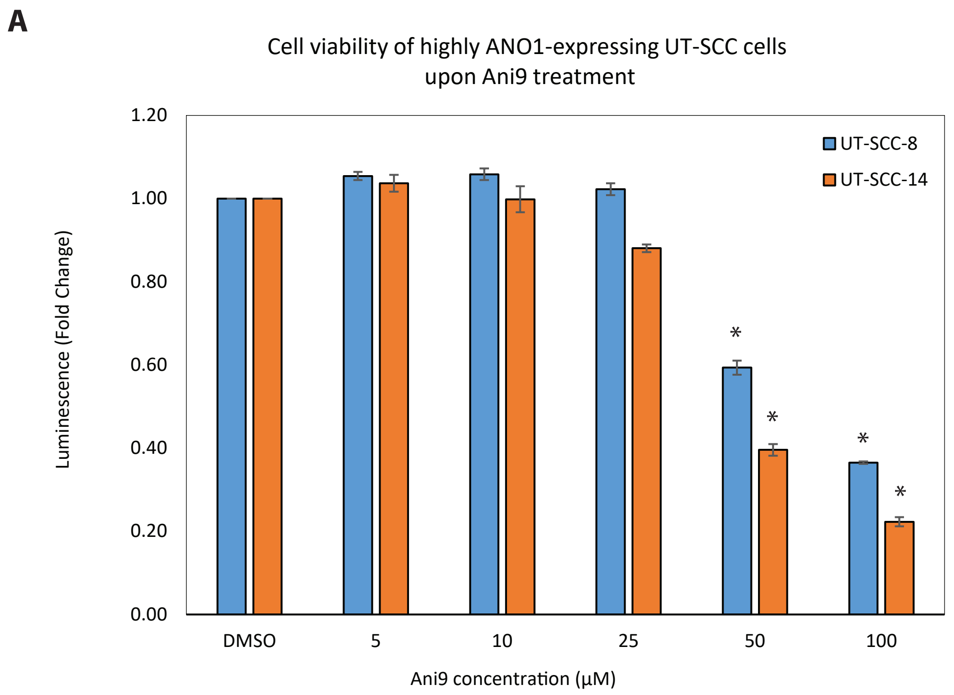

A
Cell viability of highly ANO1-expressing UT-SCC cells
upon Ani9 treatment
Luminescence (Fold Change)
1.20
1.00
0.80
0.60
0.40
0.20
0.00
UT-SCC-8
UT-SCC-14
*
DMSO
5
10
25
50
100
Ani9 concentration (µM)

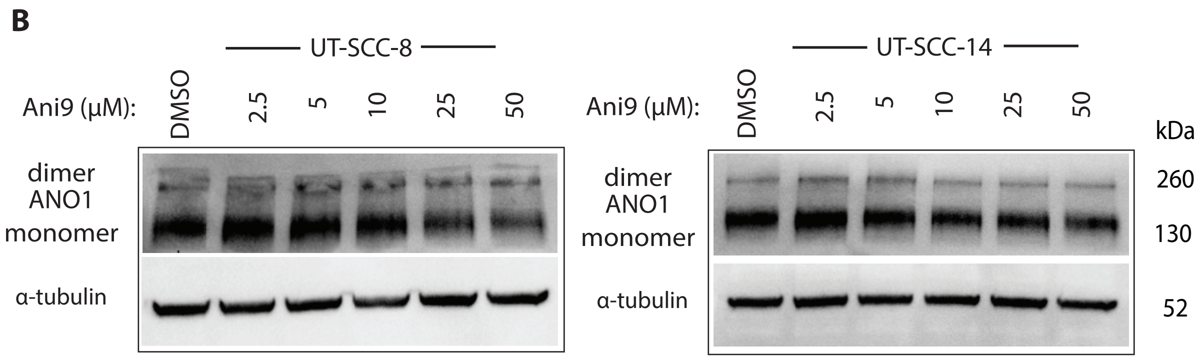

B
UT-SCC-8
UT-SCC-14
Ani9 (µM):
DMSO
2.5
5
10
25
50
dimer
ANO1
monomer
α-tubulin
kDa
260
130
52

Figure S5

A

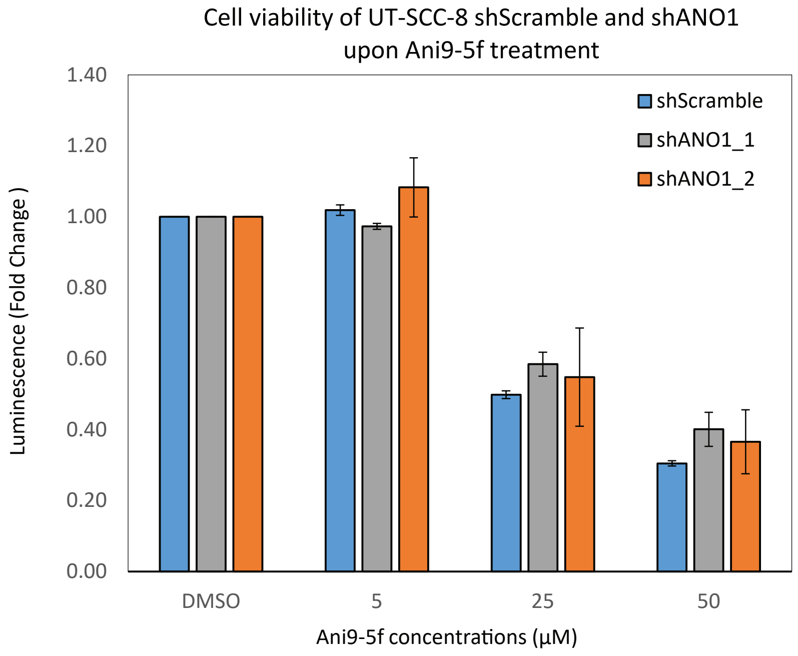
Cell viability of UT-SCC-8 shScramble and shANO1
upon Ani9-5f treatment
Luminescence (Fold Change)
1.40
1.20
1.00
0.80
0.60
0.40
0.20
0.00
shScramble
shANO1_1
shANO1_2
DMSO
5
25
50
Ani9-5f concentrations (μM)

B

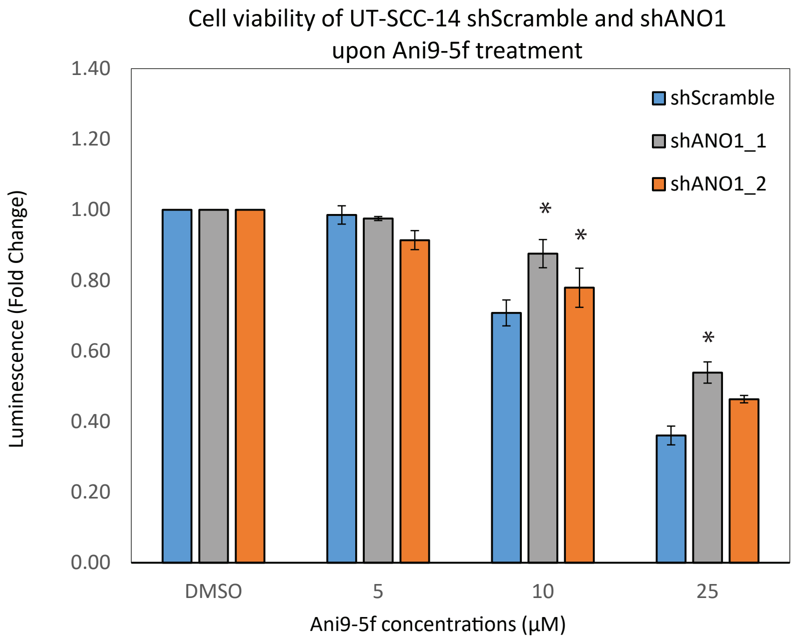
Cell viability of UT-SCC-14 shScramble and shANO1
upon Ani9-5f treatment
Luminescence (Fold Change)
1.40
1.20
1.00
0.80
0.60
0.40
0.20
0.00
shScramble
shANO1_1
shANO1_2
*
*
*
DMSO
5
10
25
Ani9-5f concentrations (μM)

Figure S6

A

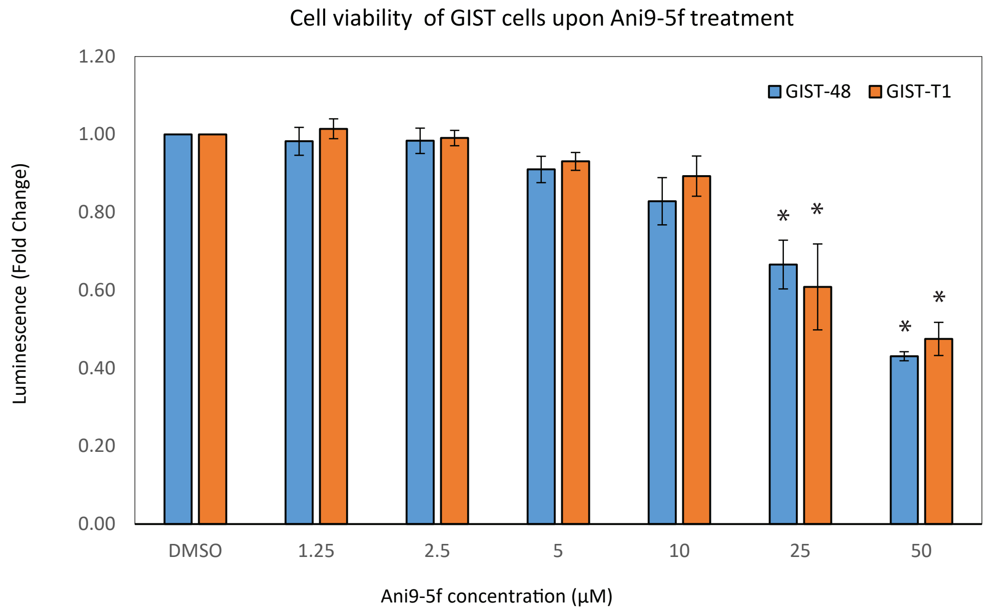

Cell viability of GIST cells upon Ani9-5f treatment
GIST-48
GIST-T1
Luminescence (Fold Change)
1.20
1.00
0.80
0.60
0.40
0.20
0.00
DMSO
1.25
2.5
5
10
25
50
Ani9-5f concentration (µM)
*

B

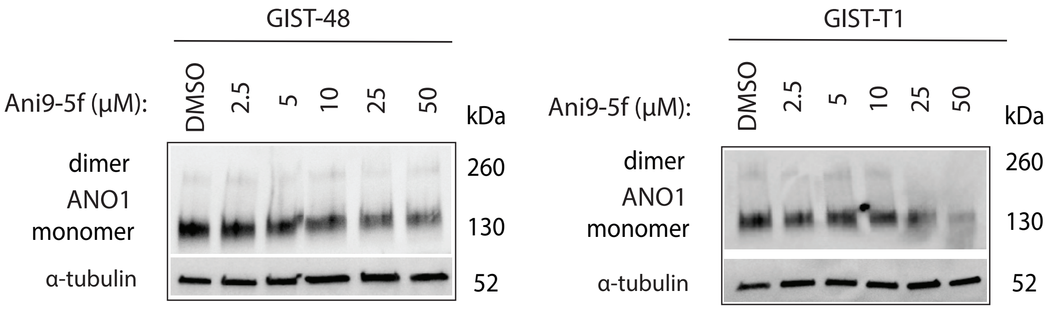

GIST-48
GIST-T1
Ani9-5f (µM):
DMSO
2.5
5
10
25
50
kDa
dimer
ANO1
monomer
α-tubulin
260
130
52

Figure S7 Uncropped blots of Figure 1A.

Immunoblot analysis of the proteins ANO1 and a-tubulin from a panel of ANO1-high/-low UT-SCC cell lines. Cell lines were selected based on their varying ANO1 mRNA expression (see Figure S1 and Lepikhova et al., 2018). Alpha-tubulin was used as the loading control to normalize the quantities of the proteins. Immunoblot figures, densitometry readings and normalized values are presented in the table below.

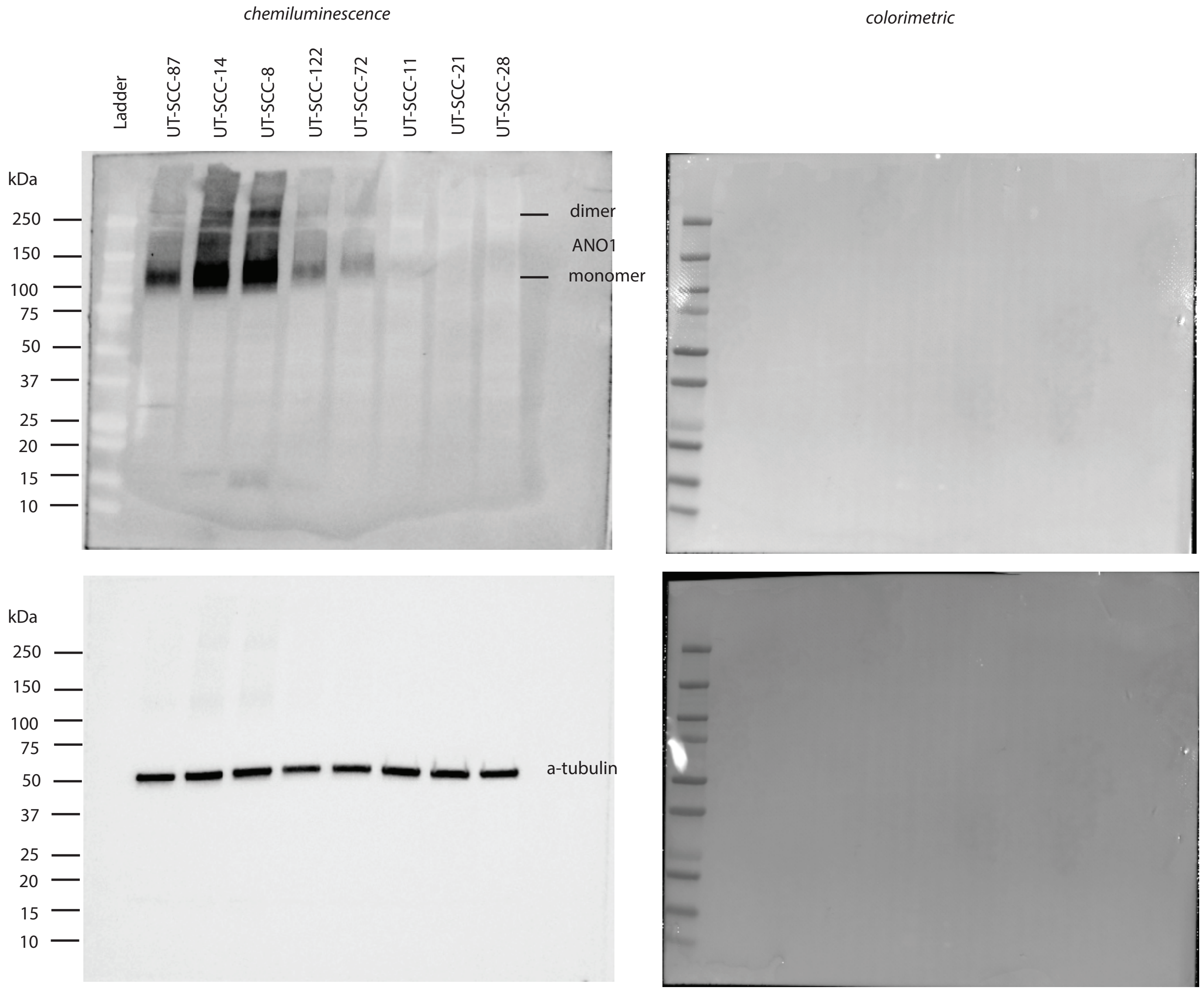

	Intensity of each band (background adjusted)							
	UT-SCC-87	UT-SCC-14	UT-SCC-8	UT-SCC-122	UT-SCC-72	UT-SCC-11	UT-SCC-21	UT-SCC-28
ANO1	2122120	4222667	4081735	1125580	719798	92608	25521	16267
α-tubulin	2167346	2171457	2001814	1308144	1489957	1881457	1921531	1677118
Intensity ratio	0.98	1.94	2.04	0.86	0.48	0.05	0.01	0.01

Figure S7 Uncropped blots of Figure 1C.
Immunoblot analysis of the proteins E-cadherin, N-cadherin, BMI1in cell lines UT-SCC-8 shScramble and shANO1_1, shANO1_2, as well as UT-SCC-14 shScramble and shANO1_1, shANO1_2. Alpha-tubulin or GAPDH were used as the loding controls to normalize the quantities of the proteins. Densitometry readings and normalized values are presented in the table under the whole blots.

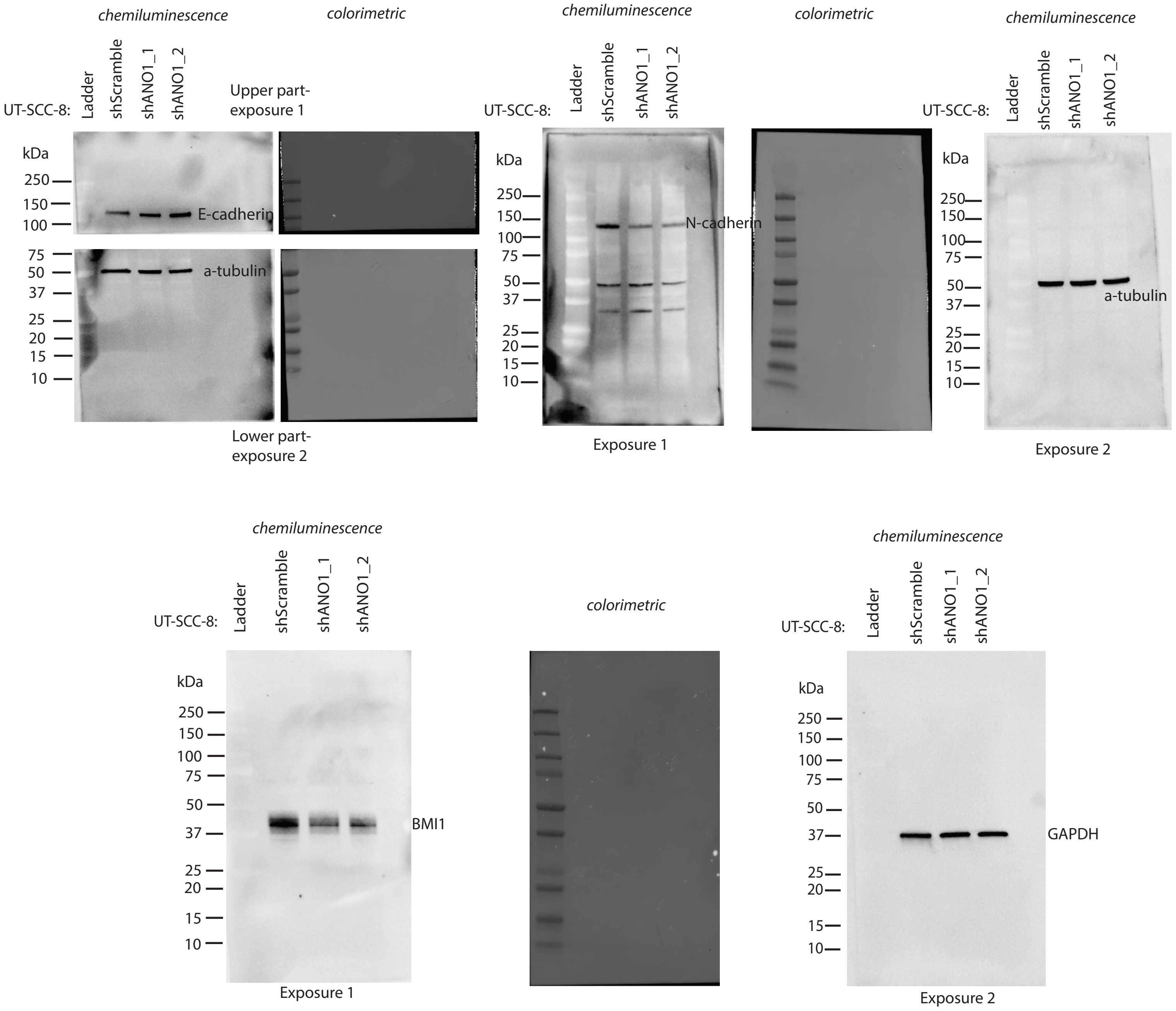

	Intensity of each band (background adjusted)		
	UT-SCC-8 shScramble	UT-SCC-8 shANO1_1	UT-SCC-8 shANO1_2
E-cadherin	754248	1019701	1559675
α-tubulin	1196417	1497757	1369201
Intensity ratio	0.63	0.68	1.14
N-cadherin	722962	16034	3697
α-tubulin	1517226	1558147	1609075
Intensity ratio	0.477	0.010	0.002
BMI1	4807371	2355650	2474908
α-tubulin	1587335	1742467	1626584
Intensity ratio	3.03	1.35	1.52

Figure S7 Uncropped blots of Figure 1C.

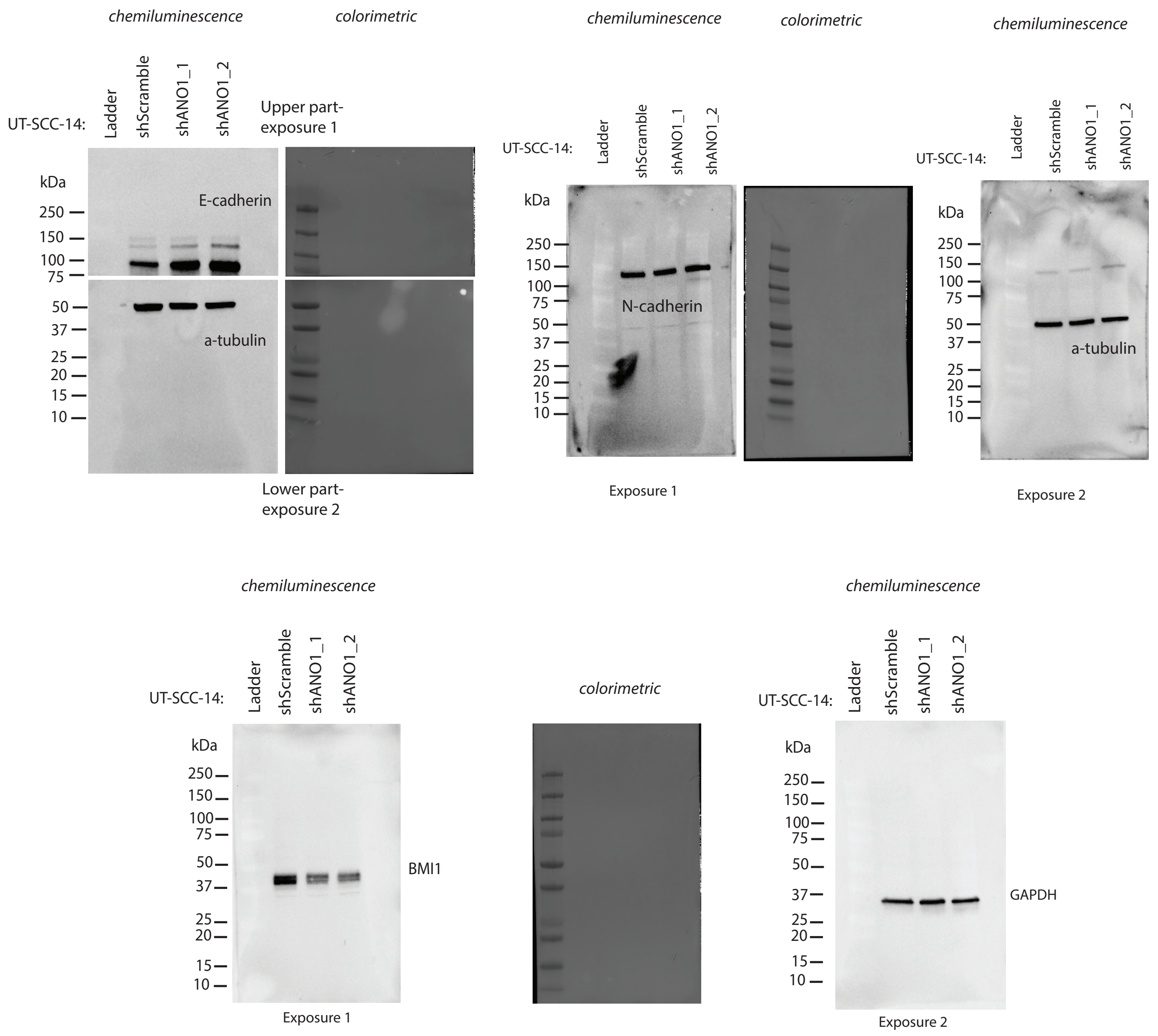

	Intensity of each band (background adjusted)		
	UT-SCC-14 shScramble	UT-SCC-14 shANO1_1	UT-SCC-14 shANO1_2
E-cadherin	1123628	2484411	3173673
α-tubulin	2054479	1696848	1854173
Intensity ratio	0.55	1.46	1.71
N-cadherin	1129594	835372	1641356
α-tubulin	1819949	1381151	1466748
Intensity ratio	0.62	0.60	1.12
BMI1	4752423	2702459	2518232
GAPDH	1926586	1840563	1691702
Intensity ratio	2.47	1.47	1.49

Figure S7 Uncropped blots of Figure 2B.
Immunoblot analysis of the proteins MCL-1, BCL-2, BCL-XL and BIM in cell lines UT-SCC-8 shScramble and shANO1_1, shANO1_2, as well as UT-SCC-14 shScramble and shANO1_1, shANO1_2. Alpha-tubulin was used as the loading control to normalize the quantities of the proteins. Densitometry readings and normalized values are presented in the tables under the respective figures.

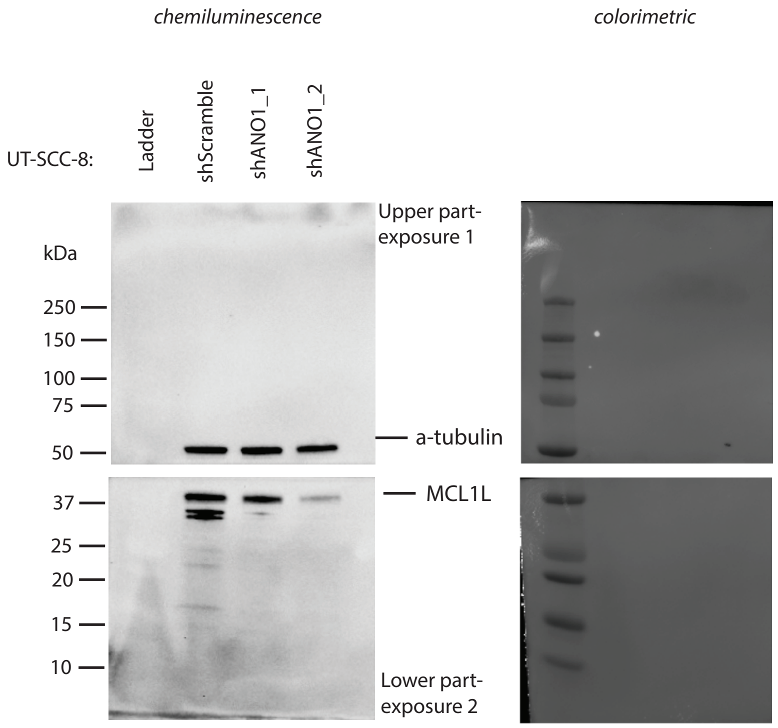

	Intensity of each band (background adjusted)		
	UT-SCC-8 shScramble	UT-SCC-8 shANO1_1	UT-SCC-8 shANO1_2
MCL1L	1379494	1043672	315049
α-tubulin	2163007	2363063	1671994
Intensity ratio	0.64	0.44	0.19

Figure S7 Uncropped blots of Figure 2B.

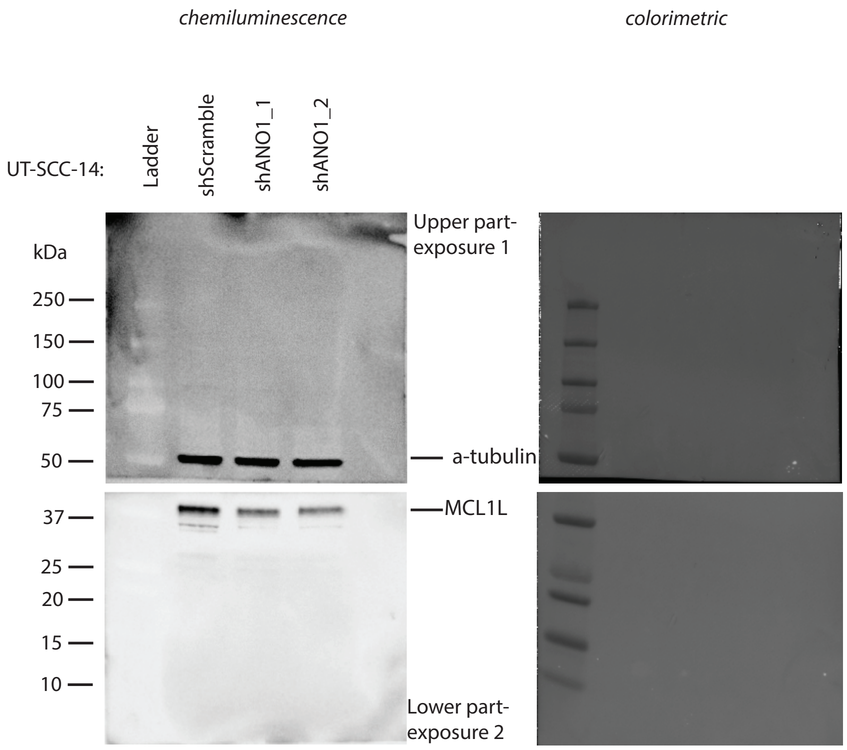

	Intensity of each band (background adjusted)		
	UT-SCC-14 shScramble	UT-SCC-14 shANO1_1	UT-SCC-14 shANO1_2
MCL1L	1748141	747115	617846
α-tubulin	1783245	1259613	996603
Intensity ratio	0.98	0.59	0.62

Figure S7 Uncropped blots of Figure 2B.

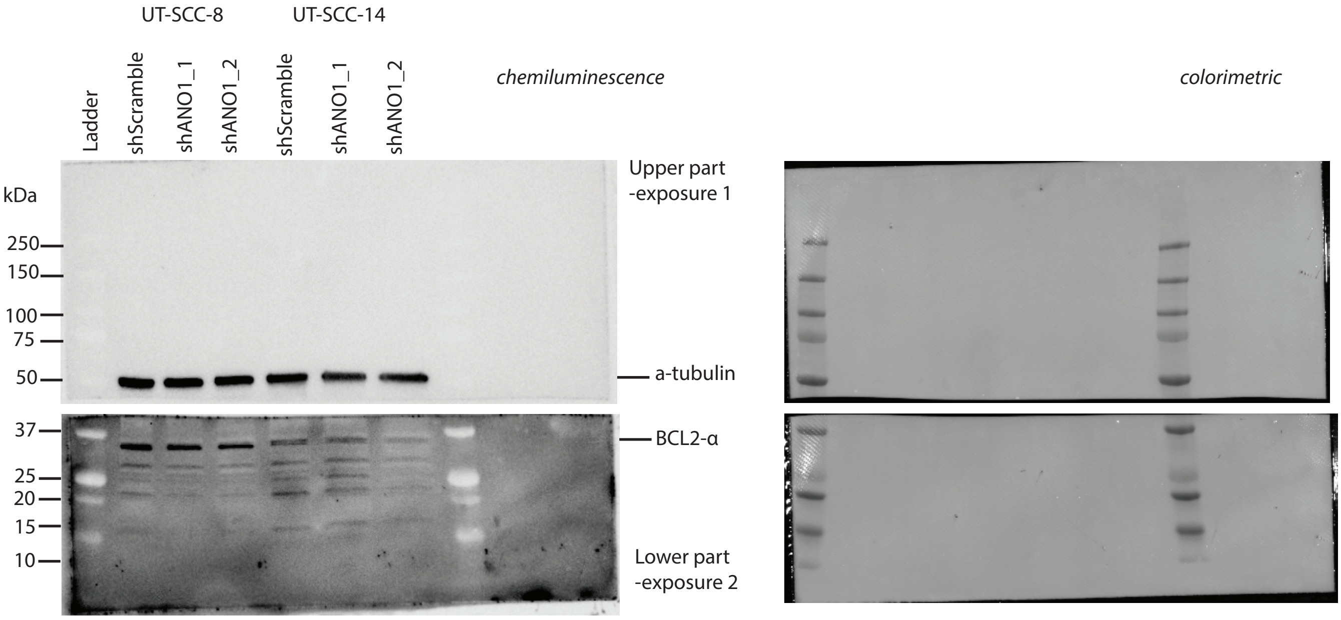

	Intensity of each band (background adjusted)					
	UT-SCC-8 shScramble	UT-SCC-8 shANO1_1	UT-SCC-8 shANO1_2	UT-SCC-14 shScramble	UT-SCC-14 shANO1_1	UT-SCC-14 shANO1_2
BCL2-α	770898	547671	495145	178472	136895	90218
α-tubulin	2302198	2155391	2034745	1694849	1371401	1413635
Intensity ratio	0.33	0.25	0.24	0.11	0.10	0.06

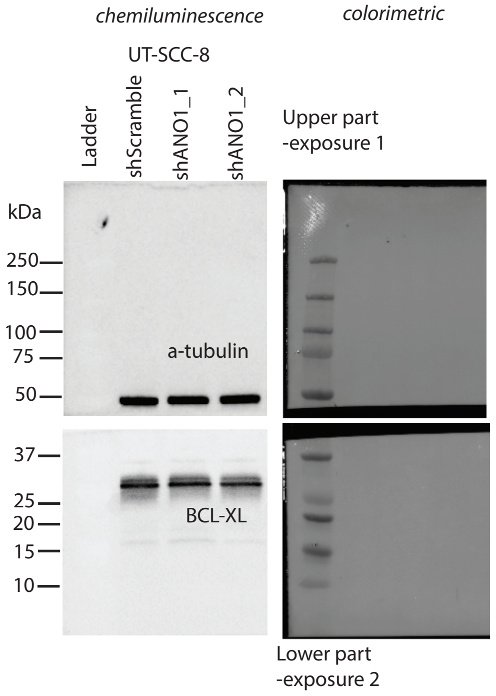

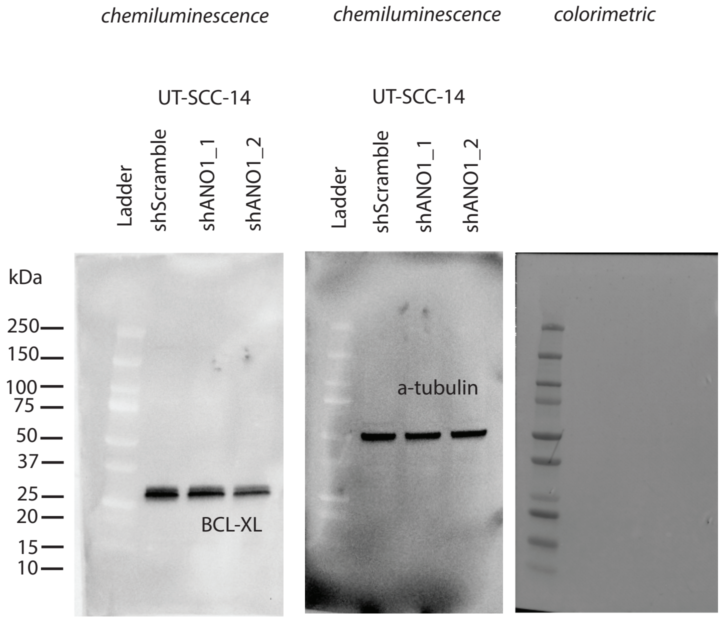

	Intensity of each band (background adjusted)		
	UT-SCC-8 shScramble	UT-SCC-8 shANO1_1	UT-SCC-8 shANO1_2
BCL-XL	3255706	2582922	2712684
α-tubulin	2068165	1959181	1760224
Intensity ratio	1.57	1.32	1.54

	Intensity of each band (background adjusted)		
	UT-SCC-14 shScramble	UT-SCC-14 shANO1_1	UT-SCC-14 shANO1_2
BCL-XL	2977950	2804066	2099190
α-tubulin	1371232	984975	1248328
Intensity ratio	2.17	2.85	1.68

Figure S7 Uncropped blots of Figure 2B.

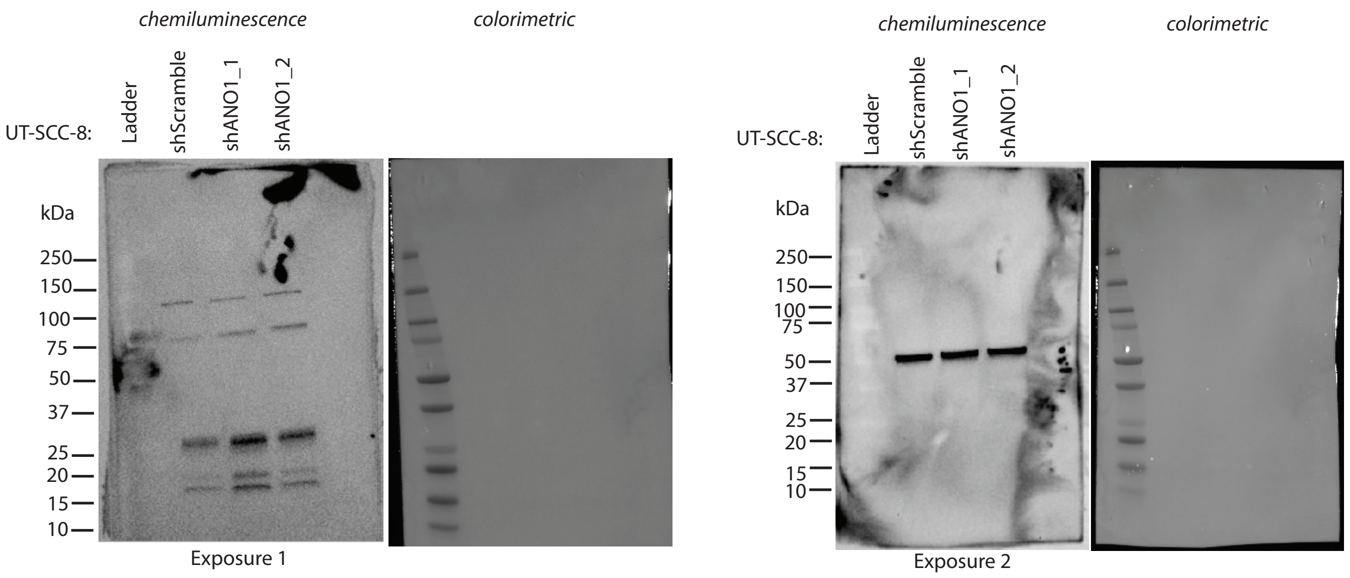

	Intensity of each band (background adjusted)		
	UT-SCC-8 shScramble	UT-SCC-8 shANO1_1	UT-SCC-8 shANO1_2
BIM	129874	261924	221288
α-tubulin	1281083	1182915	1069172
Intensity ratio	0.10	0.22	0.21

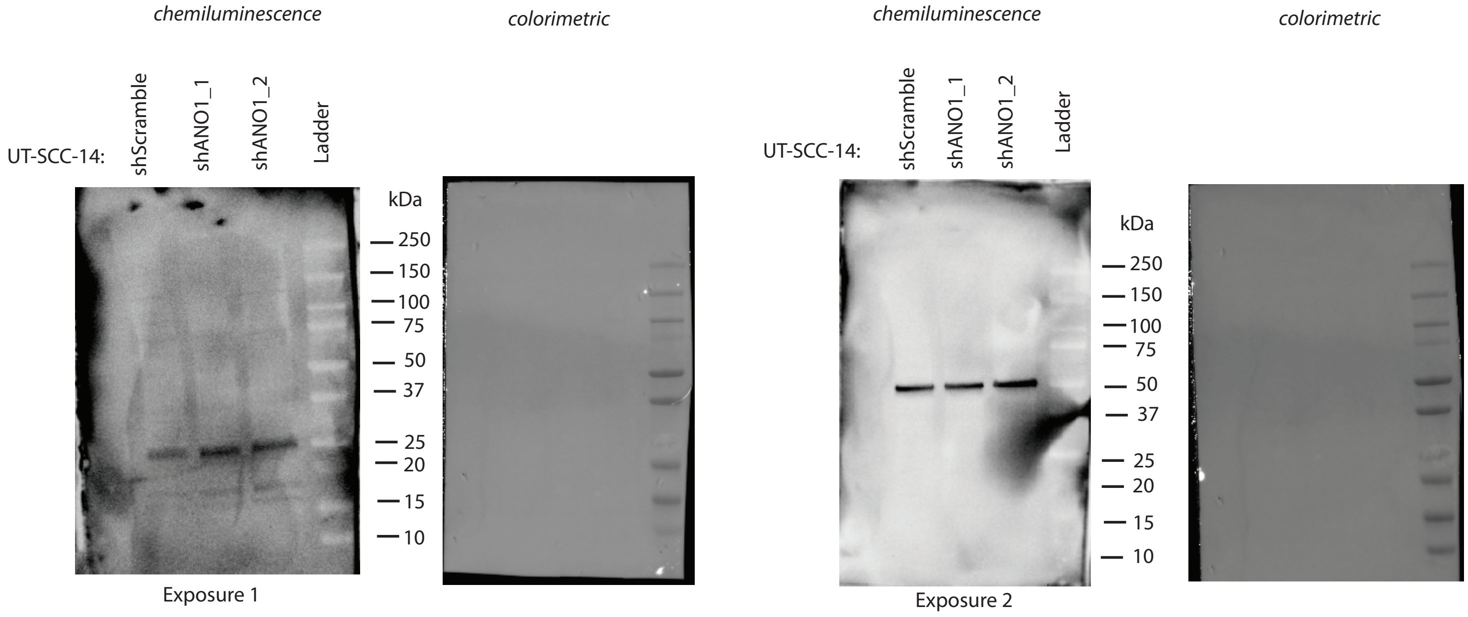

	Intensity of each band (background adjusted)		
	UT-SCC-14 shScramble	UT-SCC-14 shANO1_1	UT-SCC-14 shANO1_2
BIM	152245	285054	326040
α-tubulin	1238184	1198241	1410510
Intensity ratio	0.12	0.24	0.23

Figure S7 Uncropped blots from Figure 4A.

Immunoblot analysis of the protein CDKN1B (p27kip1) in cell lines UT-SCC-8 shScramble and shANO1_1, shANO1_2, as well as UT-SCC-14 shScramble and shANO1_1, shANO1_2. Alpha-tubulin was used as the control to normalize the quantities of the proteins. Densitometry readings and normalized values are presented in the tables under the respective whole blots.

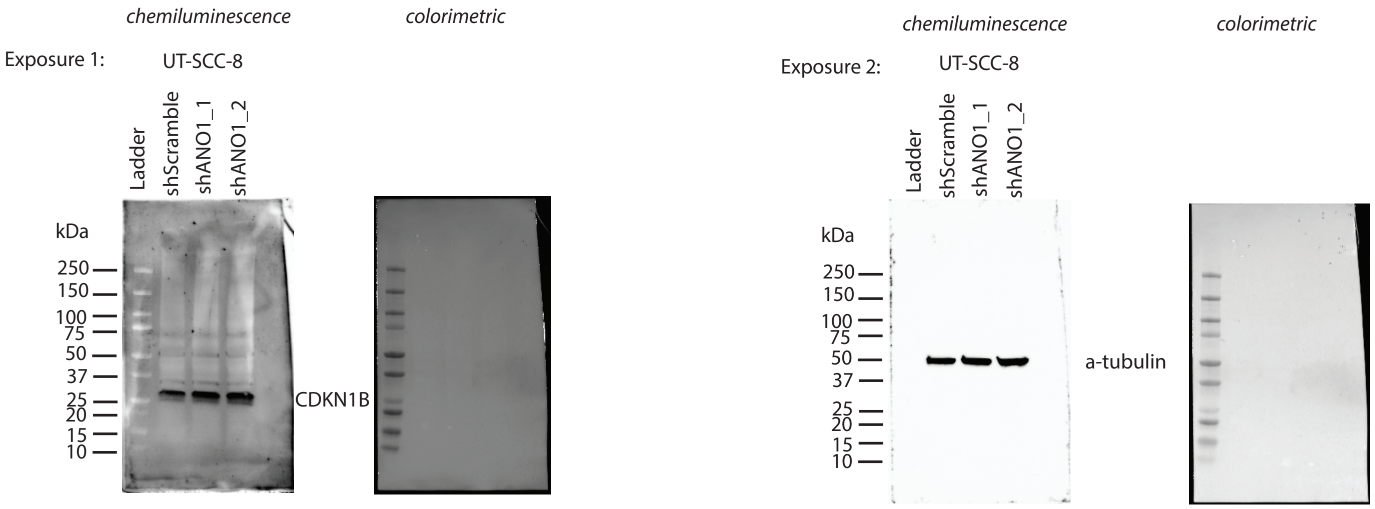

	UT-SCC-8 shScramble	UT-SCC-8 shANO1_1	UT-SCC-8 shANO1_2
CDKN1B	6954665	9387748	10598253
α-tubulin	14466840	16481549	17634530
Intensity ratio	0.48	0.57	0.60

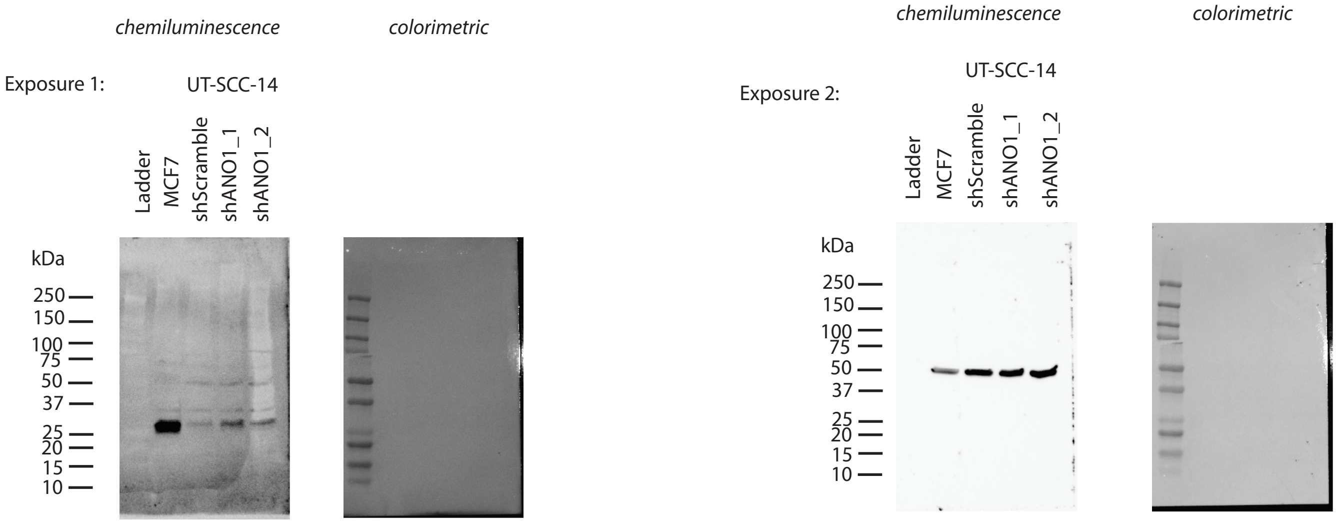

		Intensity of each band (background adjusted)		
	MCF7	UT-SCC-14 shScramble	UT-SCC-14 shANO1_1	UT-SCC-14 shANO1_2
CDKN1B	1465825	129064	330047	198153
α-tubulin	2625200	5544654	5449901	7602643
Intensity ratio	0.56	0.02	0.06	0.03

*MCF7 was used as positive control for the antibody

Figure S7 Addition uncropped blots (not included in Main Figure), showing the same result as Figure 4A.
Immunoblot analysis of the protein CDKN1B (p27kip1) in cell lines UT-SCC-8 shScramble and shANO1_1, shANO1_2, as well as UT-SCC-14 shScramble and shANO1_1, shANO1_2. Alpha-tubulin was used as the control to normalize the quantities of the proteins. Densitometry readings and normalized values are presented in the tables under the whole blots.

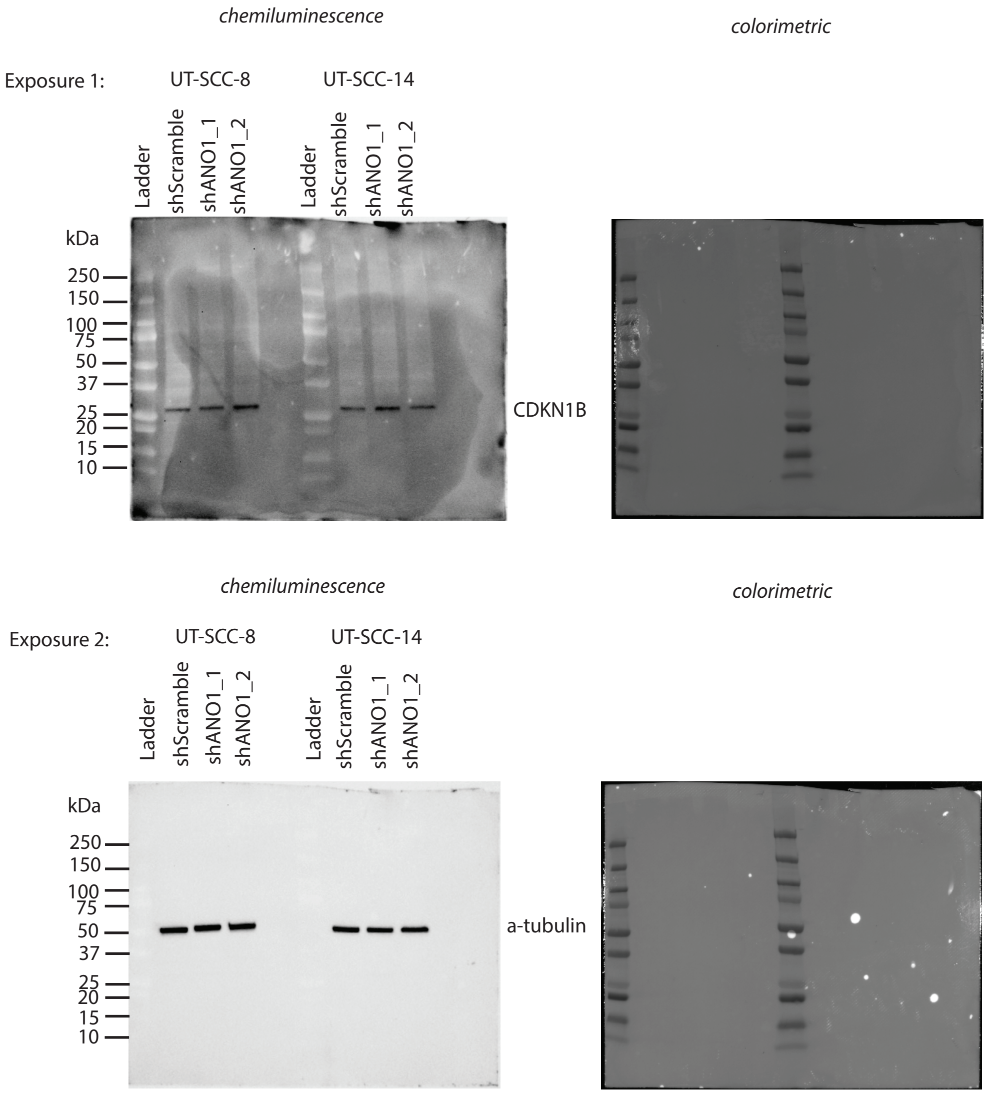

	Intensity of each band (background adjusted)					
	UT-SCC-8 shScramble	UT-SCC-8 shANO1_1	UT-SCC-8 shANO1_2	UT-SCC-14 shScramble	UT-SCC-14 shANO1_1	UT-SCC-14 shANO1_2
CDKN1B	126132	289606	613225	320905	657558	466882
α-tubulin	2317065	1972136	1882290	1681519	1461723	1454817
Intensity ratio	0.05	0.15	0.33	0.19	0.45	0.32

Figure S7 Uncropped blots of Figure 4B.
Immunoblot analysis of the proteins CDKN1B, H2A.x, a-tubulin, MCL1 in the cytoplasmic and nuclear fraction of the cell lines UT-SCC-8 shScramble and shANO1_1, shANO1_2, as well as UT-SCC-14 shScramble and shANO1_1, shANO1_2. Densitometry readings and normalized values are presented in the tables under the respective whole blots.

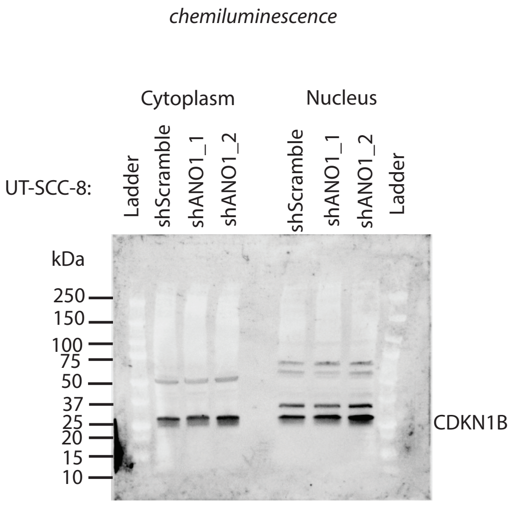

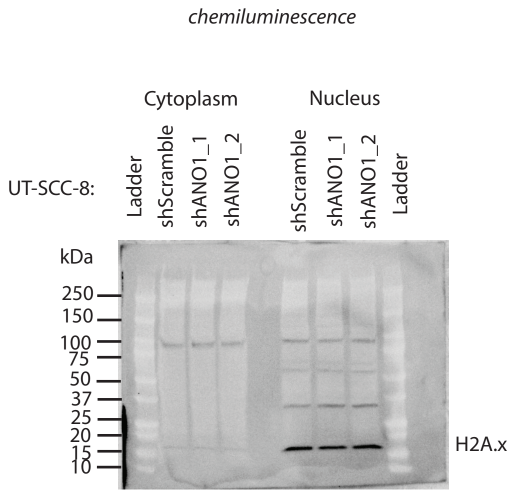

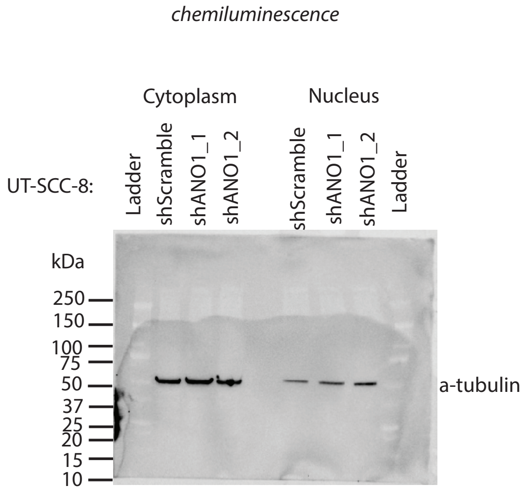

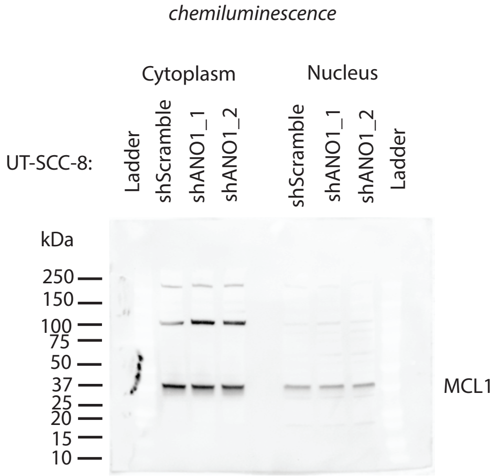

	Intensity of each band (background adjusted)					
	UT-SCC-8 shScramble cytoplasmic	UT-SCC-8 shANO1_1 cytoplasmic	UT-SCC-8 shANO1_2 cytoplasmic	UT-SCC-8 shScramble nuclear	UT-SCC-8 shANO1_1 nuclear	UT-SCC-8 shANO1_2 nuclear
CDKN1B	1613620	1726694	2195320	1775510	2390283	3665279
α-tubulin	1246482	1382003	1150454	289300	417286	468484
H2Ax	17221	25344	1668	605133	414028	562209
Intensity ratio (to a-tubulin)	1.29	1.25	1.91	6.14	5.73	7.82
Intensity ratio (to H2Ax)	93.70	68.13	1316.39	2.93	5.77	6.52
MCL1	881311	854091	636320	142116	63535	115539
Intensity ratio (to a-tubulin)	0.71	0.62	0.55	0.49	0.15	0.25
Intensity ratio (to H2Ax)	51.18	33.70	381.56	0.23	0.15	0.21

Figure S7 Uncropped blots of Figure 4B.

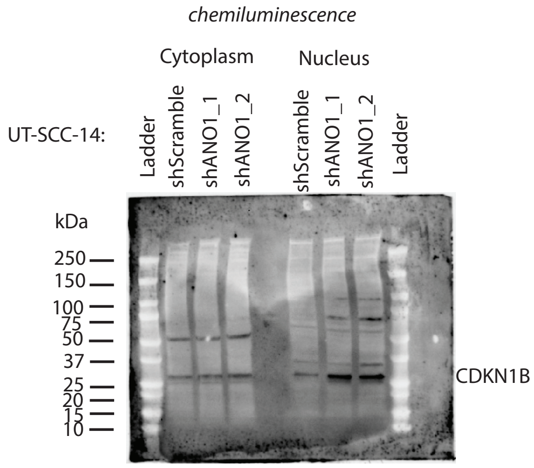

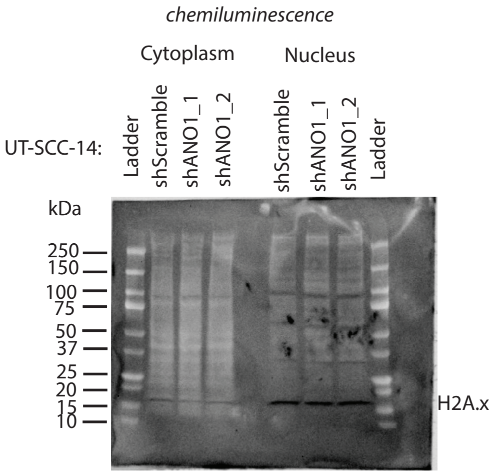

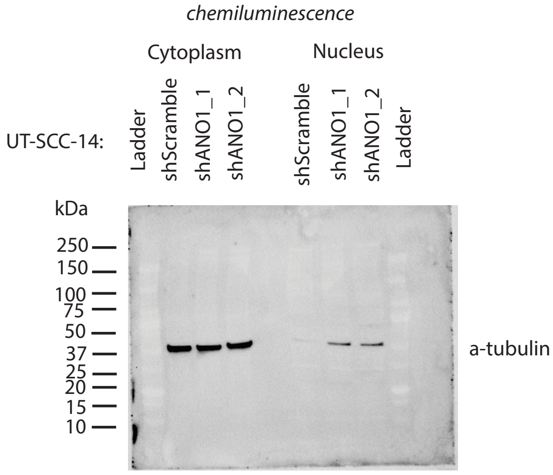

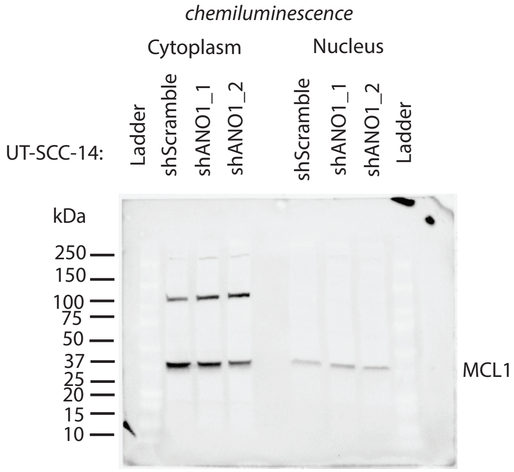

	Intensity of each band (background adjusted)					
	UT-SCC-14 shScramble cytoplasmic	UT-SCC-14 shANO1_1 cytoplasmic	UT-SCC-14 shANO1_2 cytoplasmic	UT-SCC-14 shScramble nuclear	UT-SCC-14 shANO1_1 nuclear	UT-SCC-14 shANO1_2 nuclear
CDKN1B	251426	426953	620078	358407	1449579	1448372
α-tubulin	2060382	1483864	1791450	1430	300191	348997
H2Ax	7683	11107	241691	2962334	945262	1820972
Intensity ratio (to a-tubulin)	0.12	0.29	0.35	250.71	4.83	4.15
Intensity ratio (to H2Ax)	32.72	38.44	2.57	0.12	1.53	0.80
MCL1	574987	547793	318055	28143	90272	105174
Intensity ratio (to a-tubulin)	0.28	0.37	0.18	19.69	0.30	0.30
Intensity ratio (to H2Ax)	74.84	49.32	1.32	0.01	0.10	0.06

Figure S7 Uncropped blots of Figure 5A.

Immunoblot analysis of ANO1 and a-tubulin upon treatment with a range of concentrations of Ani9-5f inhibitor. Cell lines used were UT-SCC-8 and UT-SCC-14. Densitometry readings and normalized values are presented in the tables under the respective figures.

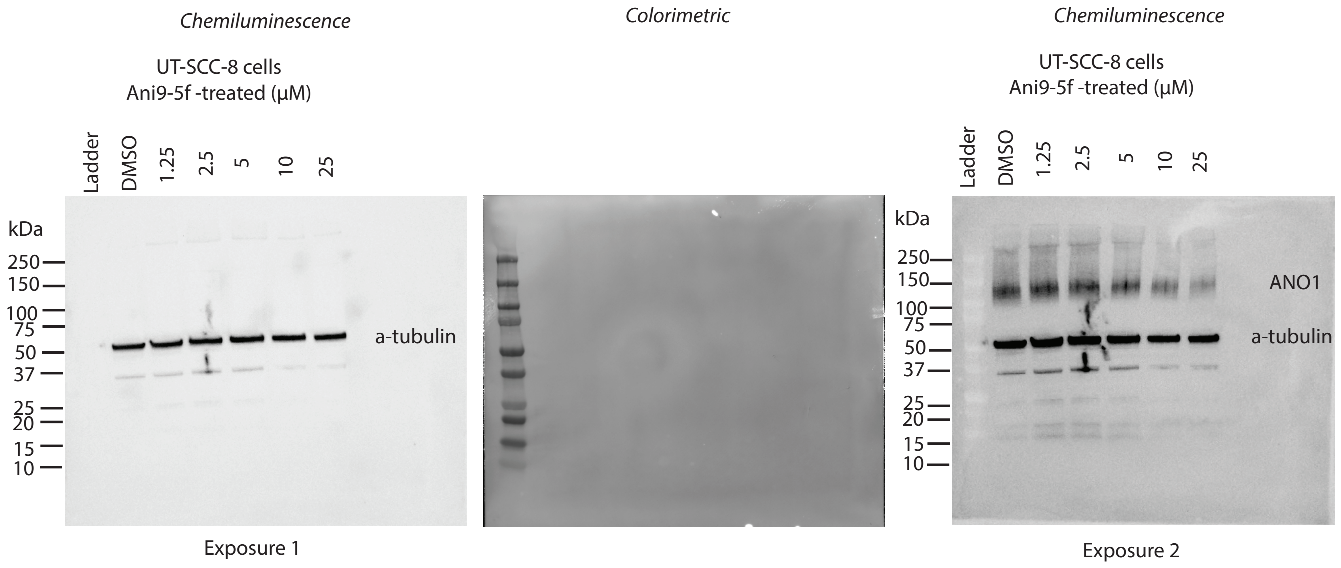

	Intensity of each band (background adjusted)					
	UT-SCC-8 DMSO	UT-SCC-8 1.25 µM	UT-SCC-8 2.5 µM	UT-SCC-8 5 µM	UT-SCC-8 10 µM	UT-SCC-8 25 µM
ANO1 (monomer)	1413815	1505826	1255117	1224846	857044	597264
a-tubulin	2036621	2279962	2613640	2682535	2508756	2262548
Intensity ratio (to a-tubulin)	0.69	0.66	0.48	0.46	0.34	0.26

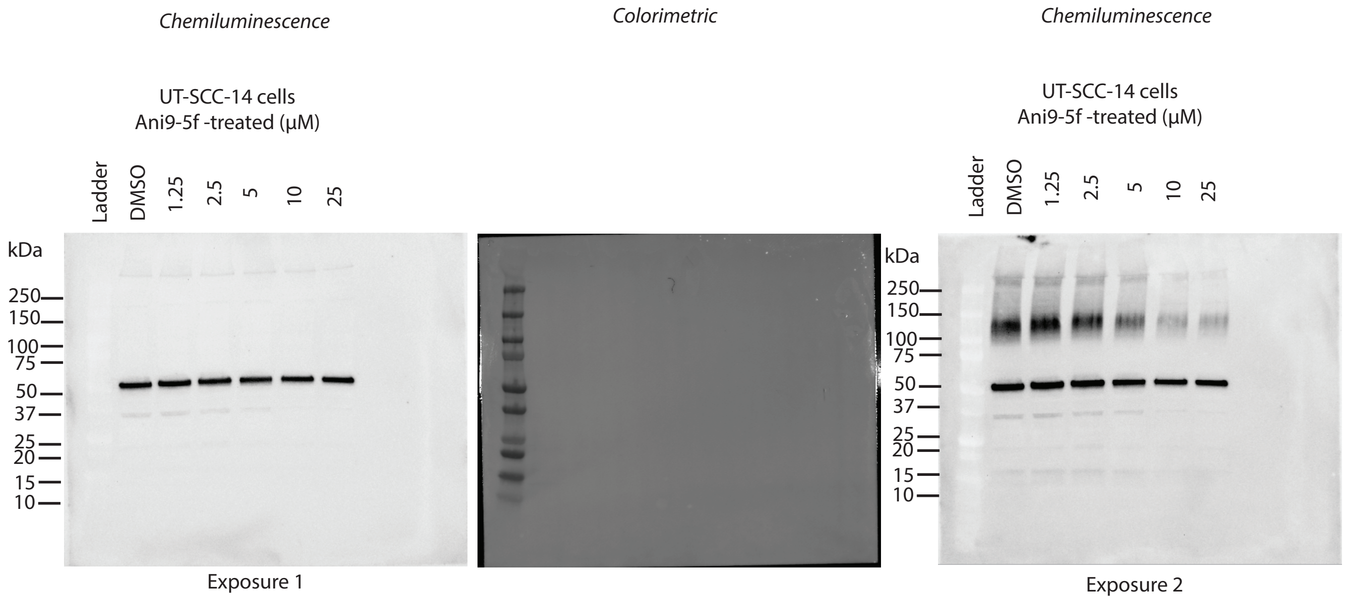

	Intensity of each band (background adjusted)					
	UT-SCC-14 DMSO	UT-SCC-14 1.25 µM	UT-SCC-14 2.5 µM	UT-SCC-14 5 µM	UT-SCC-14 10 µM	UT-SCC-14 25 µM
ANO1 (monomer)	6109486	6747155	5586846	3730226	1799467	1720249
a-tubulin	3502227	3223582	2766304	2624671	2579560	2943333
Intensity ratio (to a-tubulin)	1.74	2.09	2.02	1.42	0.70	0.58

Figure S7 Uncropped blots of Figure 5C.
Immunoblot analysis of changes in proteins ANO1, MCL1, BCL-XL and a-tubulin upon treatment with a range of concentrations of Ani9-5f inhibitor. Cell lines used were UT-SCC-8 and UT-SCC-14. Densitometry readings and normalized values are presented in the tables under the respective whole blots.

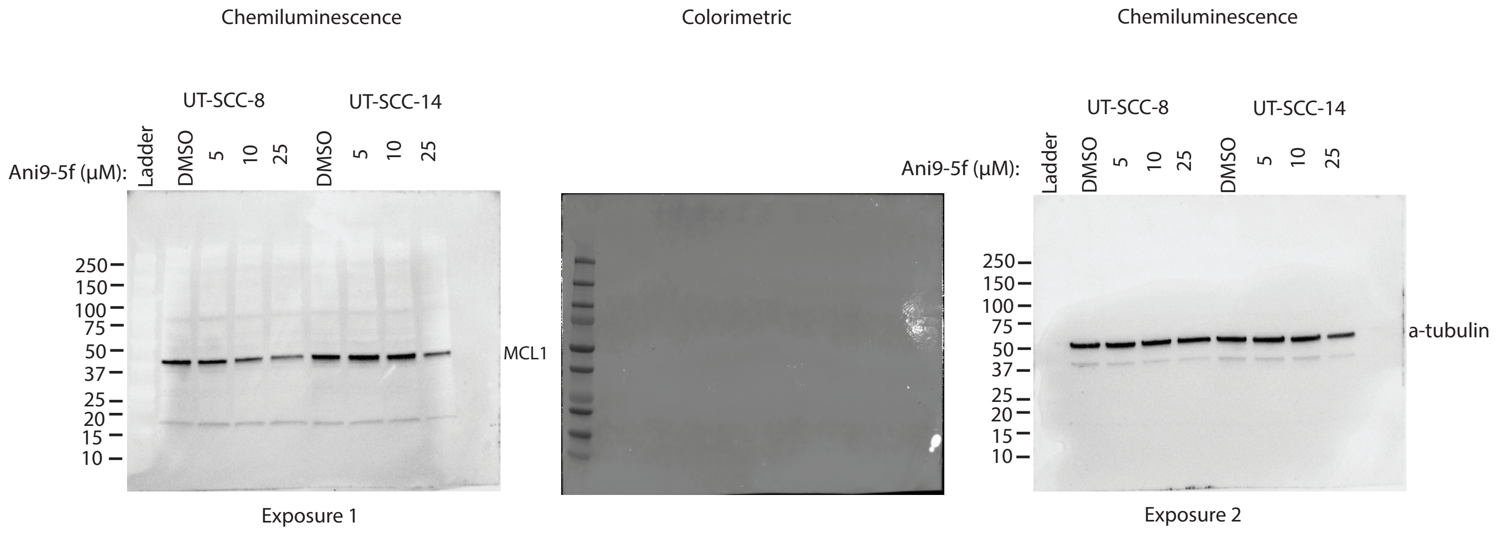

	Intensity of each band (background adjusted)							
	UT-SCC-8 DMSO	UT-SCC-8 5 µM	UT-SCC-8 10 µM	UT-SCC-8 25 µM	UT-SCC-14 DMSO	UT-SCC-14 5 µM	UT-SCC-14 10 µM	UT-SCC-14 25 µM
MCL1	1643657	1336736	903073	740553	1606465	1938292	1870507	783287
a-tubulin	1826987	2126834	2015056	1881652	1947402	2064488	1888266	1242886
Intensity ratio (MCL1/ a-tubulin)	0.90	0.63	0.45	0.39	0.82	0.94	0.99	0.63

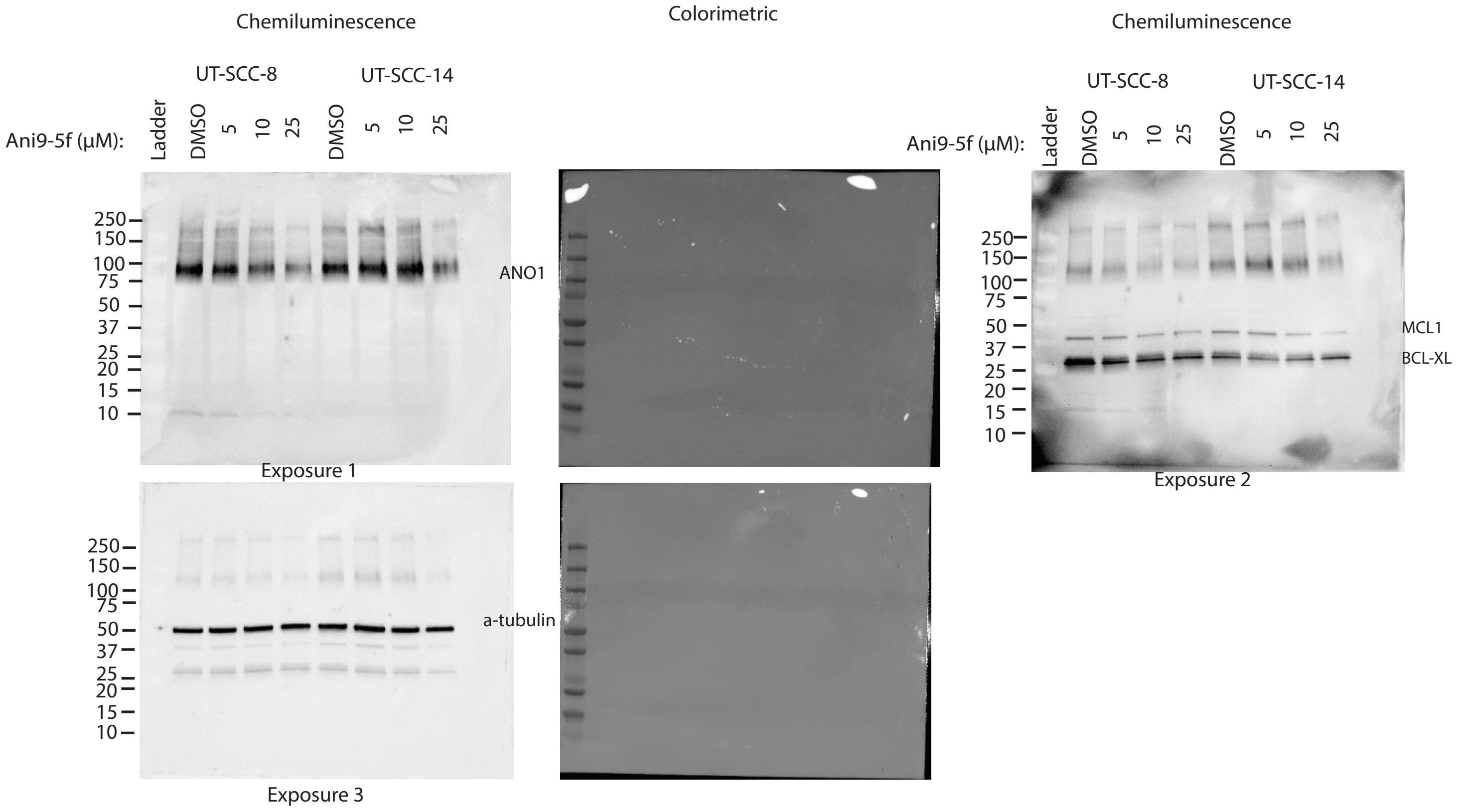

	Intensity of each band (background adjusted)							
	UT-SCC-8 DMSO	UT-SCC-8 5 µM	UT-SCC-8 10 µM	UT-SCC-8 25 µM	UT-SCC-14 DMSO	UT-SCC-14 5 µM	UT-SCC-14 10 µM	UT-SCC-14 25 µM
ANO1 (monomer)	3616714	3029903	2351941	1531517	3348691	3584370	3683543	2046164
BCL-XL	3118159	2293000	2184385	2045446	1884100	1462271	1367475	1392241
a-tubulin	1941652	2068715	2093396	2152450	2101054	2435730	2087683	1726260
Intensity ratio (ANO1/ a-tubulin)	1.86	1.46	1.12	0.71	1.59	1.47	1.76	1.19
Intensity ratio (BCL-XL/ a-tubulin)	1.61	1.11	1.04	0.95	0.90	0.60	0.66	0.81

Figure S7 Uncropped blots of Figure 6B.

Immunoblot analysis of the proteins MCL-1, BCL-XL and a-tubulin from cell lysates of UT-SCC-8 and UT-SCC-14 treated with a range of concentrations of AZD-5991 inhibitor. Alpha-tubulin was used as the control to normalize the quantities of the proteins. Densitometry readings and normalized values are presented in the tables under the respective figures.

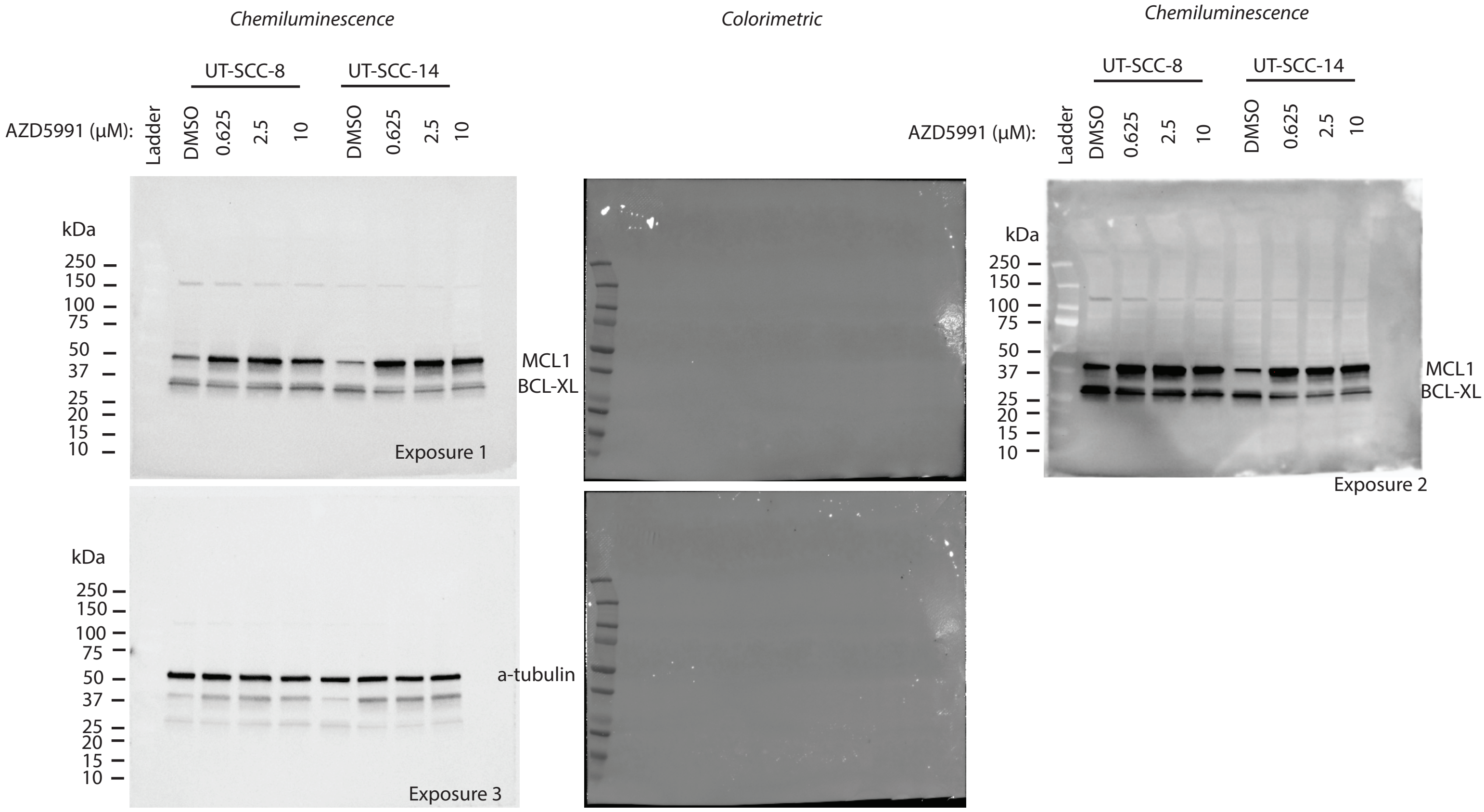

AZD-5991-treated	Intensity of each band (background adjusted)							
	UT-SCC-8 DMSO	UT-SCC-8 0.625 μM	UT-SCC-8 2.5 μM	UT-SCC-8 10 μM	UT-SCC-14 DMSO	UT-SCC-14 0.625 μM	UT-SCC-14 2.5 μM	UT-SCC-14 10 μM
MCL1	674694	1607502	2045739	1519667	577321	2225212	1972098	2007980
BCL-XL	7978149	5708129	7218642	7232210	6729603	4966260	3572365	4846508
α-tubulin	1961259	2071485	1889649	1740564	1730898	1963104	1591766	1856926
Intensity ratio (MCL1/a-tubulin)	0.34	0.78	1.08	0.87	0.33	1.13	1.24	1.08
Intensity ratio (BCL-XL/a-tubulin)	4.07	2.76	3.82	4.16	3.89	2.53	2.24	2.61

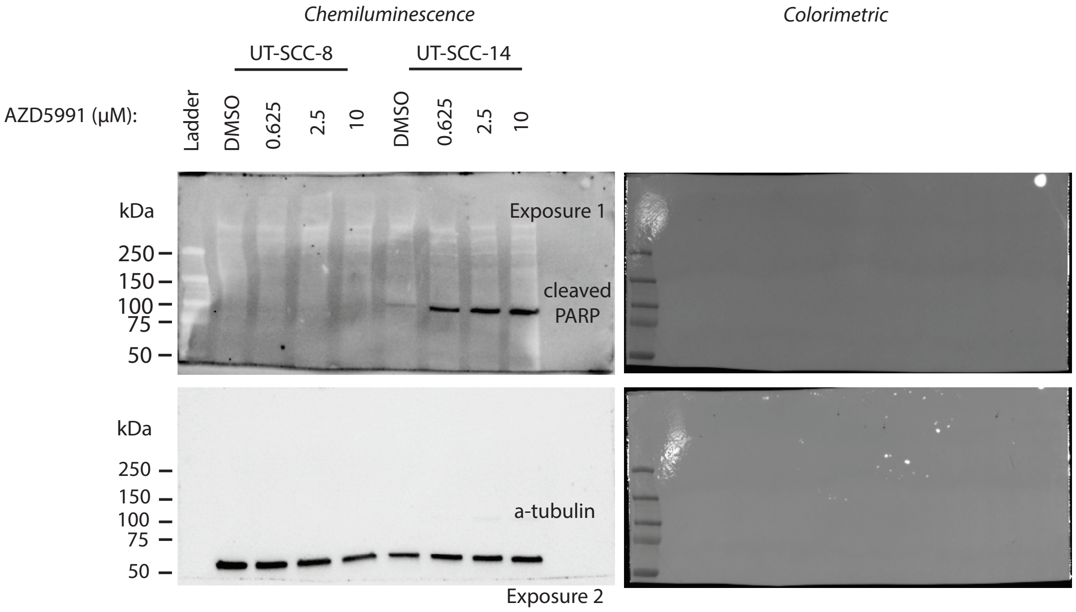

AZD-5991-treated	Intensity of each band (background adjusted)							
	UT-SCC-8 DMSO	UT-SCC-8 0.625 μM	UT-SCC-8 2.5 μM	UT-SCC-8 10 μM	UT-SCC-14 DMSO	UT-SCC-14 0.625 μM	UT-SCC-14 2.5 μM	UT-SCC-14 10 μM
Cleaved-PARP	-48534	-63401	-94361	10784	52453	515492	902968	1112333
α-tubulin	2441657	2155564	1765976	1409903	1320234	1602544	1554180	1673707
Intensity ratio (PARP/a-tubulin)	0.00	0.00	0.00	0.01	0.04	0.32	0.58	0.66

Figure S7 Uncropped blots of Figure S2A.
Immunoblot analysis shows ANO1 and a-tubulin protein levels following lentiviral transductions. Two different shRNA constructs targeting ANO1, as well as two technical replicates were tested. shScramble indicates control lentivirus constructs. Cell lines used were UT-SCC-8 and UT-SCC-14. Densitometry readings and normalized values are presented in the tables under the respective whole blots.

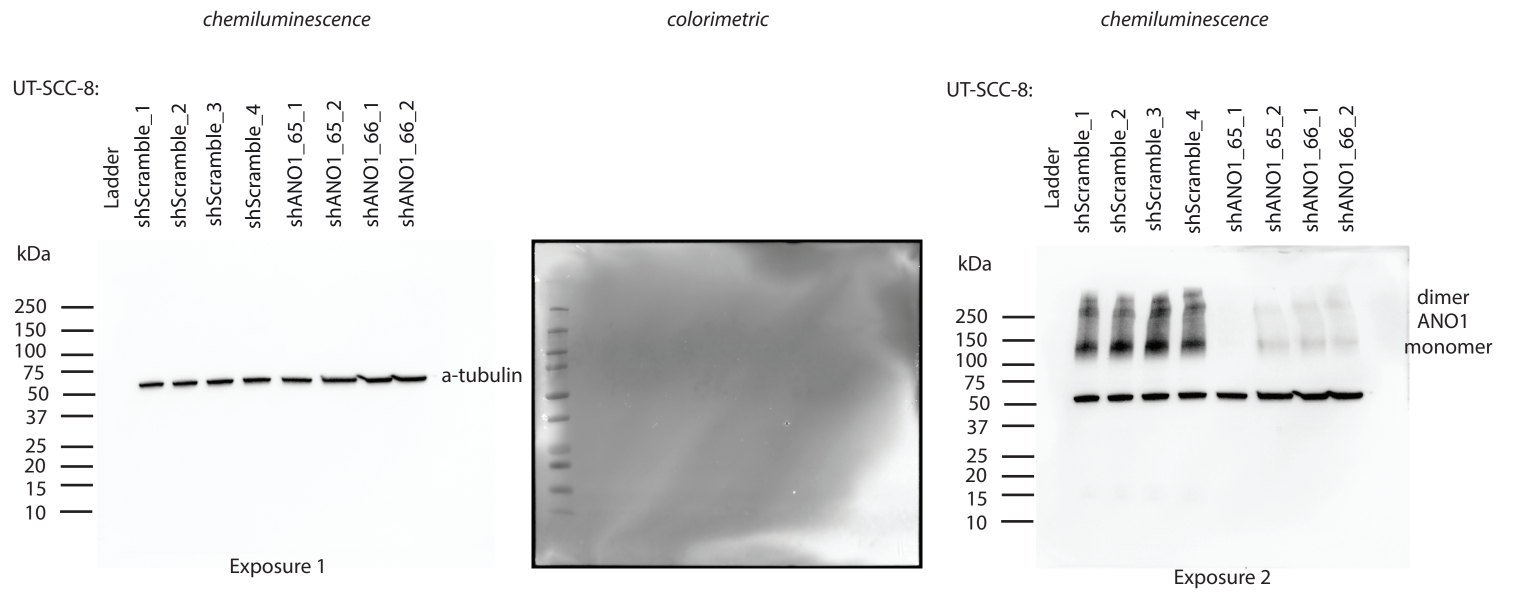

	Intensity of each band (background adjusted)							
	UT-SCC-8 shScramble-1	UT-SCC-8 shScramble-2	UT-SCC-8 shScramble-3	UT-SCC-8 shScramble-4	UT-SCC-8 shANO1_65-1	UT-SCC-8 shANO1_65-2	UT-SCC-8 shANO1_66-1	UT-SCC-8 shANO1_66-2
ANO1	12077201	13846237	14155545	10275488	137207	770529	1683514	1192792
α-tubulin	11312051	11827842	14064242	14297874	14445017	14808878	15155762	16039669
Intensity ratio	1.07	1.17	1.01	0.72	0.01	0.05	0.11	0.07

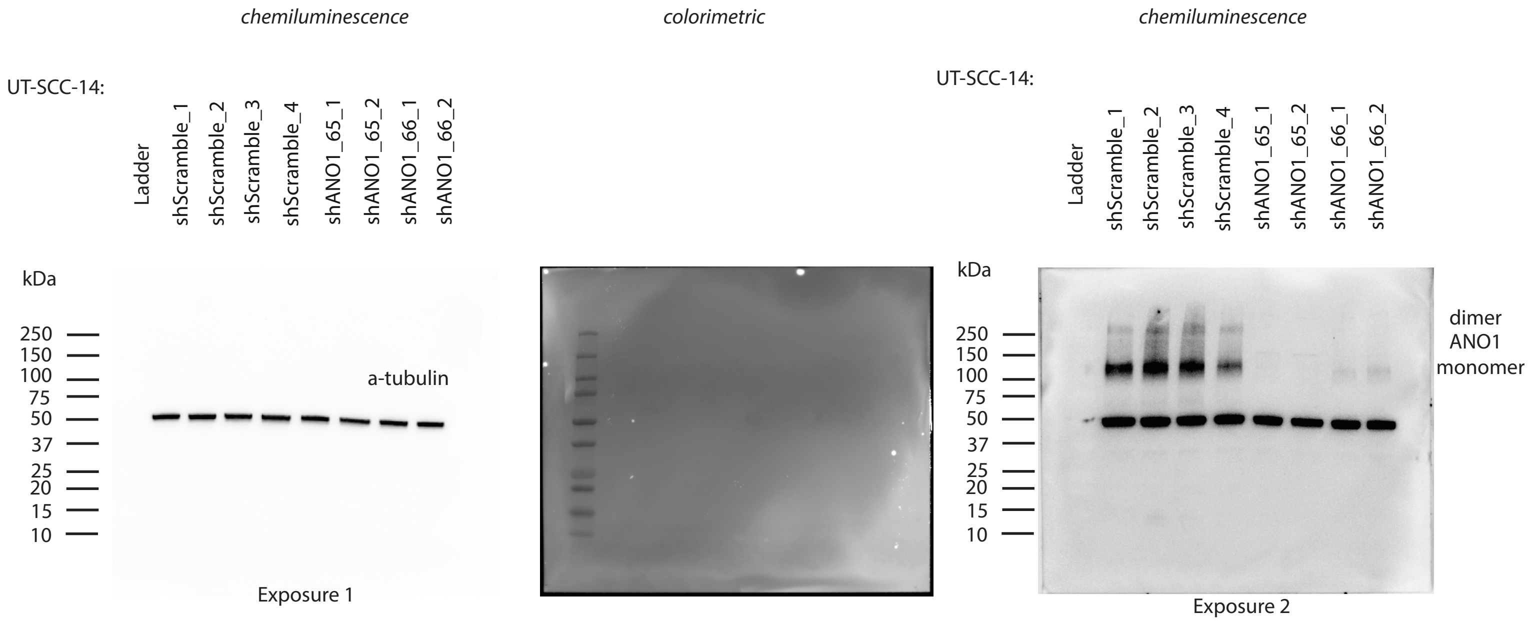

	Intensity of each band (background adjusted)							
	UT-SCC-14 shScramble-1	UT-SCC-14 shScramble-2	UT-SCC-14 shScramble-3	UT-SCC-14 shScramble-4	UT-SCC-14 shANO1_65-1	UT-SCC-14 shANO1_65-2	UT-SCC-14 shANO1_66-1	UT-SCC-14 shANO1_66-2
ANO1	5614103	6002751	5212174	3357209	89603	12904	298802	512510
α-tubulin	18575333	16319536	13843395	14311993	12855591	10660942	11556502	10008511
Intensity ratio	0.30	0.37	0.38	0.23	0.01	0.00	0.03	0.05

Figure S7 Uncropped blots of Figure S3A.
Immunoblot analysis of changes in proteins ANO1, COXIV and a-tubulin in cytoplasmic and mitochondrial fractions of the cell line UT-SCC-8. Densitometry readings and intensity ratios (normalized values) are presented in the table under the whole blots.

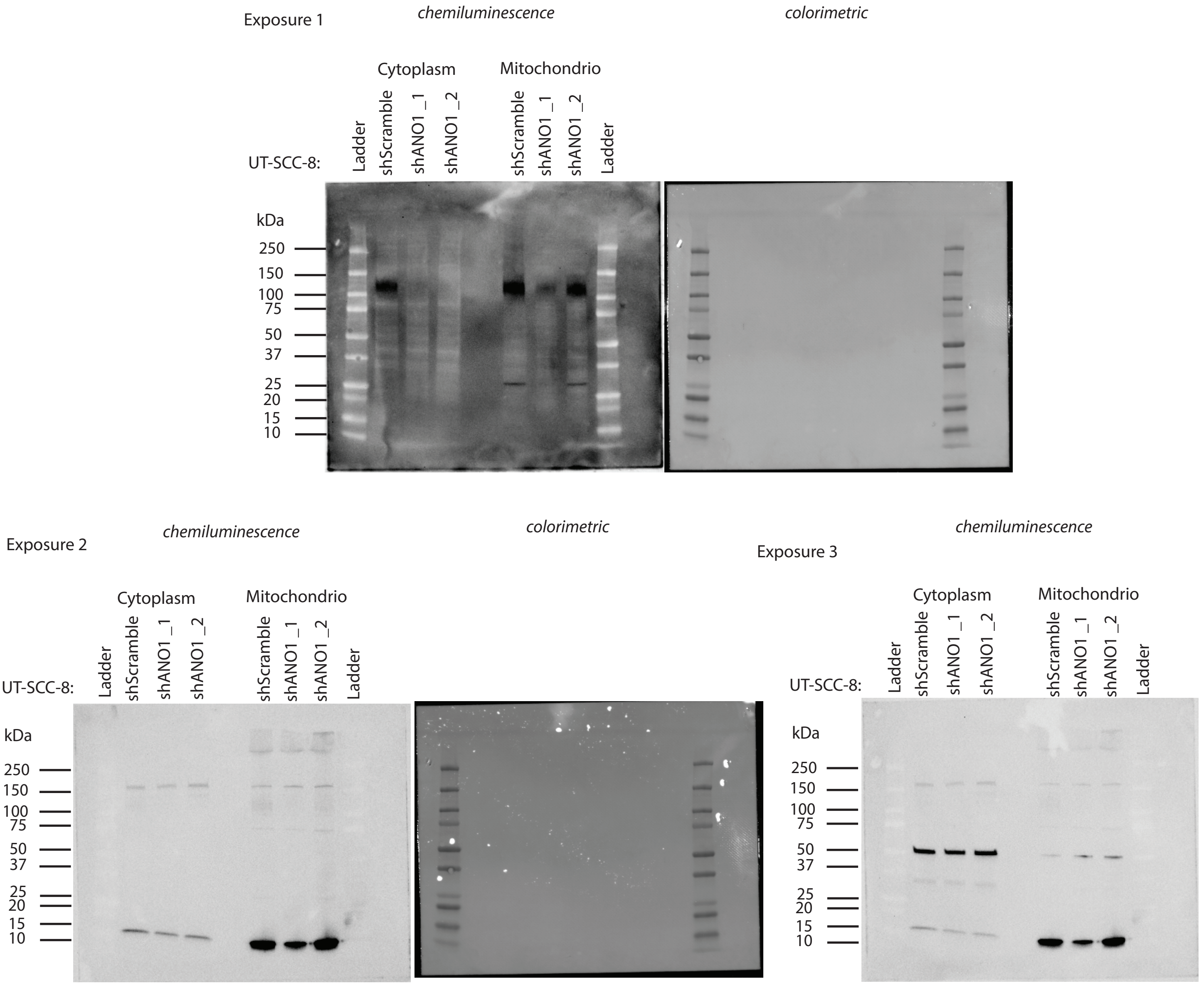

	Intensity of each band (background adjusted)					
	UT-SCC-8 shScramble cytoplasmic	UT-SCC-8 shANO1_1 cytoplasmic	UT-SCC-8 shANO1_2 cytoplasmic	UT-SCC-8 shScramble mitochondrio	UT-SCC-8 shANO1_1 mitochondrio	UT-SCC-8 shANO1_2 mitochondrio
ANO1	1708467	142562	10719	2423751	789525	1635683
a-tubulin	848404	684063	1013428	59593	136652	151321
COXIV	251119	156889	205156	2307407	1123599	2702501
Intensity ratio (to a-tubulin)	2.01	0.21	0.01	40.67	5.78	10.81
Intensity ratio (to COXIV)	6.80	0.91	0.05	1.05	0.70	0.61

Figure S7 Uncropped blots of Figure S3B.
Immunoblot analysis of the proteins COXIV and a-tubulin from co-immunoprecipitated samples with ANO1 antibody, as well as their corresponding inputs samples. Cell lines utilized were UT-SCC-8 and UT-SCC-14. COXIV antibody used was raised in mouse (Abcam).

colorimetric

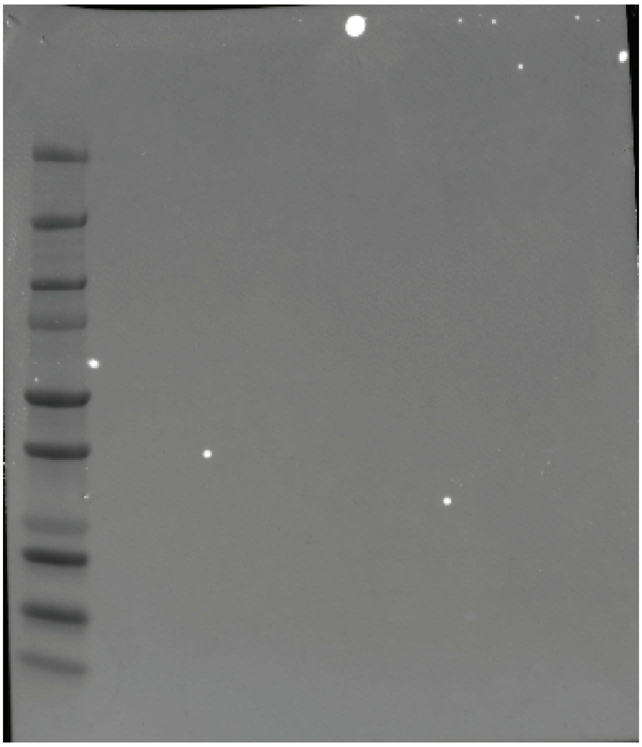

chemiluminescence

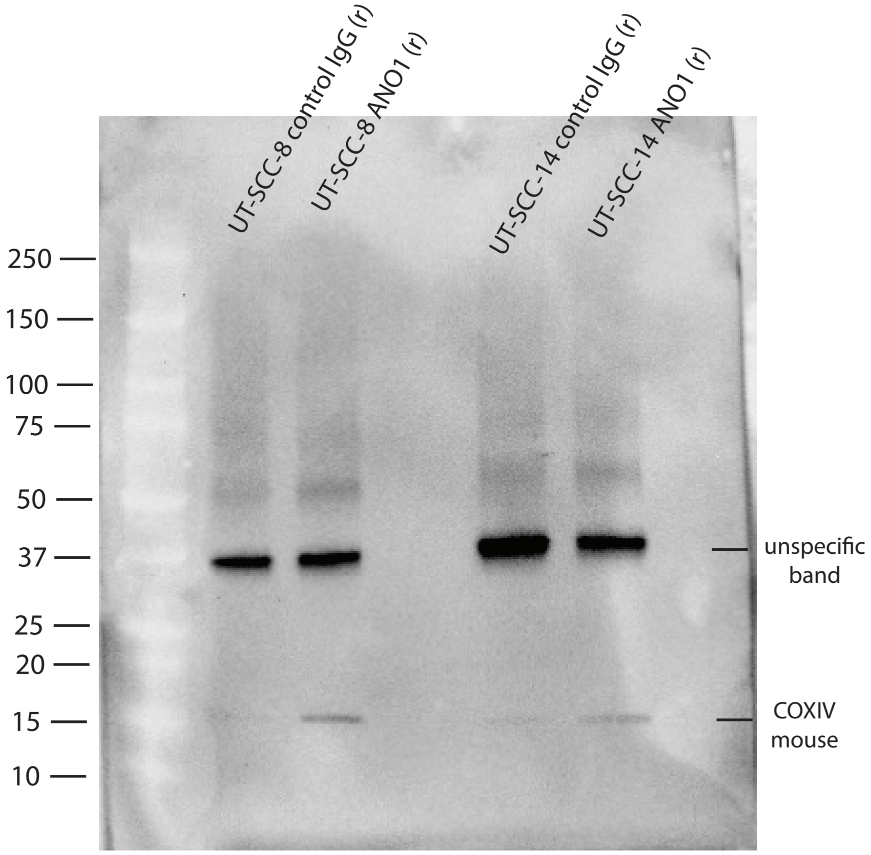

chemiluminescence

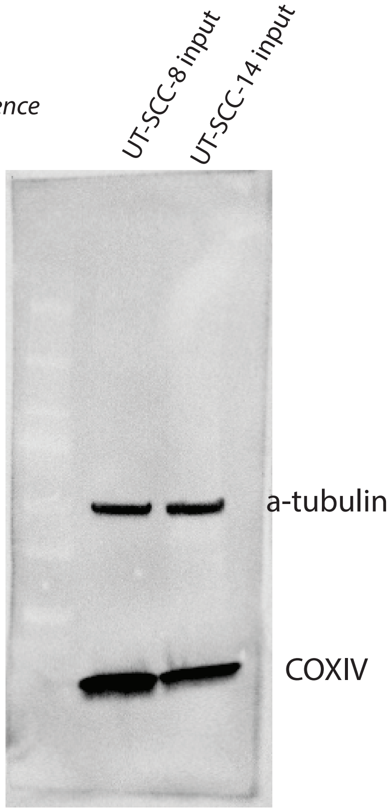

colorimetric

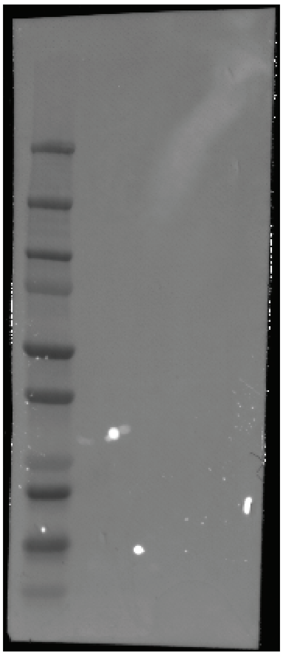

Figure S7 Uncropped blots of Figure S3C.
Immunoblot analysis of the proteins ANO1, MCL1, BCL-XL, BCL2, BIM and a-tubulin from cell lysates of a panel of UTSCC cell lines. Alpha-tubulin was used as the loading control to normalize the quantities of the proteins. Densitometry readings and normalized values are presented in the tables under the respective blots.

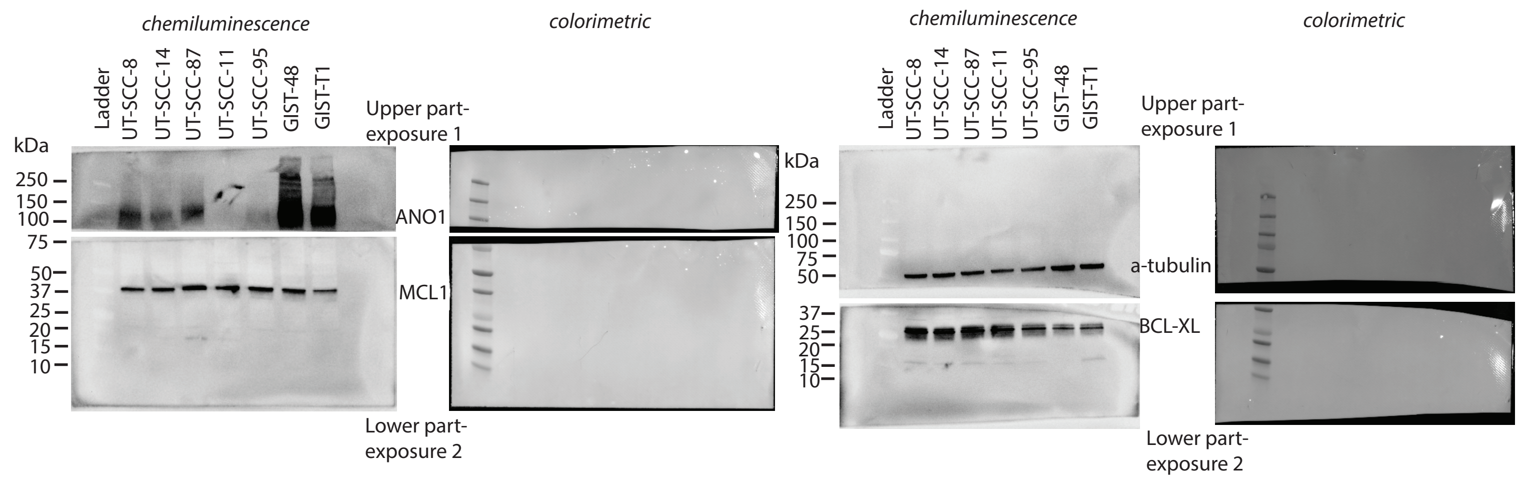

The two gels above were run simulteneously in the sds-page casette, after which the two mini-gels were transfered in parallel using a midi-PVDF membrane.

	Intensity of each band (background adjusted)						
	UT-SCC-8	UT-SCC-14	UT-SCC-87	UT-SCC-11	UT-SCC-95	GIST-48	GIST-T1
ANO1	1557644	1087278	1139776	370644	677492	5937297	4030323
MCL1	1096911	1227031	1657176	1711423	1600784	1355426	779095
BCL-XL	4124391	3602105	3908531	3812586	2906652	1824880	2112793
α-tubulin	1273000	1329923	1128754	840036	907972	1754374	2047973
Intensity ratio (ANO1/a-tubulin)	1.22	0.82	1.01	0.44	0.75	3.38	1.97
Intensity ratio (MCL1/a-tubulin)	0.86	0.92	1.47	2.04	1.76	0.77	0.38
Intensity ratio (BCL-XL/a-tubulin)	3.24	2.71	3.46	4.54	3.20	1.04	1.03

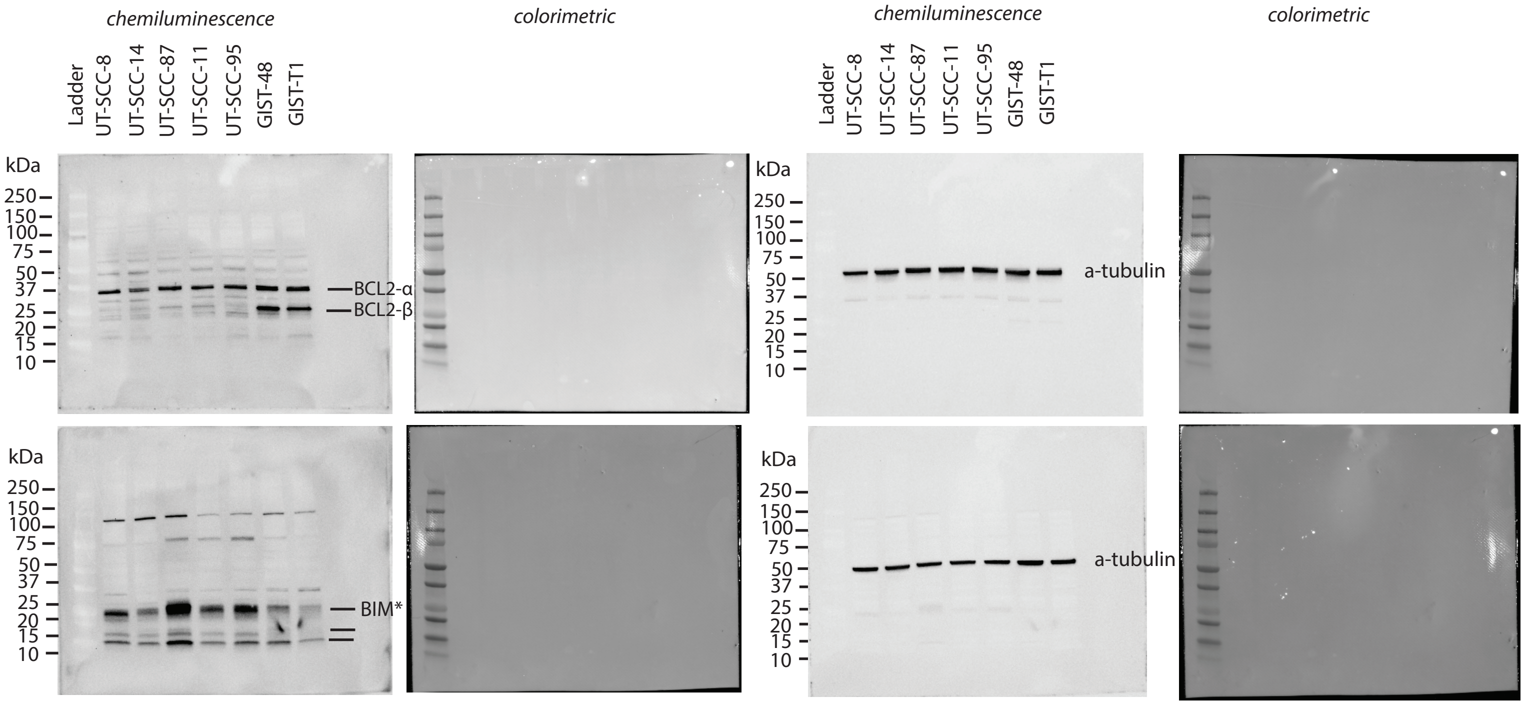

*BIM-specific bands: 23, 16, 12 kDa. Dnsitometry was measured for the main, 23 kD band.

	Intensity of each band (background adjusted)						
	UT-SCC-8	UT-SCC-14	UT-SCC-87	UT-SCC-11	UT-SCC-95	GIST-48	GIST-T1
BCL2-α	1005709	597373	1204262	974539	1113073	1544510	1455693
BCL2-β	66430	80284	183876	158980	69084	1530632	1110605
a-tubulin	1562656	1807257	1711540	1610574	1709107	1913249	2154470
Intensity ratio (BCL2-α /a-tubulin)	0.64	0.33	0.70	0.61	0.65	0.81	0.68
Intensity ratio (BCL2-β/a-tubulin)	0.04	0.04	0.11	0.10	0.04	0.80	0.52
BIM	1556109	791182	4124364	1519685	2188691	871609	360657
α-tubulin	1389433	1154068	1109790	1079964	1333818	1929931	1860979
Intensity ratio (BIM/a-tubulin)	1.12	0.69	3.72	1.41	1.64	0.45	0.19

Figure S7 Uncropped blots of Figure S4B.

Immunoblot analysis of the proteins ANO1 and a-tubulin from cell lysates of UT-SCC-8 and UT-SCC-14 treated with a range of concentrations of Ani9 inhibitor. Alpha-tubulin was used as the control to normalize the quantities of the proteins. Densitometry readings and normalized values are presented in the tables under the respective blots.

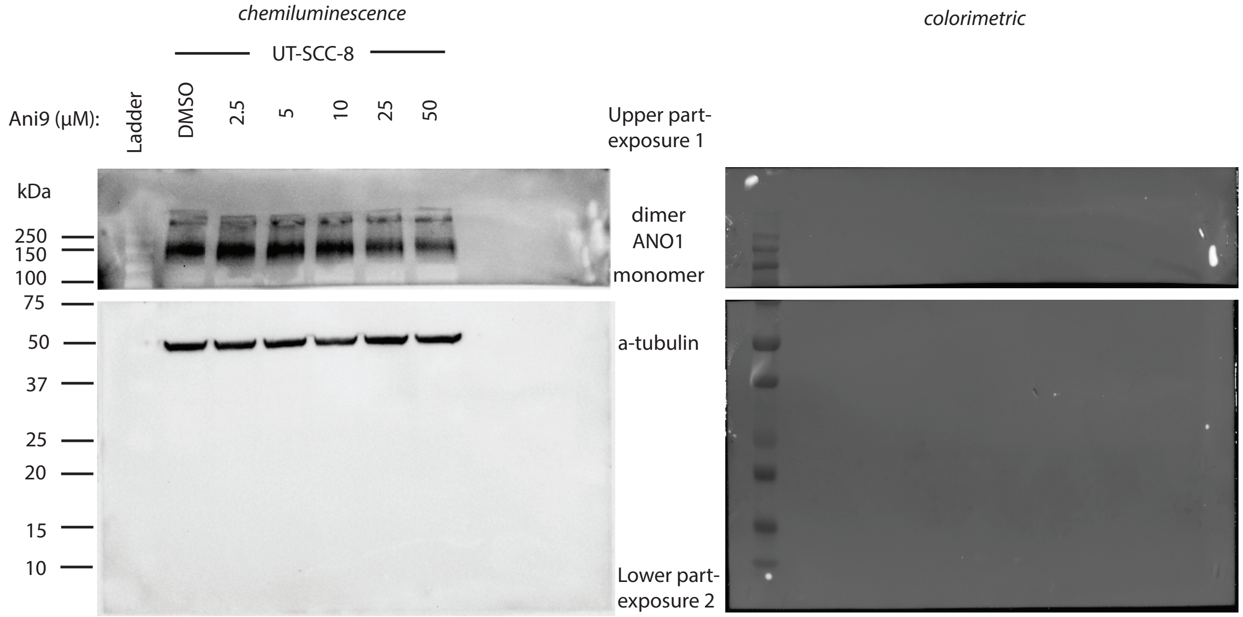

	Intensity of each band (background adjusted)					
	UT-SCC-8 DMSO	UT-SCC-8 2.5 µM Ani9	UT-SCC-8 5 µM Ani9	UT-SCC-8 10 µM Ani9	UT-SCC-8 25 µM Ani9	UT-SCC-8 50 µM Ani9
ANO1 (monomer)	3076860	3389209	3453302	3385514	2884734	2801066
a-tubulin	2800540	2347174	2492055	1983652	3086486	2774190
Intensity ratio (to a-tubulin)	1.10	1.44	1.39	1.71	0.93	1.01

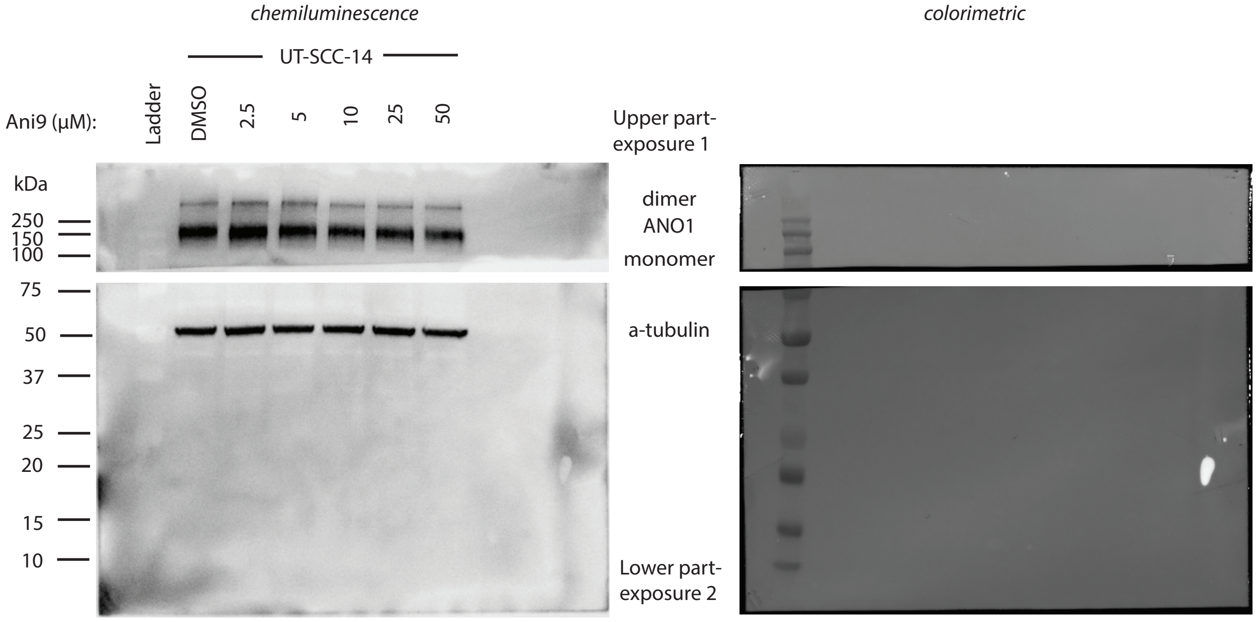

	Intensity of each band (background adjusted)					
	UT-SCC-14 DMSO	UT-SCC-14 2.5 µM Ani9	UT-SCC-14 5 µM Ani9	UT-SCC-14 10 µM Ani9	UT-SCC-14 25 µM Ani9	UT-SCC-14 50 µM Ani9
ANO1 (monomer)	3120427	3491869	2990948	2697955	2776385	2757160
a-tubulin	2231352	2400024	2053325	2280005	2420889	2233379
Intensity ratio (to a-tubulin)	1.40	1.45	1.46	1.18	1.15	1.23

Figure S7 Uncropped blots of Figure S6B.
Immunoblot analysis of the proteins ANO1 and a-tubulin of cell lysates from GIST-48 and GIST-T1 treated with a range of concentrations of Ani9-5f inhibitor. Alpha-tubulin was used as the control to normalize the quantities of the proteins. Densitometry readings and normalized values are presented in the tables under the respective blots.

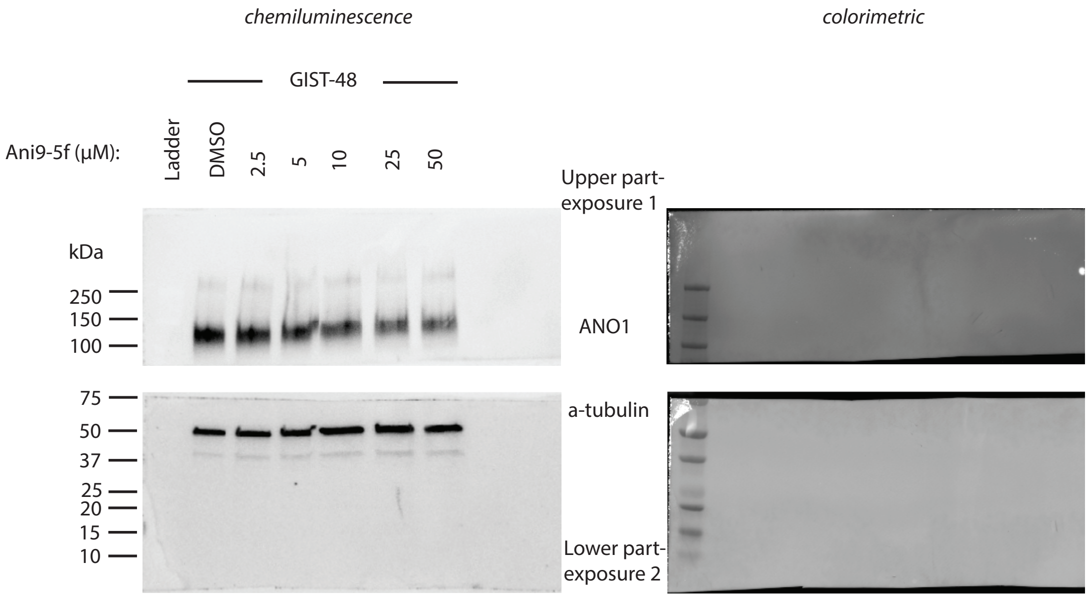

	Intensity of each band (background adjusted)					
	GIST-48 DMSO	GIST-48 2.5 µM Ani9-5f	GIST-48 5 µM Ani9-5f	GIST-48 10 µM Ani9-5f	GIST-48 25 µM Ani9-5f	GIST-48 50 µM Ani9-5f
ANO1 (monomer)	4103574	4148841	3423658	2861404	2992919	2876292
a-tubulin	1403925	1682958	1553548	2096217	2379447	1887867
Intensity ratio (to a-tubulin)	2.92	2.47	2.20	1.37	1.26	1.52

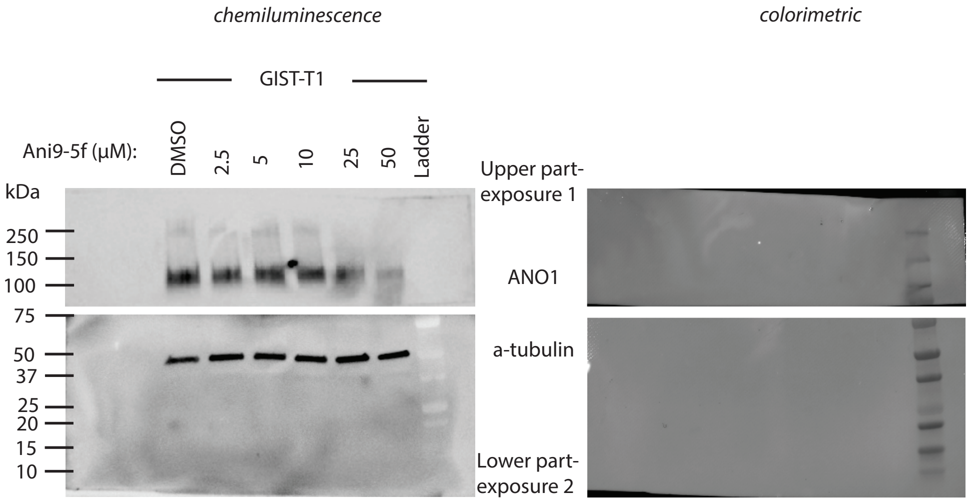

	Intensity of each band (background adjusted)					
	GIST-T1 DMSO	GIST-T1 2.5 µM Ani9-5f	GIST-T1 5 µM Ani9-5f	GIST-T1 10 µM Ani9-5f	GIST-T1 25 µM Ani9-5f	GIST-T1 50 µM Ani9-5f
ANO1 (monomer)	1409509	1089275	866654	343499	729478	384773
a-tubulin	1095759	1701096	1548198	1382456	1756769	1595450
Intensity ratio (to a-tubulin)	1.29	0.64	0.56	0.25	0.42	0.24

Figure S8

Run 1-UT-SCC-8

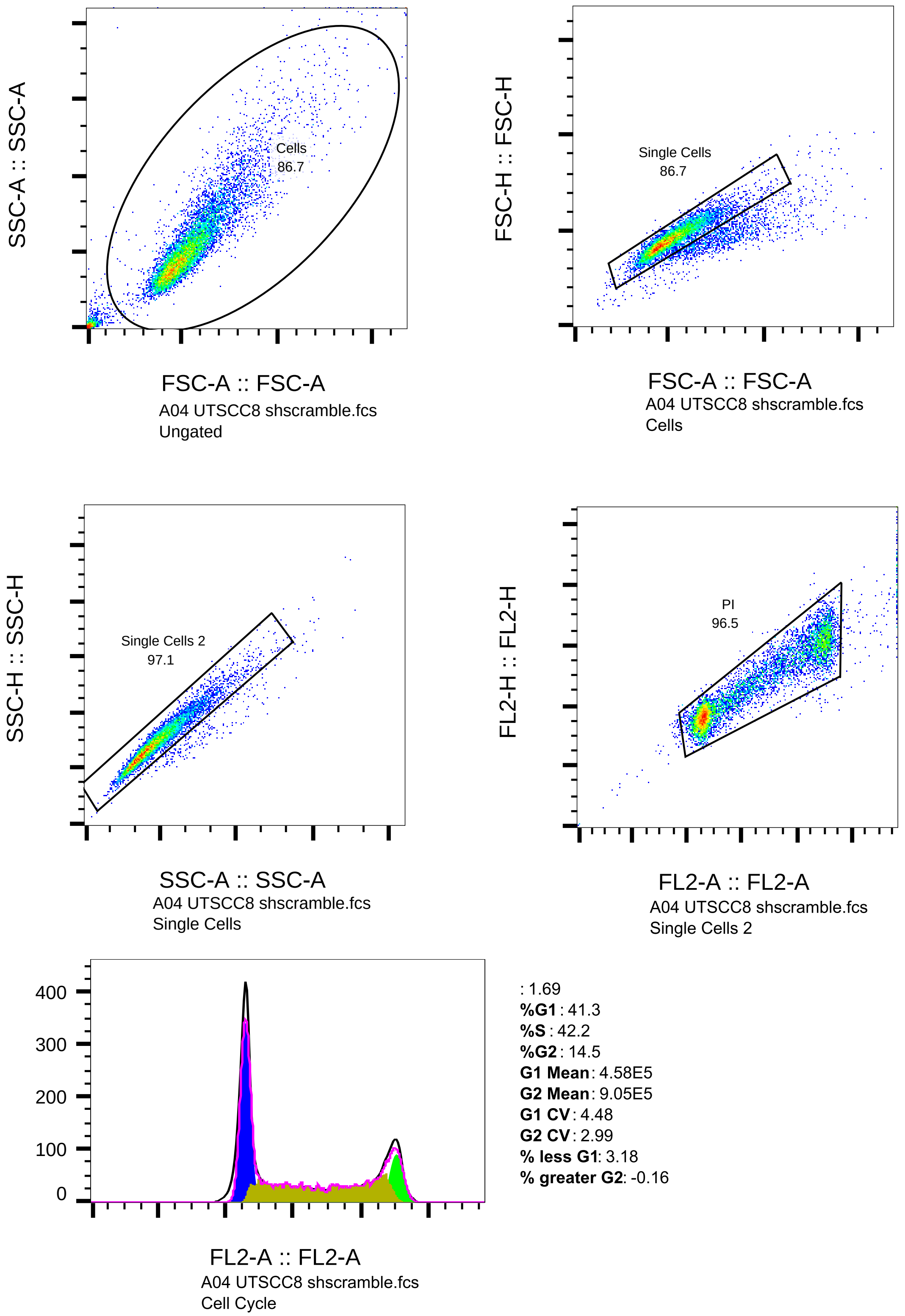

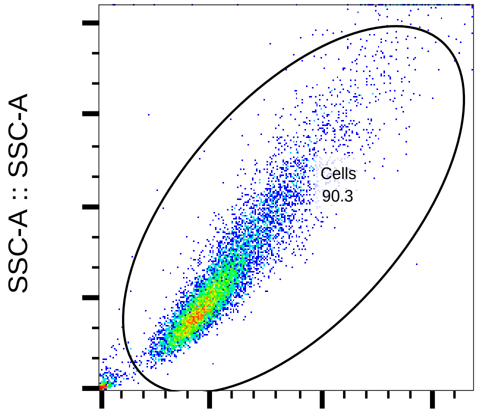

FSC-A :: FSC-A
A05 UTSCC8 shANO1 65.fcs
Ungated

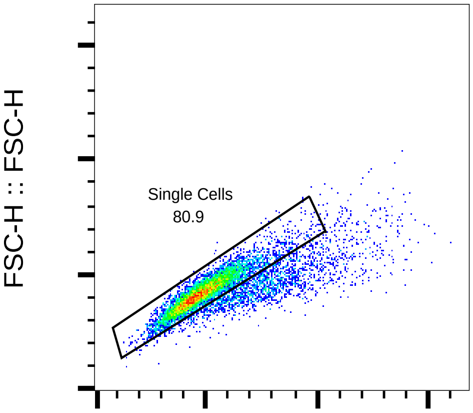

FSC-A :: FSC-A
A05 UTSCC8 shANO1 65.fcs
Cells

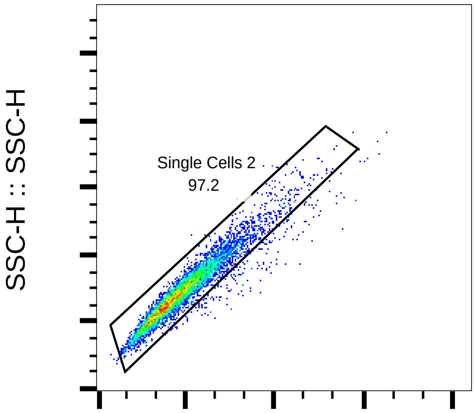

SSC-A :: SSC-A
A05 UTSCC8 shANO1 65.fcs
Single Cells

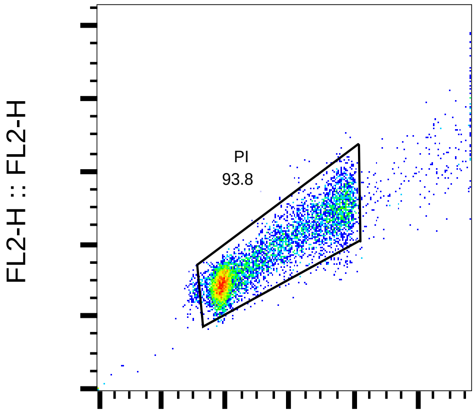

FL2-A :: FL2-A
A05 UTSCC8 shANO1 65.fcs
Single Cells 2

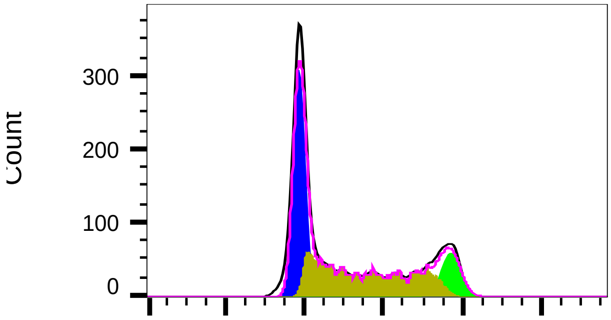

: 1.02
%G1 : 43.8
%S : 41.6
%G2 : 13.4
G1 Mean : 386630
G2 Mean : 7.71E5
G1 CV : 5.71
G2 CV : 4.56
% less G1: 2.19
% greater G2: -0.87

FL2-A :: FL2-A
A05 UTSCC8 shANO1 65.fcs
Cell Cycle

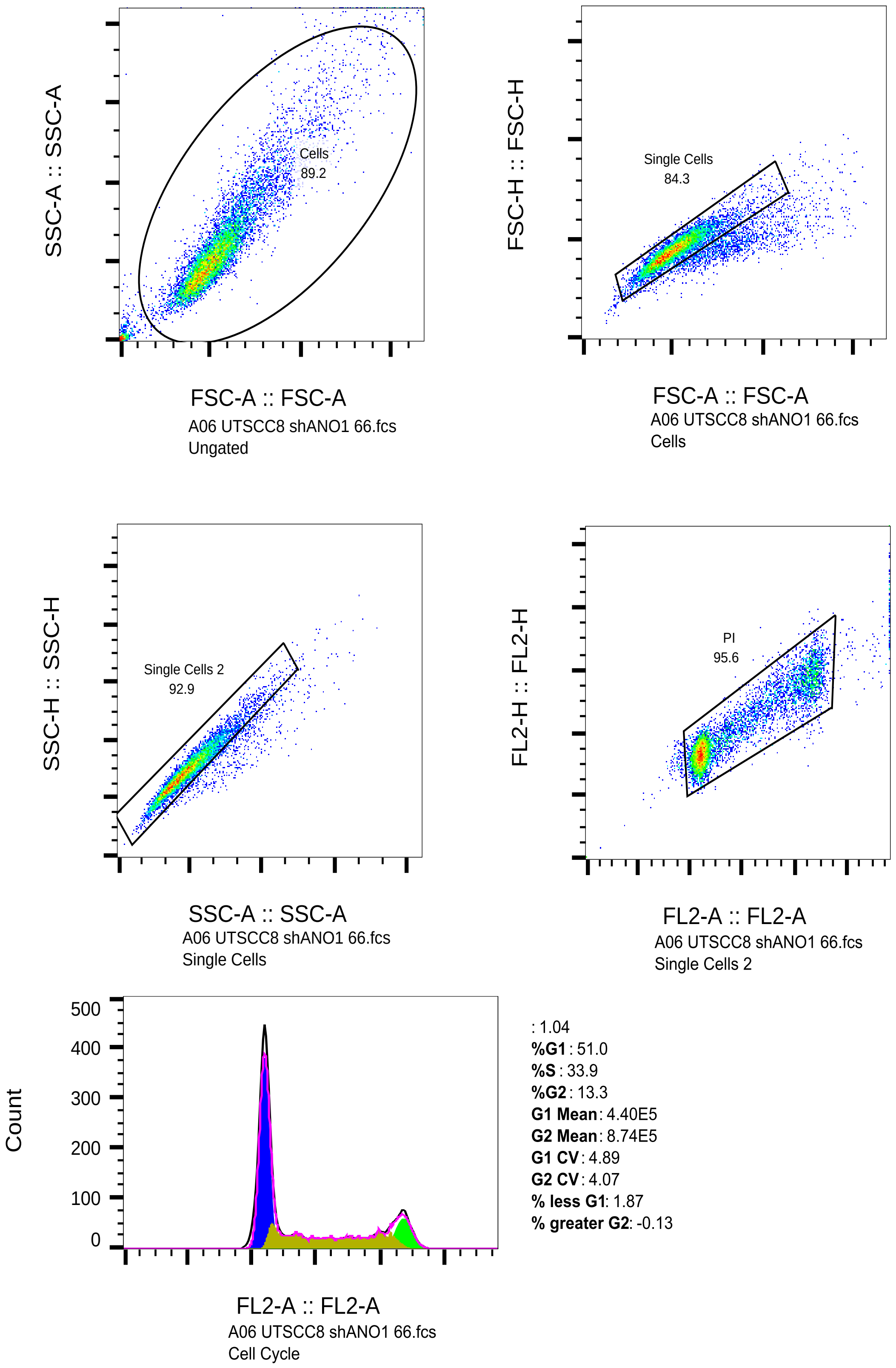
SSC-A :: SSC-A
Cells
89.2
FSC-A :: FSC-A
A06 UTSCC8 shANO1 66.fcs
Ungated
FSC-H :: FSC-H
Single Cells
84.3
FSC-A :: FSC-A
A06 UTSCC8 shANO1 66.fcs
Cells
SSC-H :: SSC-H
Single Cells 2
92.9
SSC-A :: SSC-A
A06 UTSCC8 shANO1 66.fcs
Single Cells
FL2-H :: FL2-H
PI
95.6
FL2-A :: FL2-A
A06 UTSCC8 shANO1 66.fcs
Single Cells 2
Count
500
400
300
200
100
0
FL2-A :: FL2-A
A06 UTSCC8 shANO1 66.fcs
Cell Cycle
: 1.04
%G1 : 51.0
%S : 33.9
%G2 : 13.3
G1 Mean : 4.40E5
G2 Mean : 8.74E5
G1 CV : 4.89
G2 CV : 4.07
% less G1: 1.87
% greater G2: -0.13

Run 2- UT-SCC-8

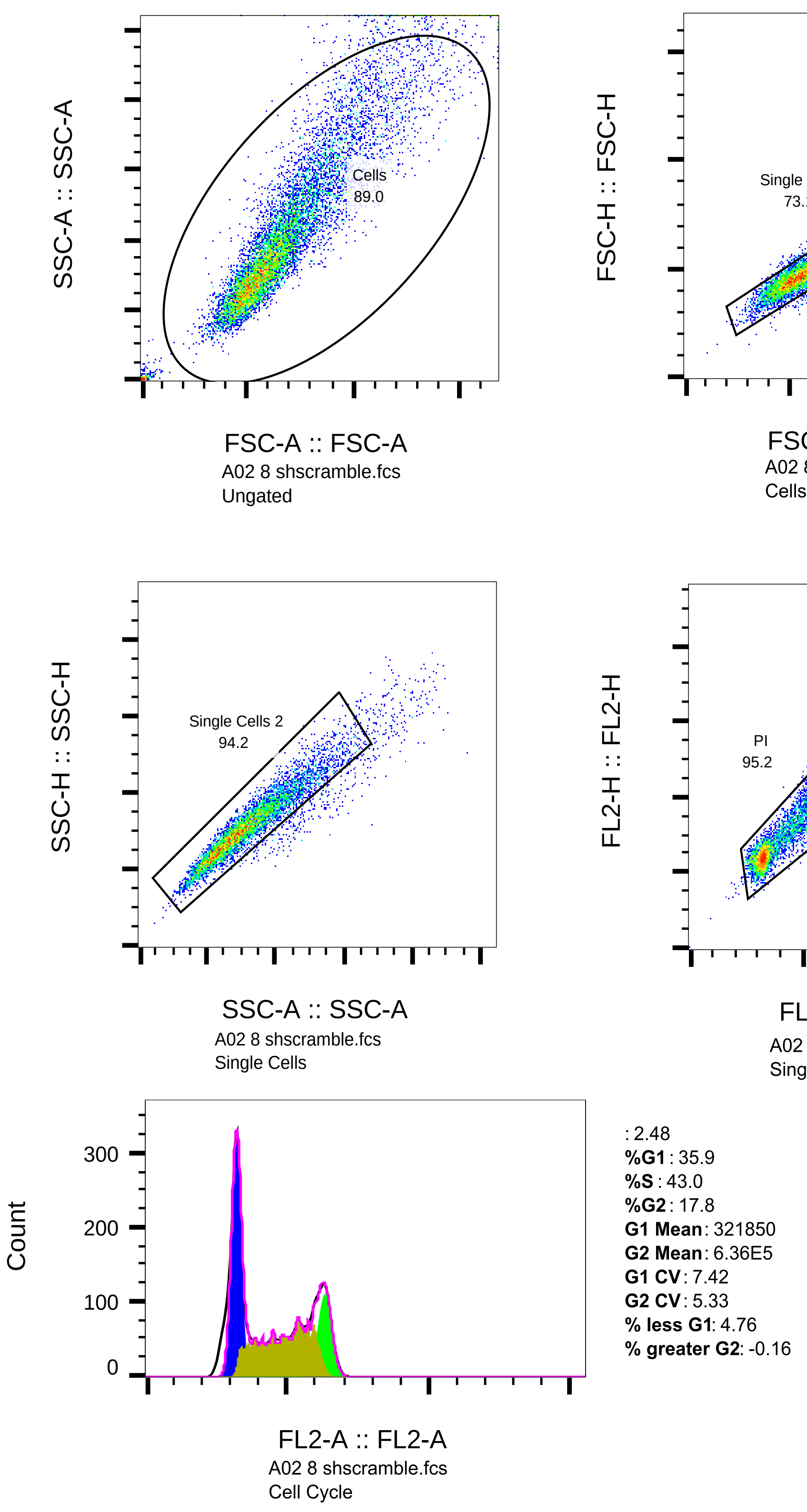
SSC-A :: SSC-A
Cells
89.0
FSC-A :: FSC-A
A02 8 shscramble.fcs
Ungated
FSC-H :: FSC-H
Single Cells
73.1
FSC-A :: FSC-A
A02 8 shscramble.fcs
Cells
SSC-H :: SSC-H
Single Cells 2
94.2
SSC-A :: SSC-A
A02 8 shscramble.fcs
Single Cells
FL2-H :: FL2-H
PI
95.2
FL2-A :: FL2-A
A02 8 shscramble.fcs
Single Cells 2
Count
300
200
100
0
FL2-A :: FL2-A
A02 8 shscramble.fcs
Cell Cycle
: 2.48
%G1 : 35.9
%S : 43.0
%G2 : 17.8
G1 Mean : 321850
G2 Mean : 6.36E5
G1 CV : 7.42
G2 CV : 5.33
% less G1: 4.76
% greater G2: -0.16

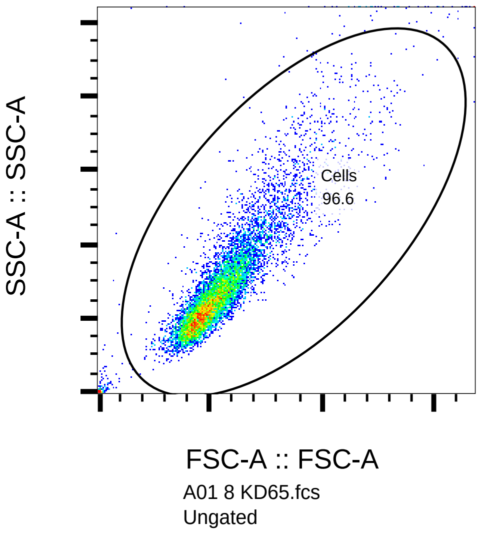

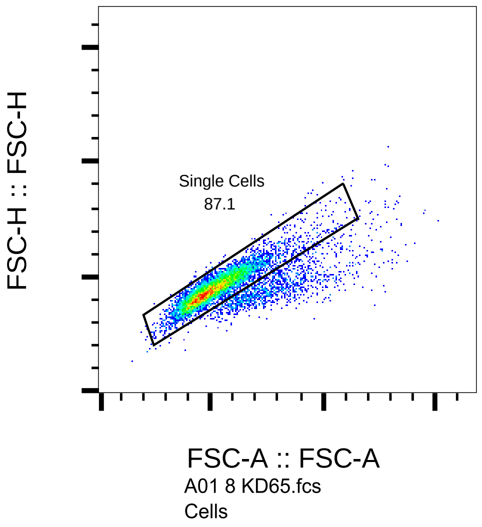

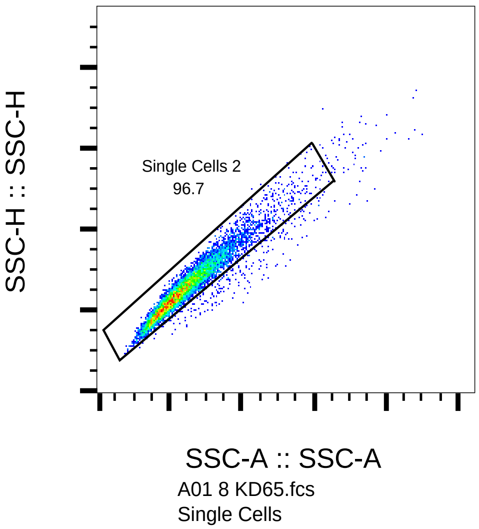

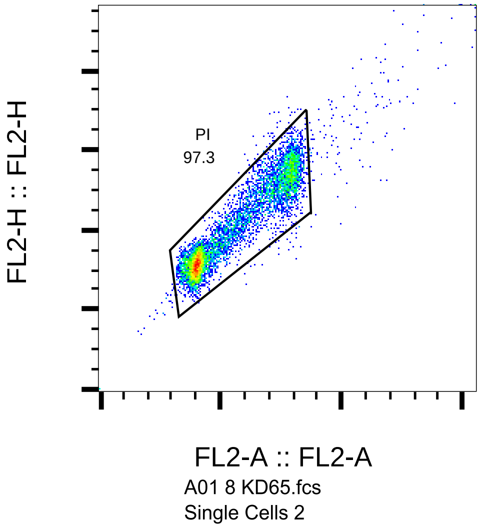

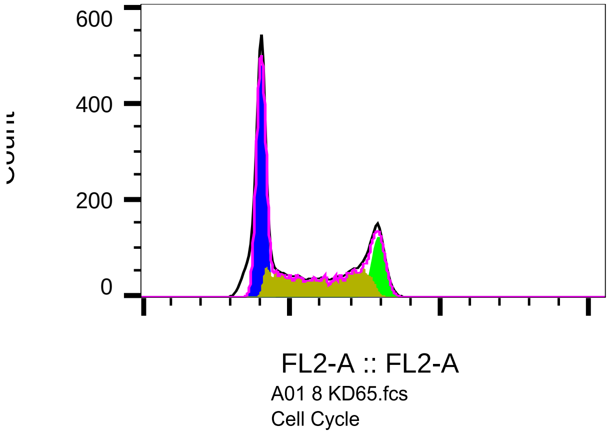

: 2.84
%G1 : 41.6
%S : 37.4
%G2 : 15.9
G1 Mean : 4.03E5
G2 Mean : 7.95E5
G1 CV : 5.15
G2 CV : 3.91
% less G1: 5.49
% greater G2: 0.21

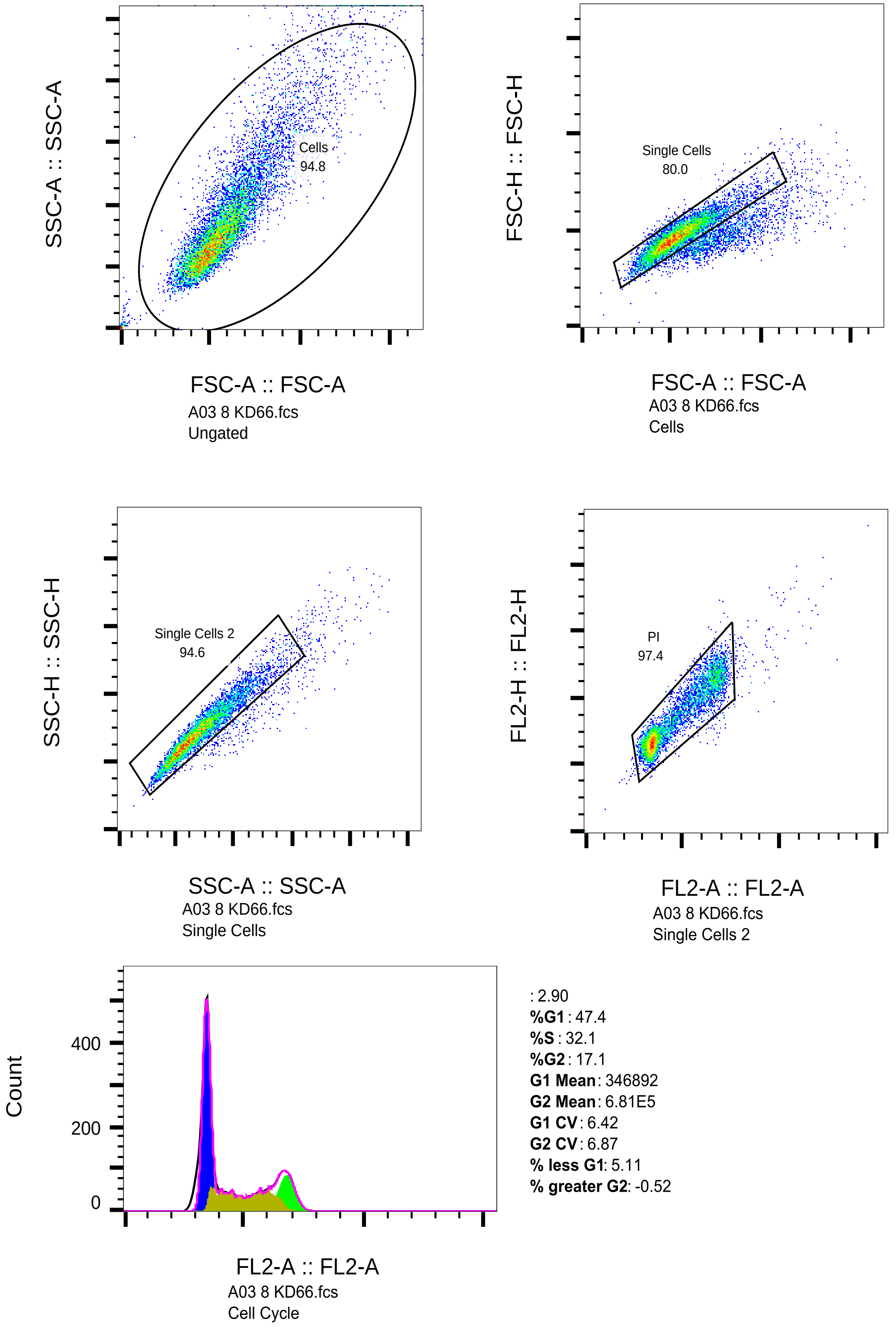

SSC-A :: SSC-A
Cells
94.8
FSC-A :: FSC-A
A03 8 KD66.fcs
Ungated
FSC-H :: FSC-H
Single Cells
80.0
FSC-A :: FSC-A
A03 8 KD66.fcs
Cells
SSC-H :: SSC-H
Single Cells 2
94.6
SSC-A :: SSC-A
A03 8 KD66.fcs
Single Cells
FL2-H :: FL2-H
PI
97.4
FL2-A :: FL2-A
A03 8 KD66.fcs
Single Cells 2
Count
400
200
0
FL2-A :: FL2-A
A03 8 KD66.fcs
Cell Cycle
: 2.90
%G1 : 47.4
%S : 32.1
%G2 : 17.1
G1 Mean : 346892
G2 Mean : 6.81E5
G1 CV : 6.42
G2 CV : 6.87
% less G1: 5.11
% greater G2: -0.52

Run 1- UT-SCC-14

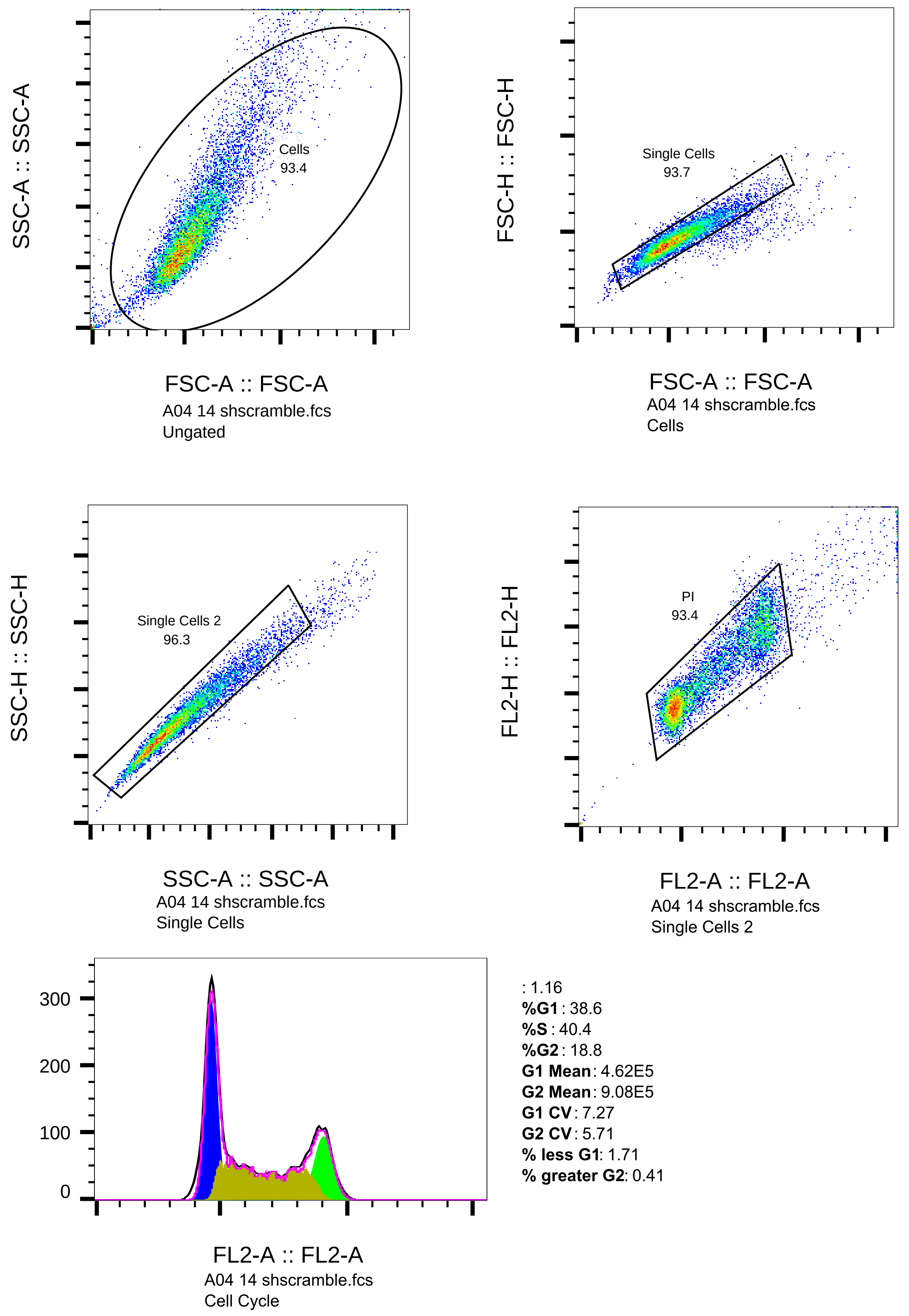

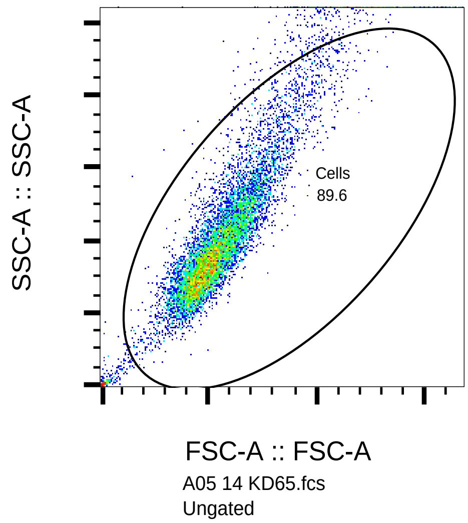

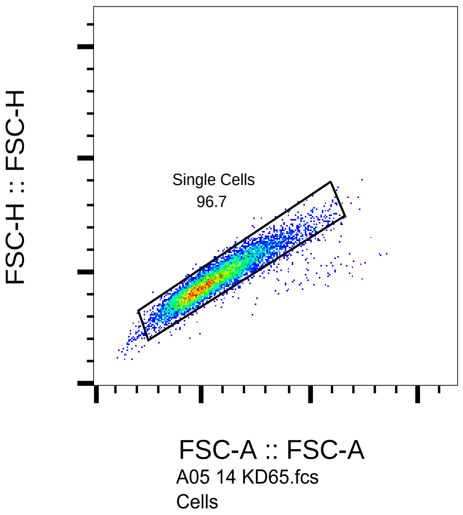

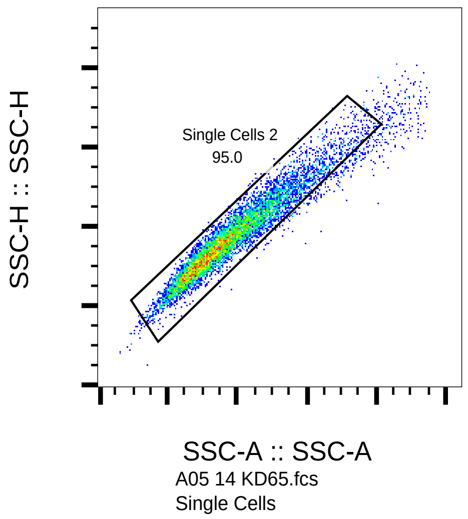

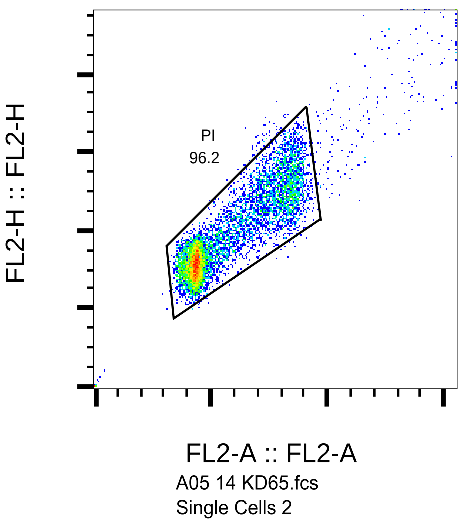

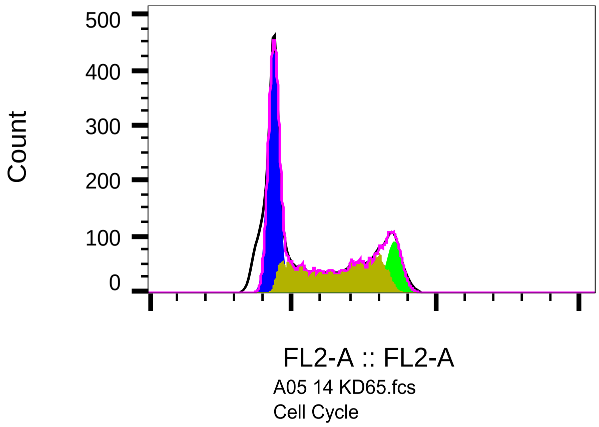

: 4.12
%G1 : 41.5
%S : 38.9
%G2 : 12.3
G1 Mean : 4.37E5
G2 Mean : 8.57E5
G1 CV : 6.02
G2 CV : 4.38
% less G1: 7.35
% greater G2: 0.58

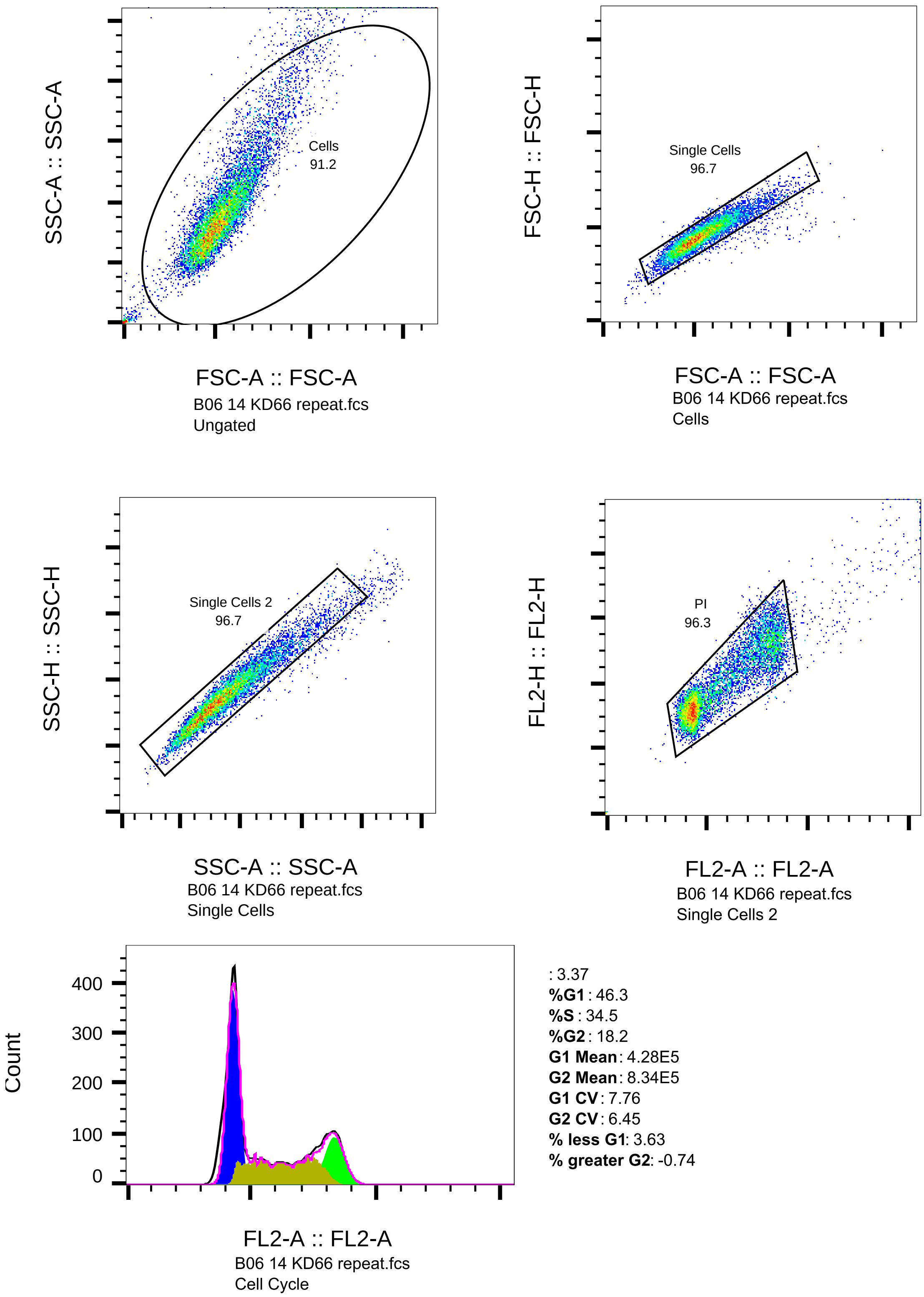

SSC-A :: SSC-A
Cells
91.2
FSC-A :: FSC-A
B06 14 KD66 repeat.fcs
Ungated
FSC-H :: FSC-H
Single Cells
96.7
FSC-A :: FSC-A
B06 14 KD66 repeat.fcs
Cells
SSC-H :: SSC-H
Single Cells 2
96.7
SSC-A :: SSC-A
B06 14 KD66 repeat.fcs
Single Cells
FL2-H :: FL2-H
PI
96.3
FL2-A :: FL2-A
B06 14 KD66 repeat.fcs
Single Cells 2
Count
400
300
200
100
0
FL2-A :: FL2-A
B06 14 KD66 repeat.fcs
Cell Cycle
: 3.37
%G1 : 46.3
%S : 34.5
%G2 : 18.2
G1 Mean : 4.28E5
G2 Mean : 8.34E5
G1 CV : 7.76
G2 CV : 6.45
% less G1: 3.63
% greater G2: -0.74

Run 2- UT-SCC-14

B04 UT-SCC-14 shScramble -2.fcs
Cell Cycle
10952

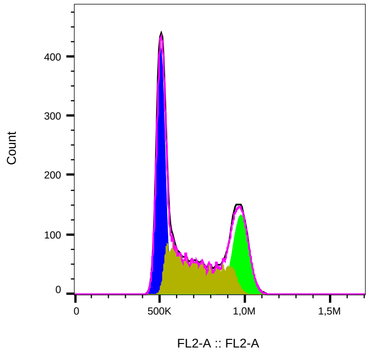

: 1.08
%G1 : 39.6
%S : 38.3
%G2 : 23.1
G1 Mean : 5.08E5
G2 Mean : 9.78E5
G1 CV : 6.94
G2 CV : 6.61
% less G1 : -0.73
% greater G2 : -0.026

B05 UT-SCC-14 ANO1 KD-1 -2.fcs
Cell Cycle
11099

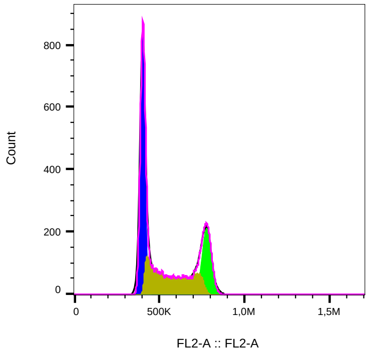

: 0.86
%G1 : 43.5
%S : 34.9
%G2 : 20.2
G1 Mean : 4.05E5
G2 Mean : 7.81E5
G1 CV : 4.97
G2 CV : 4.83
% less G1 : 0.41
% greater G2 : 0.69

B06 UT-SCC-14 ANO1 KD-2 -2.fcs
Cell Cycle
9405

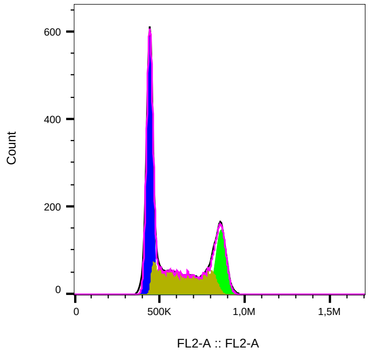

: 1.38
%G1 : 44.7
%S : 34.7
%G2 : 19.4
G1 Mean : 4.45E5
G2 Mean : 8.63E5
G1 CV : 5.45
G2 CV : 4.84
% less G1 : 1.18
% greater G2 : 0.32

Run 3- UT-SCC-8

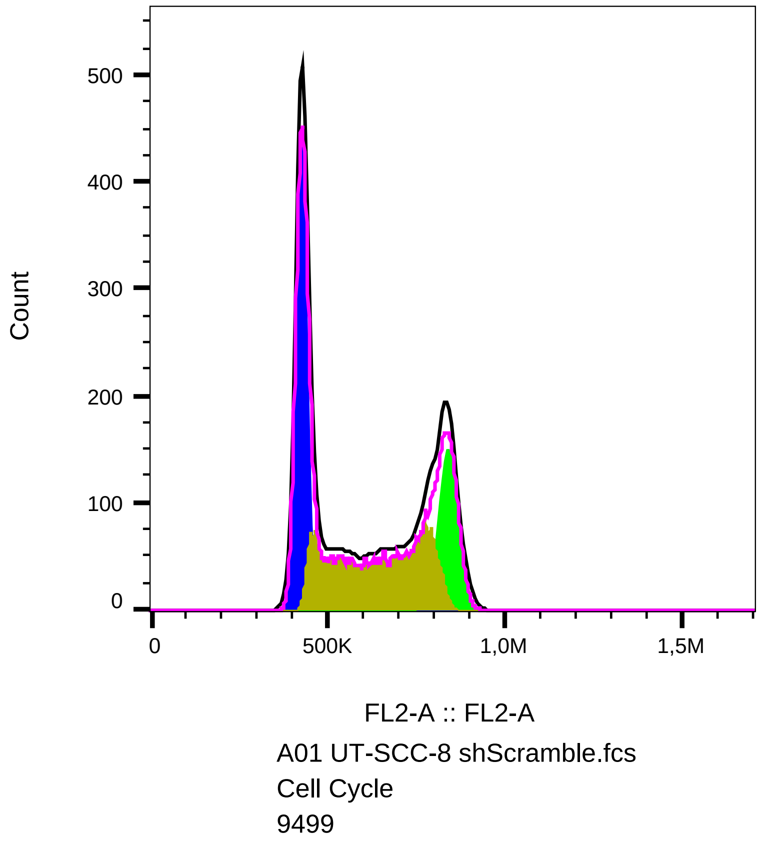

: 0,92
%G1 : 35,0
%S : 43,0
%G2 : 20,3
G1 Mean : 4,29E5
G2 Mean : 8,43E5
G1 CV : 5,30
G2 CV : 4,55
% less G1 : 0,13
% greater G2 : 0,91

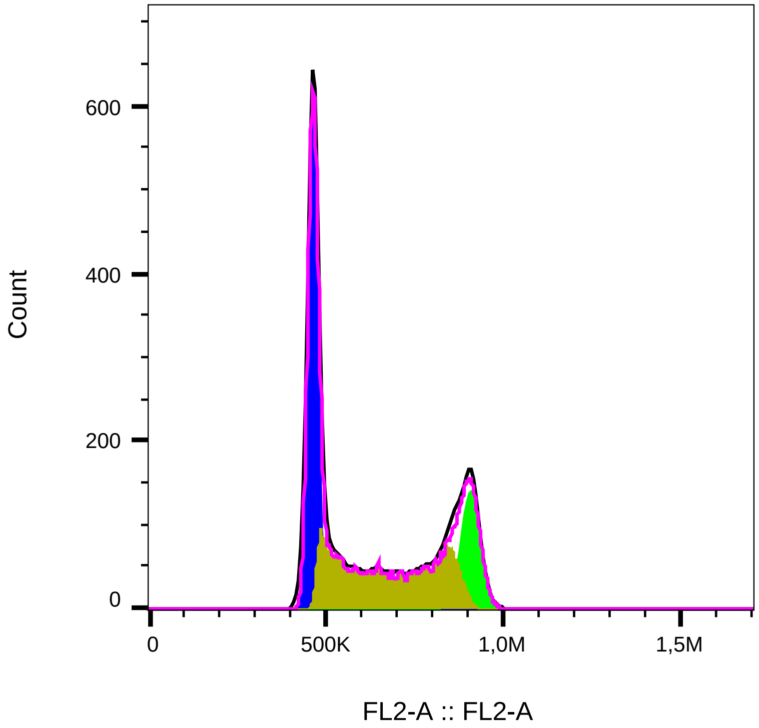

A02 UT-SCC-8 ANO1 KD-1.fcs
Cell Cycle
9418

: 1,17
%G1 : 39,1
%S : 42,0
%G2 : 18,0
G1 Mean : 4,66E5
G2 Mean : 9,10E5
G1 CV : 4,26
G2 CV : 4,29
% less G1 : 0,90
% greater G2 : -0,19

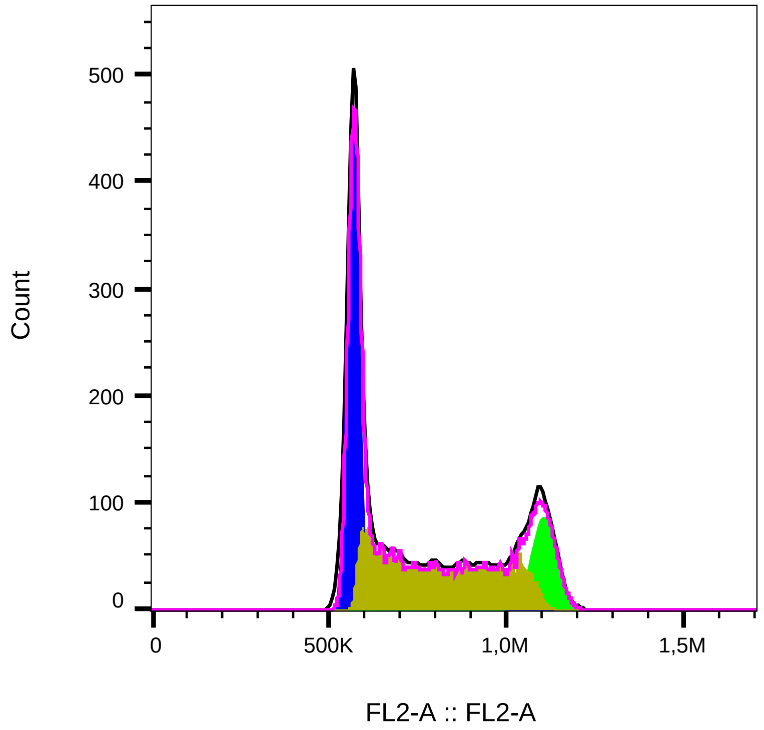

: 1,21
%G1 : 37,9
%S : 46,2
%G2 : 15,1
G1 Mean : 5,72E5
G2 Mean : 1,11E6
G1 CV : 4,19
G2 CV : 4,48
% less G1 : 0,99
% greater G2 : -0,028

A06 UT-SCC-8 ANO1 KD2- 2.fcs
Cell Cycle
8940

Run 3- UT-SCC-14

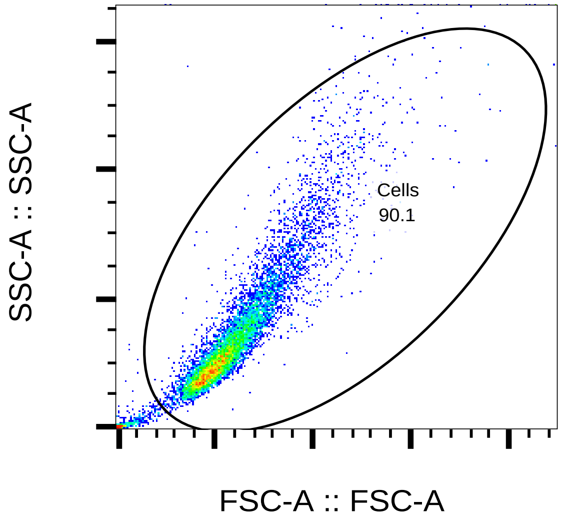

A04 14 shscramble.fcs
Ungated

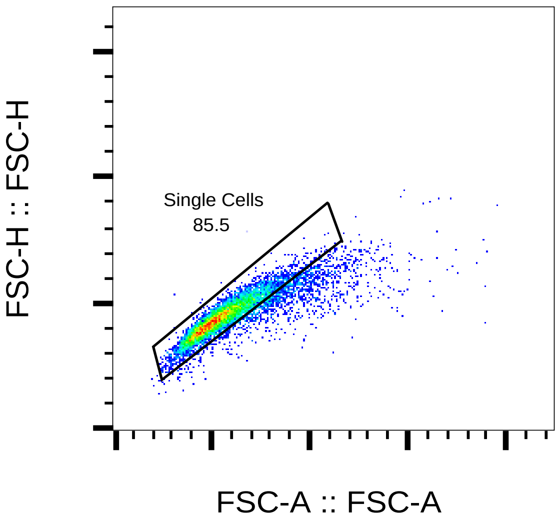

A04 14 shscramble.fcs
Cells

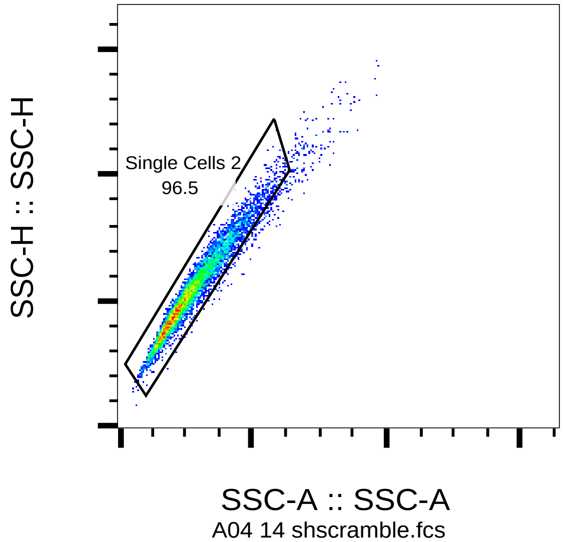

A04 14 shscramble.fcs
Single Cells

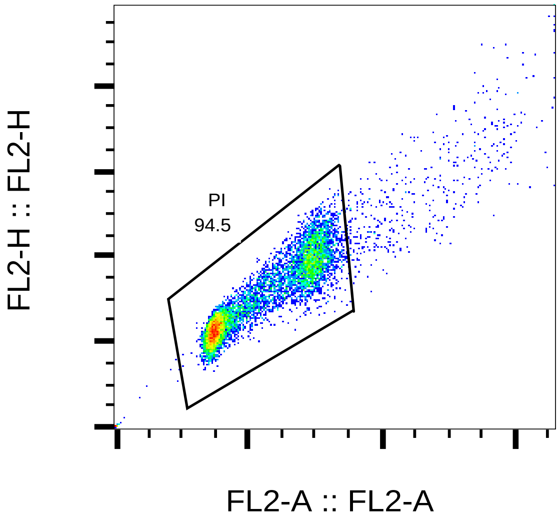

A04 14 shscramble.fcs
Single Cells 2

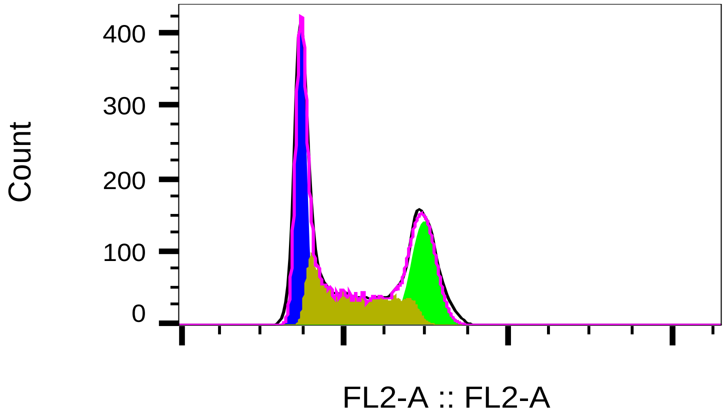

A04 14 shscramble.fcs
Cell Cycle

: 1.79
%G1 : 34.3
%S : 34.8
%G2 : 28.7
G1 Mean: 148789
G2 Mean: 298472
G1 CV: 5.97
G2 CV: 7.19
% less G1: -1.73
% greater G2: 3.61

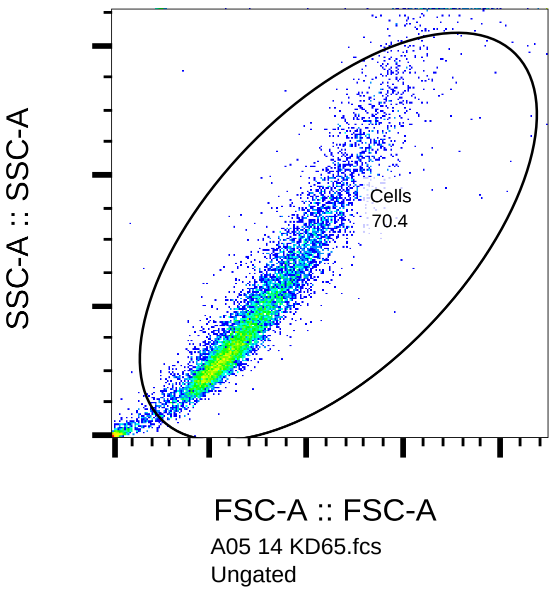

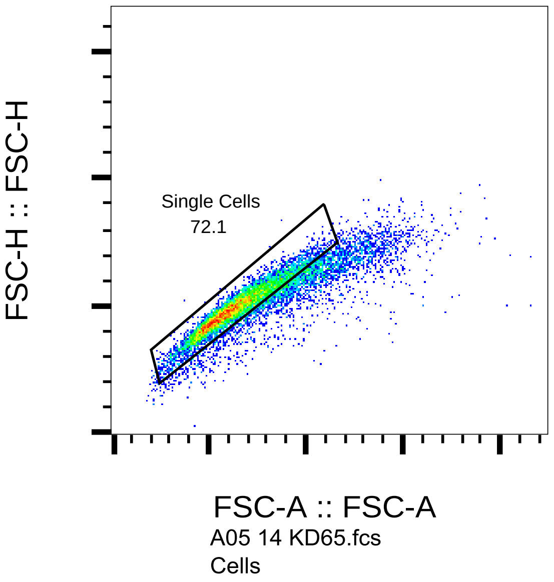

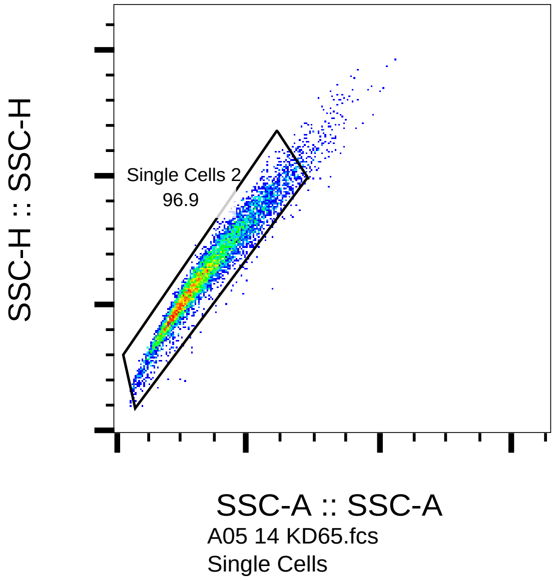

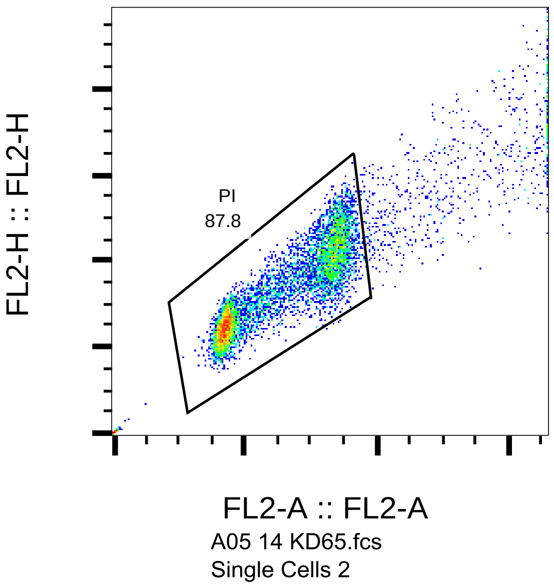

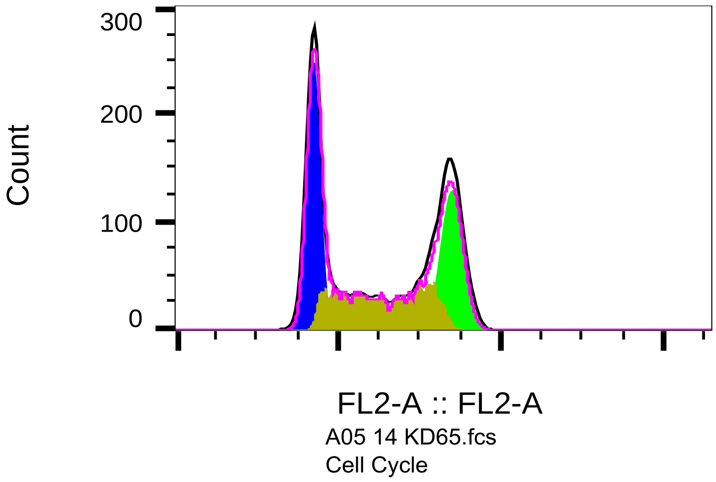

: 0.91
%G1 : 35.3
%S : 33.8
%G2 : 29.2
G1 Mean: 170331
G2 Mean: 340101
G1 CV: 6.99
G2 CV: 5.61
% less G1: 0.32
% greater G2: 1.86

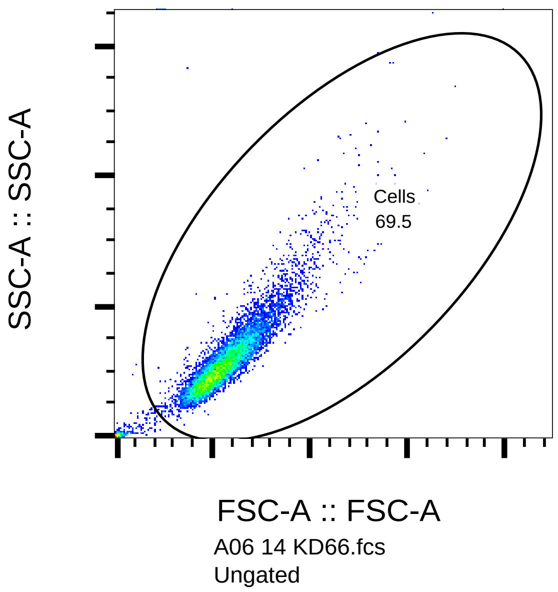

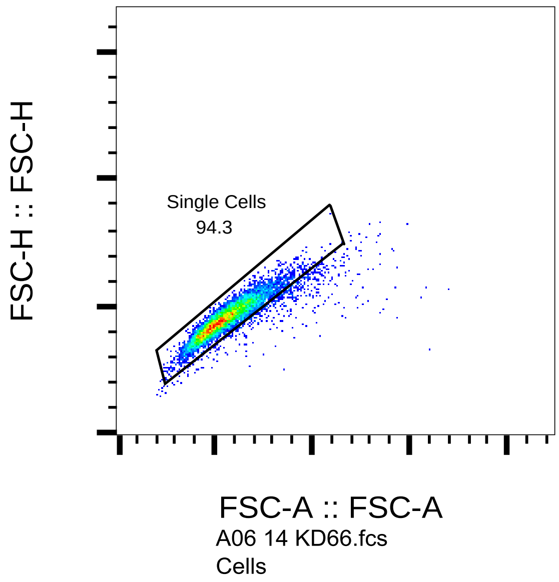

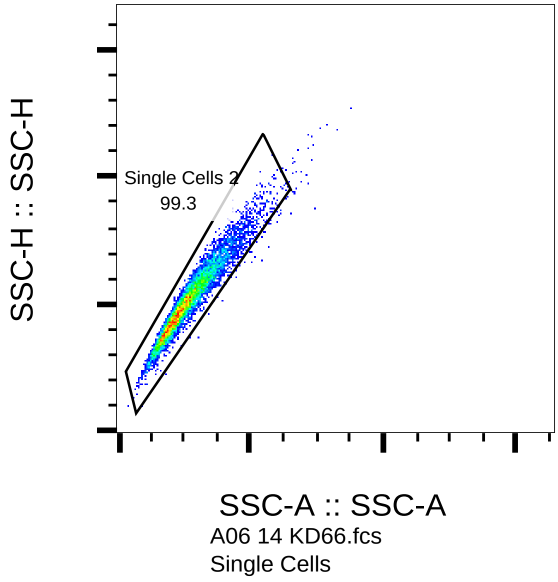

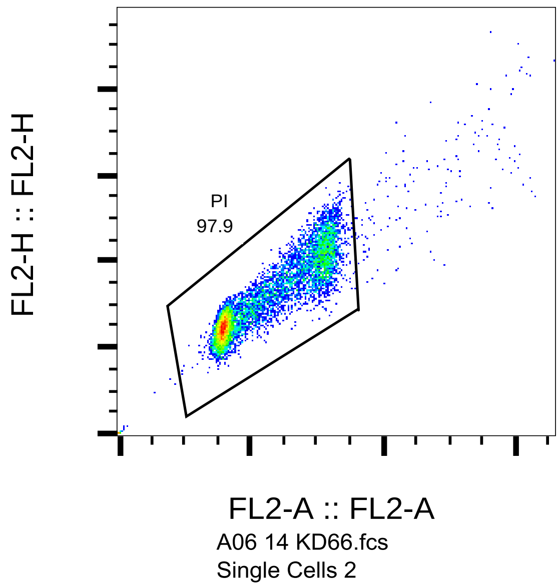

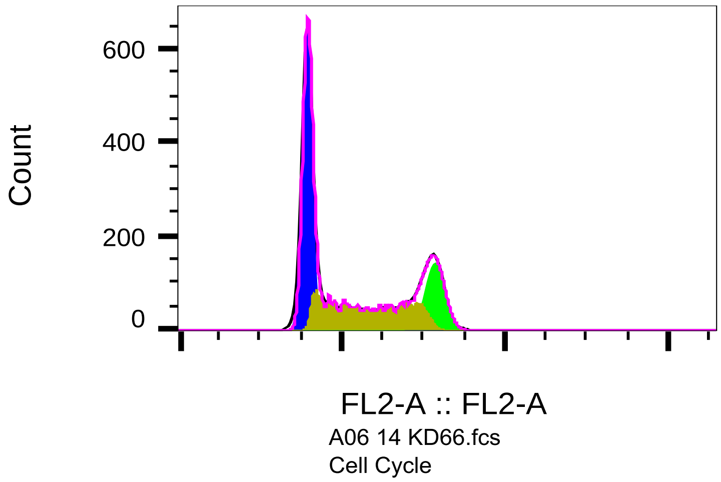

: 1.12
%G1 : 44.4
%S : 36.2
%G2 : 19.0
G1 Mean : 158774
G2 Mean : 314884
G1 CV : 5.37
G2 CV : 5.25
% less G1: 0.65
% greater G2: 0.14
